# A DFT Study on the Kinetics of HOO^•^, CH_3_OO^•^, and O_2_^•−^ Scavenging by Quercetin and Flavonoid Catecholic Metabolites

**DOI:** 10.3390/antiox12061154

**Published:** 2023-05-25

**Authors:** Ana Amić, Denisa Mastiľák Cagardová

**Affiliations:** 1 Department of Chemistry, Josip Juraj Strossmayer University of Osijek, Ulica cara Hadrijana 8A, 31000 Osijek, Croatia; 2 Institute of Physical Chemistry and Chemical Physics, Department of Chemical Physics, Slovak University of Technology in Bratislava, Radlinského 9, SK-812 37 Bratislava, Slovakia

**Keywords:** density functional theory (DFT), kinetics, quercetin, catecholic metabolites, alphitonin, 5-(3,4-dihydroxyphenyl)-γ-valerolactone, peroxyl radicals, superoxide, radical scavenging mechanisms

## Abstract

Reaction kinetics have been theoretically examined to ascertain the potency of quercetin (**Q**) and flavonoid catecholic metabolites **1**–**5** in the inactivation of HOO^•^, CH_3_OO^•^, and O_2_^•−^ under physiological conditions. In lipidic media, the $k_{\mathrm{overall}}^{\mathrm{TST}/\mathrm{Eck}}$ rate constants for the proton-coupled electron transfer (PCET) mechanism indicate the catecholic moiety of **Q** and **1**–**5** as the most important in HOO^•^ and CH_3_OO^•^ scavenging. 5-(3,4-Dihydroxyphenyl)-γ-valerolactone (**1**) and alphitonin (**5**) are the most potent scavengers of HOO^•^ and CH_3_OO^•^, respectively. The $k_{\mathrm{overall}}^{\mathrm{Mf}}$ rate constants, representing actual behavior in aqueous media, reveal **Q** as more potent in the inactivation of HOO^•^ and CH_3_OO^•^ via single electron transfer (SET). SET from 3-O^−^ phenoxide anion of **Q**, a structural motif absent in **1**–**5**, represents the most contributing reaction path to overall activity. All studied polyphenolics have a potency of O_2_^•−^ inactivation via a concerted two-proton–coupled electron transfer (2PCET) mechanism. The obtained results indicate that metabolites with notable radical scavenging potency, and more bioavailability than ingested flavonoids, may contribute to human health-promoting effects ascribed to parent molecules.

## 1. Introduction

Epidemiological and clinical studies support traditional knowledge that diets rich in fruit and vegetables have the potential to prevent or delay the development of various diseases [1]. The beneficial effects of such eating habits are partly related to polyphenols, widespread in the plant kingdom. The etiology of many diseases is related to cellular damage caused by the overproduction of free radicals in oxidative stress conditions when cell enzymatic defense mechanisms are not able to combat excess free radicals [2]. In this case, plant antioxidants, such as polyphenols, may help to restore homeostasis. Among the diverse possible mechanisms of the protective action of polyphenols, the direct scavenging of free radicals has been indicated as operative [3,4,5]; however, its bioefficacy was questioned [6,7]. Absorption and the blood concentration of polyphenols are very low when compared with their metabolites [1,8]. To be considered in vivo as a potentially active direct free radical scavenger, a polyphenolic compound must be bioavailable enough to reach a sufficiently high concentration in systemic circulation to fulfill such activity [3].

**Figure 1:**
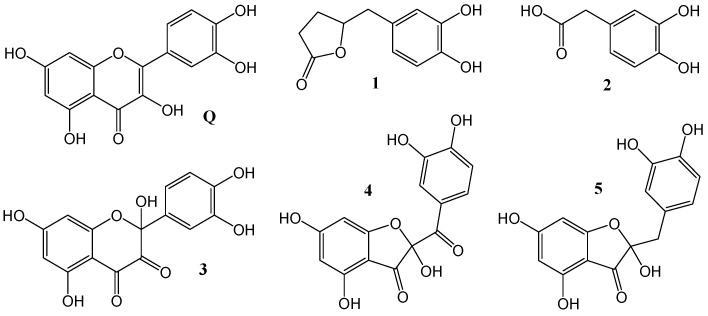
Studied catecholic compounds.

Catecholic flavonoids and their derivatives have been recognized as efficient free radical scavengers [9,10,11]. Among them, we selected six compounds for which high bioavailability and/or notable antioxidant activity has been found (Figure 1).

Catechins (flavan-3-ol monomers), constituents of green tea, are a subclass of flavonoids with numerous beneficial effects on human health [12]. After ingestion, they are partially absorbed in the small intestine and hence their concentration in human plasma may reach low μM values [13,14]. Following reduced absorption in the small intestine, considerable quantities of flavan-3-ol monomers pass to the colon where they are degraded by colonic microflora to phenolic catabolites such as 5-(3,4-dihydroxyphenyl)-γ-valerolactone **1** and 3,4-dihydroxyphenylacetic acid (DOPAC) **2** [15,16,17,18]. Bacterial catabolites appear to be more bioavailable and more abundant in the circulation than the parent compounds, thus increasing their importance for in vivo biological activities including free radical inactivation [3,5,19,20].

Quercetin **Q**, one of the most studied flavonoids, occurs ubiquitously as glycoside in fruit, vegetables, red wine, tea, and particularly in yellow and red onions [21]. Despite its generally poor bioavailability [22], a plethora of quercetin’s benefits on human health has been suggested including antioxidant, cardioprotective, anti-inflammatory, and anticancer activity [23,24]. It is also well known as an excellent in vitro free radical scavenger [10]. After ingestion, **Q** itself rarely exists unmodified in the living organism. Quercetin oxidation product 2,5,7,3′,4′-pentahydroxy-3,4-flavandione **3** or its tautomer 2-(3,4-dihydroxybenzoyl)-2,4,6-trihydroxy-3(2*H*)-benzofuranone **4** [25,26], which naturally occur in onions [27], and quercetin gut metabolite alphitonin **5** [28] were recognized as even more efficient antioxidants than quercetin itself [29,30,31,32,33]. The inhibitory activity of **Q** and **4** towards enzymes essential for SARS-CoV-2 has been recently investigated [34].

In this report, we used physiologically relevant hydroperoxyl radical (HOO^•^), its conjugated base superoxide anion radical (O_2_^•−^), and methylperoxyl radical (CH_3_OO^•^) as the lipid peroxyl radical model to investigate the protective potency of selected catecholic compounds against those important mediators of oxidative stress.

The deprotonation of HOO^•^ in aqueous systems results in the formation of O_2_^•−^:

HOO^•^ ⇄ H^+^ + O_2_^•−^

The p*K*_a_ of this equilibrium amounts to 4.8 which implies that at a physiological pH of 7.4, only 0.251% of the superoxide exists as a neutral species. O_2_^•−^ is a reactive oxygen species (ROS) ubiquitous in living systems, which is mainly produced by mitochondrial respiration [35]. O_2_^•−^ is predominantly present as a hydroperoxyl radical in biological membranes and as a superoxide anion radical in the aqueous phase. HOO^•^ is the simplest of the peroxyl radicals (ROO^•^) and is generally more reactive than O_2_^•−^. Peroxyl radicals can penetrate into lipid bilayers and react with allylic hydrogens of polyunsaturated fatty acids in membranes, i.e., they initiate lipid peroxidation which may cause destruction of the membrane function [36]. Although water-soluble but negligibly lipid-soluble O_2_^•−^ does not, in general, cross the membranes, it can pass through the anion exchange proteins [8]. O_2_^•−^, as an intrinsically weak oxidant, does not cause much direct damage to cells [37]. Deleterious effects on cells arise from the fact that O_2_^•−^ plays an important role as a precursor of other more oxidizing species formed in cellular systems, e.g., hydroxyl radical (HO^•^), hydrogen peroxide (H_2_O_2_), peroxynitrite (ONOO^−^), carbonate radical anion (CO_3_^•−^), and hypochlorite (^−^OCl) [38].

**Figure 2:**
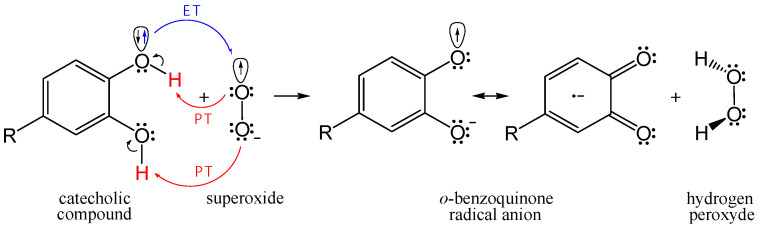
Simplified presentation for the 2PCET mechanism of O_2_^•−^ scavenging by the catecholic compound.

The main goal of this research is to theoretically compare the free radical scavenging potency of flavonoid metabolites **1**–**5** in reference to quercetin **Q**. To achieve this, we performed kinetic analysis of HOO^•^ and CH_3_OO^•^ scavenging via the proton-coupled electron transfer (PCET) mechanism in pentyl ethanoate (for neutral compounds) and via the single electron transfer (SET) mechanism in water (for phenoxide anion species of investigated compounds). The potency of the investigated catecholic compounds in O_2_^•−^ inactivation was estimated via a concerted two-proton–coupled electron transfer (2PCET) mechanism (Figure 2).

## 2. Materials and Methods

All electronic calculations were performed using the Gaussian 09 program package [39]. Geometry optimizations and frequency calculations for investigated compounds and their species involved in studied reactions were carried out using the M05-2X functional and the 6-311++G(d,p) basis set. The M05-2X has been chosen because it is one of the best-performing functionals for modeling reaction kinetics involving free radicals [40]. The M05-2X functional has been recommended for kinetic calculations by its developers, and it has been successfully used for the study of free radical scavenging mechanisms [41]. The popular B3LYP functional underestimates the barrier heights and is not appropriate for reaction kinetics [42]. The influence of solvents was calculated using an implicit continuum solvation model—SMD, which considers the full solute electron density in the estimation of the energy of the solvation [43]. SMD is a universal solvation model, and in conjunction with the M05-2X density functional, it represents a useful tool for the study of kinetics and thermodynamics of free radical inactivation [44]. Local minima and transition state (TS) were identified by the number of imaginary frequencies: local minima have only real frequencies, while TS is identified by the presence of a single imaginary frequency. An intrinsic reaction coordinate (IRC) calculation was performed on both sides of the TS to confirm that it properly connects two corresponding energy minima: reactant complex (RC) and product complex (PC). Further optimizations were carried out on the IRC final structures in order to obtain the fully relaxed geometries. All computations were performed at 298.15 K in water and pentyl ethanoate as solvents to mimic aqueous and lipid environments, respectively.

The rate constants (*k*) for PCET reactions were calculated in pentyl ethanoate using the conventional transition state theory (TST) [45] as implemented in the Eyringpy program [46]:

(1) $$k=\sigma \kappa \frac{k_{B}T}{h}e^{-\left(\Delta G^{\neq }\right)/RT}$$

where σ is the reaction path degeneracy, i.e., the number of different but equivalent reaction pathways that are possible, *k*_B_ is the Boltzmann constant, *T* is the temperature, *h* is the Planck constant, and Δ*G*^≠^ is the Gibbs free energy of activation of the studied reaction. κ represents the one-dimensional tunneling corrections: in the Eyringpy program it is calculated through the Eckart approach [47]. 

For the SET reactions in water, the Marcus theory was used [48]. It relies on the transition state formalism and allows calculating the barrier of any SET reaction from two thermodynamic parameters, the free energy of reaction, $\Delta G_{\mathrm{SET}}^{0}$, and the nuclear reorganization energy, λ:

(2) $$\Delta G_{\mathrm{SET}}^{\neq }\;=\;\frac{\lambda }{4}\;{\left(1+\frac{\Delta G_{\mathrm{SET}}^{0}}{\lambda }\right)}^{2}$$

(3) $$\lambda \;\approx \;\Delta E_{\mathrm{SET}}-\Delta G_{\mathrm{SET}}^{0}$$

$\Delta E_{\mathrm{SET}}$ is the nonadiabatic energy difference between reactants and vertical products for SET [49]. Accordingly, the TST rate constant (*k*_SET_) for SET reactions is computed in the Eyringpy program using the following equation:

(4) $$k_{\mathrm{SET}}=\frac{k_{B}T}{h}e^{-\left(\Delta G_{\mathrm{SET}}^{\neq }\right)/RT}$$

Some of the rate constants calculated using the conventional TST can be sometimes equal to or even higher than the diffusion-limited rate constant. In this case, the kinetics of the reaction are controlled by the rate at which reactants diffuse toward each other. To preserve a physical meaning, the reaction rate constant must be smaller than the diffusion limit [41]. For this reason, the apparent rate constant (*k*_app_), which is expected to reproduce the experimental findings, can be calculated according to the Collins–Kimball theory [50]:

(5) $$k_{\mathrm{app}}=\frac{k_{D}\;k}{k_{D}+k}$$

where *k* is the thermal rate constant, obtained from the TST calculations, and *k_D_* is the steady-state Smoluchowski rate constant for an irreversible bimolecular diffusion-controlled reaction [51], which is calculated as:

*k*_D_ = 4π*R*_AB_*D*_AB_*N*_A_ (6)

where *R*_AB_ is the reaction distance, *N*_A_ is the Avogadro constant, and *D*_AB_ is the mutual diffusion coefficient of the reactants A (free radical) and B (catecholic compound) [46].

For the SET mechanism, the rate constant (*k*^Mf^) involving molar fractions of reactants (antioxidant (M*f*_A–_) and radical species (M*f*_ROO•_)) at a given pH is directly related to experimentally determined ones under the same conditions:

*k*^Mf^ = *k*_app_ × ^M^*f*_A−_ × ^M^*f*_ROO•_ (7)

The overall rate constant ($k_{\mathrm{overall}}^{\mathrm{Mf}}$) can be estimated by summing up the rate constants for all phenoxide monoanionic paths:

(8) $$k_{\mathrm{overall}}^{\mathrm{Mf}}=\sum k_{\mathrm{Mf}}$$

The physiological pH = 7.4, M*f*_HOO•_ = 0.00251, M*f*_MeOO•_ = 1, and *k*_app_ denotes the related apparent rate constant. The molar fraction of the monoanionic species (^M^*f*_A–_) can be estimated from related p*K*_a_ values.

The branching ratios (Γ) calculated from the rate constants can be used to identify the reaction pathways most contributing to the total reaction (in %) [41]. They are calculated as:

(9) $$\Gamma =100\;\frac{k_{i}}{k_{\mathrm{overall}}}$$

where *k*_i_ represents the rate constant of an independent path. The overall rate constant (*k*_overall_) is calculated as the sum of the rate constants of all reaction paths.

## 3. Results and Discussion

### 3.1. PCET from OH Groups and SET from Phenoxide Anions of **Q** to HOO^•^ and CH_3_OO^•^ Radicals

**Figure 3:**
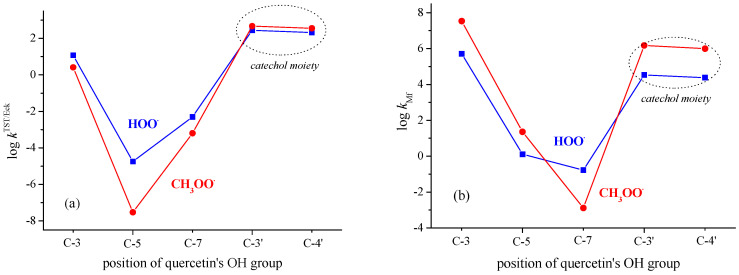
Scavenging of HOO^•^ and CH_3_OO^•^ radicals: (**a**) via PCET mechanism by OH groups of **Q** in non-polar media (pentyl ethanoate); (**b**) via SET mechanism by phenoxide monoanions of **Q** in polar media (water).

Very recently, a DFT kinetic analysis of mechanisms by which Q inactivates HOO^•^ and CH_3_OO^•^ radicals was performed by us [52]. Here, obtained results will be briefly outlined, and related new results will be presented. In non-polar media (pentyl ethanoate), the B-ring of **Q**, i.e., its catechol moiety, is the preferred site for scavenging of HOO^•^ and CH_3_OO^•^ radicals, as shown in Figure 3a. This is in agreement with an earlier experimental finding that the ring whose radical has a lower reduction potential is the antioxidant active moiety in any flavonoid [53], and with recent ESR measurements, which indicate that the unpaired electron of the quercetin radical is mostly delocalized in the B-ring and partly on the AC rings [54]. The estimated rate constant $k_{\mathrm{overall}}^{\mathrm{TST}/\mathrm{Eck}}$ (obtained without consideration of RC and PC) for HOO^•^ and CH_3_OO^•^ radical quenching amounts to 5.0 × 10^2^ M^−1^ s^−1^ and 8.3 × 10^2^ M^−1^ s^−1^, respectively. In both cases, the H-atom is transferred between two heteroatoms indicating the PCET mechanism as operative [55]. Consideration of the RC and PC has been shown to be essential for the determination of the barrier height and tunneling corrections [56]. By taking RC and PC into account, as well as the well-known fact that the Eckart method tends to overestimate the tunneling contributions, the recalculated rate constant $k_{\mathrm{overall}}^{\mathrm{TST}/\mathrm{Eck}}$ amounts to 1.1 × 10^3^ M^−1^ s^−1^ and 4.3 × 10^3^ M^−1^ s^−1^ for scavenging of HOO^•^ and CH_3_OO^•^ radicals (Table 1 and Table 2), respectively. Regarding the HOO^•^ radical (Chart S1), a linearly approximated *k*^TST/Eck^ value for the C3-OH site amounts to 5.0 × 10^1^ M^−1^ s^−1^ (instead of a highly overestimated value of 1.6 × 10^6^ M^−1^ s^−1^), which contributes to the more reliably predicted $k_{\mathrm{overall}}^{\mathrm{TST}/\mathrm{Eck}}$ = 1.1 × 10^3^ M^−1^ s^−1^.

Results for scavenging of HOO^•^ and CH_3_OO^•^ by **Q** via the SET mechanism in polar media are presented in Figure 3b. As expected [57,58], the SET mechanism in water, by which phenoxide anions of **Q** inactivate the HOO^•^ and CH_3_OO^•^ radicals, is much faster than the PCET mechanism in pentyl ethanoate.

In a polar aqueous environment, the main contribution to HOO^•^ and CH_3_OO^•^ scavenging by **Q** exerts the 3-O^−^ phenoxide anion followed by the catecholic moiety. Phenoxide monoanions of **Q** show higher potency in scavenging of CH_3_OO^•^ radicals in comparison with HOO^•^ radicals (Figure 3b). In the forthcoming sections, the obtained result for **Q** will be compared with the scavenging potency of flavonoid catecholic metabolites **1**–**5**.

### 3.2. PCET from OH Groups of **Q** and **1**–**5** to HOO^•^ and CH_3_OO^•^ Radicals in Non-Polar Media

Famous Bors’ criteria for efficient free radical scavenging indicate catecholic compounds as efficient free radical scavengers [9]. The results of numerous in vitro assays support the importance of this structural determinant for free radical quenching (for example, see [9,59,60]). The performed kinetic analysis revealed that potency of **Q** and catecholic derivatives **1**–**5** in scavenging of HOO^•^ radicals covers one order of magnitude: from 6.5 × 10^2^ M^−1^ s^−1^ for **2** to 3.06 × 10^3^ M^−1^ s^−1^ for **1** (Table 1). All this potency arises primarily from the contribution of the catecholic moiety. Contributions of 5-OH and 7-OH groups of **Q** and derivatives **3**–**5** are negligible. The role of the 3-OH group of **Q** is already specified.

**Figure 4:**
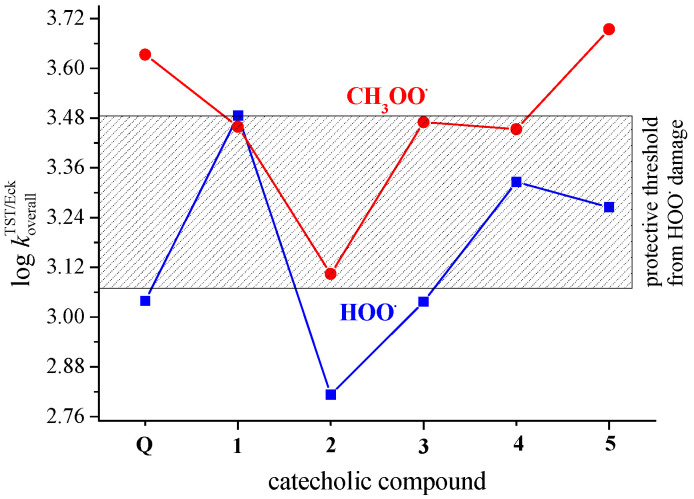
Scavenging of HOO^•^ and CH_3_OO^•^ radicals via the PCET mechanism by **Q** and catecholic derivatives **1**–**5** in non-polar media (pentyl ethanoate). Compounds above the shadowed rectangle have the potential to protect biological macromolecules from ROO^•^ damage. The protective potency decreases inside the rectangle and ceases below the threshold.

The experimentally measured rate constants corresponding to the reaction of HOO^•^ with polyunsaturated fatty acids (PUFAs) are in the range of 1.18–3.05 × 10^3^ M^−1^ s^−1^ [36]. Compounds that react faster with HOO^•^ than the double allylic hydrogens of the PUFAs are expected to act as efficient antioxidants [61]. The reactivity of biological targets such as proteins and DNA is lower than that of bisallylic hydrogens in PUFAs [62]. The shadowed rectangle in Figure 4 represents the abovementioned protective threshold of 1.18–3.05 × 10^3^ M^−1^ s^−1^, i.e., its log value of 3.08–3.48. Hence, the catecholic derivative **1**, with a log $k_{\mathrm{overall}}^{\mathrm{TST}/\mathrm{Eck}}$ value larger than the activity threshold, has the potential to protect biologically important molecules against HOO^•^-induced oxidative damage. The protective potency of catecholic derivatives **4** and **5** is reduced, while **Q**, **2**, and **3** appear as inactive. A higher efficiency of **4** than the parent **Q** molecule in scavenging of the DPPH^•^ radical was shown by an in vitro antiradical assay [29].

Similar to scavenging of the HOO^•^ radical, the potency of **Q** and compounds **1**–**5** in scavenging of the CH_3_OO^•^ radical ranges within one order of magnitude from 1.3 × 10^3^ M^−1^ s^−1^ for **2** to 4.9 × 10^3^ M^−1^ s^−1^ for the most potent catecholic derivative **5**, Table 2. For all studied compounds, the catecholic moiety is an active site for the scavenging of CH_3_OO^•^ radicals. The kinetic data presented here are in good agreement with the published theoretical results. For the reaction of the 4′-OH group of **Q** with CH_3_OO^•^ at the MPWB1K/6-311G** level of theory, the published TST rate constant amounts to 1.97 × 10^1^ M^−1^ s^−1^ [42]. By using the canonical variational transition state theory (CVT), corrected by the semiclassical multidimensional small-curvature tunneling (SCT) approach, the authors obtained a tunneling correction of κ_SCT_ = 496 and a rate constant of *k*^CVT/SCT^ = 9.63 × 10^3^ M^−1^ s^−1^ at 300 K.

The presented results indicate that among the studied catecholic derivatives **1**–**5**, quercetin gut catabolite **5** is a more potent scavenger of HOO^•^ and CH_3_OO^•^ radicals than **Q** itself, which is in accordance with the results of investigations of related biological activities (DPPH radical scavenging activity and α-glucosidase inhibitory activity [31]).

### 3.3. SET from Phenoxide Anions of **Q** and **1**–**5** to HOO^•^ and CH_3_OO^•^ Radicals in Polar Media

It is frequently the case that SET reactions are very fast and in cases within or close to the diffusion-limited regime the reliable rate constants cannot be directly obtained from the TST calculations [61]. Therefore, the TST rate constants (*k*^TST^) should be corrected to include the limit imposed by diffusion. Such a limit is represented by the diffusion-controlled rate constant (*k*_D_) that enables the calculation of the apparent rate constants *k*_app_, which is intended to reproduce the actual behavior in a real system [41].

The *k*_app_ rate constants are usually used in the literature to assess the peroxyl radical scavenging potency of (poly)phenolics [63]. For example, the results of a kinetic analysis related to the SET mechanism by which 4′-O^−^ phenoxide anions of **Q** and derivatives **3** and **4** inactivate HOO^•^ radicals were published recently [32]. The published *k*_app_ values are in good accordance with our results (Table 3): **Q** 1.2 × 10^7^ M^−1^ s^−1^ vs 1.4 × 10^7^ M^−1^ s^−1^; **3** 1.4 × 10^6^ M^−1^ s^−1^ vs. 1.1 × 10^6^ M^−1^ s^−1^; and **4** 4.8 × 10^2^ M^−1^ s^−1^ vs 2.7 × 10^2^ M^−1^ s^−1^, respectively. However, the authors of the abovementioned work only considered the *k*_app_ rate constant related to 4′-O^−^ phenoxide anions. Consequently, they did not recognize a major role of the 3-O^−^ phenoxide anion of **Q** and the 3′-O^−^ phenoxide anion of **3** and **4** in scavenging of HOO^•^ in an aqueous environment.

To calculate the rate constants (*k*_Mf_) that can be directly compared with the experimental ones, a crucial aspect is taking into account acid–base equilibria, i.e., the inclusion of molar fractions of reactants (catecholic compound and scavenged radical) at the pH of interest [64]. By considering the molar fractions of reactants at the physiological pH of 7.4, a more reliable picture of antiradical potency relevant to the human body arises. Such a calculated rate constant is directly related to an experimentally determined one. Surprisingly, the number of articles addressing this aspect is rather limited. To evaluate the *k*_Mf_, accurate, experimentally determined p*K*_a_ values are necessary. According to a study by Alvarez-Diduk et al. [65], the order of the first three deprotonation steps of Q is 4′-OH, 7-OH, and 3-OH, with the corresponding p*K*_a_ values of 6.41, 7.81, and 10.19, respectively. This implies that at the physiological pH of 7.4, the molar fractions (^M^*f*_A_) of **Q** species amount to the following: AH = 0.0686, A^−^ = 0.6702, A^2−^ = 0.2608, and A^3−^ = 0.0004. By including the molar fraction of HOO^•^ and A^−^ at pH = 7.4 (^M^*f*_HOO•_ = 0.00251; ^M^*f*_A–_ = 0.6702), and by summing up the ^M^*f*_HOO•_ × ^M^*f*_A–_ × *k*_app_, the $k_{\mathrm{overall}}^{\mathrm{Mf}}$ for the monoanions of **Q** was obtained, as shown in Table 3. While HOO^•^ presents an acid–base equilibrium, CH_3_OO^•^ does not. Thus a $k_{\mathrm{overall}}^{\mathrm{Mf}}$, related to CH_3_OO^•^ scavenging by phenoxide anions of **Q** and **1**–**5** (Table 4), embraces ^M^*f*_CH3OO•_ = 1.

Amorati et al. [21] indicated that a rate-determining reaction of **Q** with alkylperoxyl radicals must occur from equilibrated 3-O^−^, 4′-O^−^, and 7-O^−^ phenoxide monoanions by firstly involving the 3-O^−^ species. Our calculated rate constant for the reaction of **Q** with the hydroperoxyl radical in an aqueous environment at pH = 7.4 ($k_{\mathrm{overall}}^{\mathrm{Mf}}$ = 5.8 × 10^5^ M^−1^ s^−1^, Table 3) is in line with the experimentally determined one of 1.6 × 10^5^ M^−1^ s^−1^ [21] under the same conditions. 

For metabolite **2**, p*K*_a_’ values have been experimentally determined [66]. The carboxylate group (−COOH) of **2** is more acidic than its phenolic -OH groups. The p*K*_a_’s amount to 4.18, 9.42, and 11.65 for –COOH, 4-OH, and 3-OH, respectively, indicating that this metabolite primarily exists as a carboxylate anion (−COO^−^) in neutral solutions. More precisely, the molar fractions of **2** at the physiological pH of 7.4 are as follows: AH = 0.0006, A^−^ = 0.9931, A^2−^ = 0.0063, and A^3−^ = 5.34 × 10^−7^. By using *k*_app_ data listed in Table 3, the calculated $k_{\mathrm{overall}}^{\mathrm{Mf}}$ amounts to 1.2 × 10^5^ M^−1^ s^−1^.

To the best of our knowledge, there are no assayed p*K*_a_ values for the rest of the investigated metabolites, i.e., compounds **1** and **3**–**5**. The p*K*_a_ values predicted by using the ACD/pKa GALAS algorithm [67] are presented in Table S1. By using molar fractions listed in Table S2 and *k*_app_ values from Table 3, the $k_{\mathrm{overall}}^{\mathrm{Mf}}$ values for compounds **1** and **3**–**5** were calculated and are included in Table 3.

**Figure 5:**
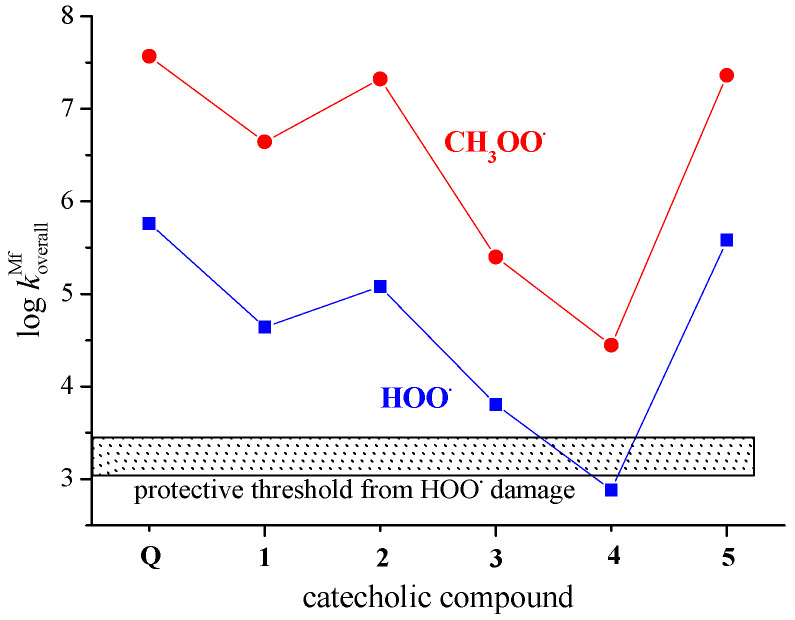
Scavenging of HOO^•^ and CH_3_OO^•^ radicals by phenoxide monoanions of **Q** and compounds **1**–**5** in polar media (water) via the SET mechanism as a function of the log $k_{\mathrm{overall}}^{\mathrm{Mf}}$.

The results presented in Table 3, Table 4, and Figure 5 indicate that **Q** has a higher potency than **1**–**5** in scavenging of both HOO^•^ and CH_3_OO^•^ radicals at pH = 7.4. This difference in reactivity partly arises due to the role of Q’s 3-O^−^ phenoxide anion in radical inactivation, a structural motif absent in **1**–**5**, and partly because of higher molar fractions of phenoxide monoanions of **Q** in comparison with that of **1**–**5** (Table S2). Figure 5 also clearly shows that in polar media via the SET mechanism, **Q** and catecholic metabolites **1**–**5** have the potential to protect biologically important molecules against peroxyl-induced oxidative damage because almost all of them overwhelm the protective threshold significantly. The only exception is phenoxide anion of **4**, a species below the protective threshold of 1.18–3.05 × 10^3^ M^−1^ s^−1^.

The graphically presented data in Figure 5 resemble each other indicating the scavenging of CH_3_OO^•^ by ~2.5 orders of magnitude faster than the scavenging of HOO^•^. The decrease in the $k_{\mathrm{overall}}^{\mathrm{Mf}}$ rate constants at pH = 7.4 for the reaction involving HOO^•^ is caused by the decrease in the radical abundance. The acid–base equilibrium of HOO^•^ in aqueous solution (p*K*_a_ = 4.8) is largely responsible for this decrease. The molar fraction of HOO^•^ at the pH of interest (at pH = 7.4 it amounts to 0.00251) must be included in the calculations, while it is ignored in the case of CH_3_OO^•^, which has no acid–base equilibria [63,64]. The trend of the presented data is in line with published results for the reactions between 3-hydroxyanthranilic acid and HOO^•^ and CH_3_OO^•^ radicals [64]. Thus, we can conclude that in an aqueous environment, CH_3_OO^•^ reacts via the SET mechanism faster with **Q** and **1**–**5** than the HOO^•^. A similar trend appears in lipid media via the PCET mechanism, as shown in Figure 4.

For both HOO^•^ and CH_3_OO^•^ radicals, monoanions of quercetin metabolites **3** and **4** exert the lowest antiradical potency. Recently, Fuentes et al. [30] reported that at nM concentrations, **3** and **4** revealed an antioxidant potency 200-fold higher than that of **Q**. The authors ascribed this potency to the ability of **3** and **4** to trigger immediate intracellular antioxidant responses, not related to radical scavenging. Catecholics **3** and **4** are potentially capable of activating the transcription factor Nrf2 pathway, such as **Q** itself [24]. The activation of Nrf2 may account for the upregulation of the expression of genes encoding ROS-removing and/or antioxidant-synthesizing enzymes [33]. To be able to exert direct free radical scavenging potency in vivo, a polyphenolic antioxidant should persist in circulation in high enough concentrations (μM to mM range), which is not a common circumstance [3,4,5].

To the best of our knowledge, there is no published experimental kinetic data for HOO^•^ and CH_3_OO^•^ scavenging by **Q** and **1**–**5**. The only results of the TEAC assay are available for **Q**, **1** and **2**. The data presented in Figure 5 indicate **Q** as being more potent in the scavenging of peroxyl radicals than **1** (a major microbial metabolite of proanthocyanidins) and its microbiota-derived metabolite **2**, which is in agreement with published experimental results of in vitro ABTS radical scavenging activity [10,68]. The assayed TEAC values of 4.7 mM, 1.4 mM, and 2.2 mM for **Q**, **1** and **2**, respectively, are in line with the predicted reactivity ranking.

As for **Q** [69], it has been demonstrated that **2** is characterized by the ability to inhibit the peroxidation of lipids, and possesses DPPH radical scavenging activity [70]. In μM concentrations, **2** inhibits lipid peroxidation induced by AAPH in rat plasma [71]. Nrf2 activation and prevention of the disruption of antioxidant enzymatic defenses by **2** seem to be involved in the mechanisms underlying antioxidant protection against cholesterol-triggered oxidative stress, rather than radical scavenging since lower concentrations were required for this process [72].

### 3.4. Concerted Two-Proton–Coupled Electron Transfer from Catechol Moiety of **Q** and **1**–**5** to O_2_^•−^

Natural polyphenolic antioxidants may exhibit a strong activity in scavenging of O_2_^•−^. Compounds with pyrogallol or catechol structural motifs were found to be the most rapid superoxide scavengers [73]. Some flavonoid’s colonic catabolites, which can be in situ produced by gut microbiota in high concentrations, possess catechol moiety, e.g., compound **2** [18]. In scavenging of O_2_^•−^ by the catechol moiety, the concerted 2PCET mechanism involving two-proton and one-electron transfers resulting in ortho-benzoquinone radical anion and H_2_O_2_ has been proposed by Nakayama and Uno [74,75,76,77]. The single electron transfer (ET) reaction from the catechol moiety to O_2_^•−^ occurs concertedly with the two-proton transfer (PT), as shown in Figure 2.

**Figure 6:**
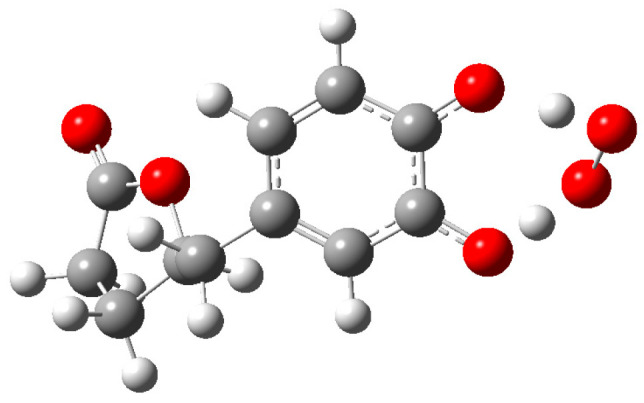
TS structure of the reaction of **1** with O_2_^•−^ in pentyl ethanoate.

The transition state for the reaction of inactivation of O_2_^•−^ by **Q** and derivatives **1**–**5** in pentyl ethanoate as a solvent was successfully achieved, as illustrated by Figure 6 for TS of **1**. The Cartesian coordinates of the investigated TSs are provided in the Supplementary Materials.

**Figure 7:**
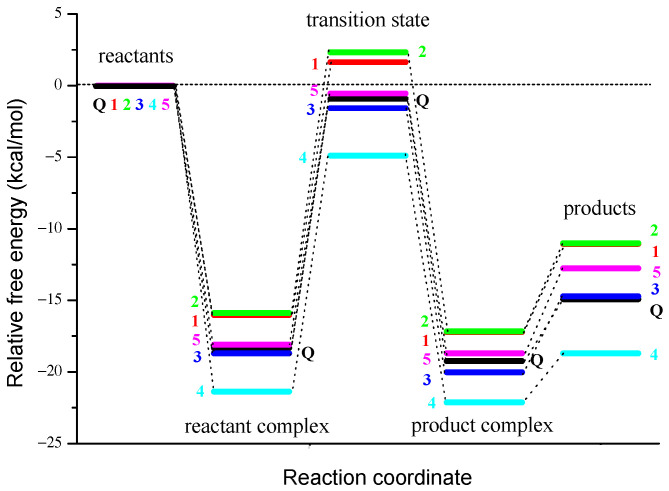
Reaction profiles for the concerted 2PCET from the catechol moiety of **Q** and **1**–**5** to O_2_^•−^ in pentyl ethanoate.

Our numerous attempts to reveal the transition state in water as a solvent were unsuccessful. Therefore, we present and discuss results obtained in non-polar media, i.e., in pentyl ethanoate. The results of TST kinetic calculations for the reaction of **Q** and derivatives **1**–**5** with O_2_^•−^ are summarized in Table 5. The occurrence of negative activation energy (Δ*G*^≠^ < 0 kcal/mol) in some 2PCET reactions can be explained by analyzing the corresponding reaction profiles shown in Figure 7.

As can be seen from the theoretical energy profile of the reaction of O_2_^•−^ with **Q** and **1**–**5**, the 2PCET mechanism proceeds via a hydrogen-bonded reactant complex, which is of much lower energy than the separated reactants, as shown in Figure 7. The consideration of the formation of the reactant complex (which is also called the pre-reactive complex, or van der Waals complex), a minimum along the reaction coordinate previous to the transition state, explains the negative value of the activation energy (transition state energy is lower than the energy of the separated reactants) observed for the reaction of O_2_^•−^ with **Q** and **3**–**5**. The point on the reaction profile corresponding to the formation of the stabilized reactant complex is especially important in radical-molecule reactions, many of which are known to occur with an apparent negative activation energy [56,78]. It should be noted that the phenomenon of a negative value of the activation energy has long been known [79] and even addressed in the popular undergraduate Atkins’ physical chemistry textbook [80].

The results presented in Table 5 indicate the 2PCET reaction of O_2_^•−^ with each of the studied catecholic compounds as diffusion controlled, with an assigned apparent rate constant *k*_app_ = 8.0–8.5 × 10^9^ M^−1^ s^−1^. Obviously, the reliability of this prediction can be estimated by comparison with the available experimental results. 

In the last three decades of the twentieth century, Bors’ research group has published several experimental reports on flavonoids acting as scavengers of O_2_^•−^ [9]. Pulse radiolysis combined with kinetic spectroscopy in aqueous solutions has been used to determine second-order rate constants. The group’s authors assumed that these constants are governed by the presence of a B-ring catechol group. The rate constant measured for O_2_^•−^ scavenging by **Q** amounts to 9.0 × 10^4^ M^−1^ s^−1^ [9]. The same research group determined a much higher rate constant of 2.3 × 10^7^ M^−1^ s^−1^ for the reaction of the catecholic compound adrenalone with O_2_^•−^ [81]. 

Rate constants for the reaction of O_2_^•−^ with a number of flavonoids and phenols were determined by pulse conductivity in aqueous solutions by Jovanovic et al. [82]. The measured rate constant for **Q** amounts to 4.7 × 10^4^ M^−1^ s^−1^, which is in good accordance with Bors’ result.

The highest experimental rate constant for the reaction of **Q** with O_2_^•−^ (1.0 × 10^6^ M^−1^ s^−1^) was determined by nonenzymatic chemiluminescent assay in aqueous solutions, an alternative to pulse radiolysis [73]. Our theoretically estimated rate constant for **Q** (*k*_app_ = 8.2 × 10^9^ M^−1^ s^−1^, Table 5) overwhelms this value by ~four orders of magnitude.

The reactivities of O_2_^•−^ with the biological molecules are of the order of ~1 s^−1^ or less [83]. The flavonoids are detected in plasma at levels in the range of 0.5–1.6 × 10^−6^ M [84]. If the *k* value obtained by Taubert et al. [73] is taken as reliable, then the concentration of **Q** in situ should be greater than 10^−5^ M for efficient in vivo scavenging of O_2_^•−^ (10^6^ M^−1^ s^−1^ × 10^−5^ M = 10 s^−1^). Such a local concentration could be expected for **Q** and polyphenol catecholic metabolites at specific sites of their accumulation [5,18,85]. Lipophilic flavonoids such as **Q** may accumulate in cell membranes. Their spatial confinement inside membranes greatly enhances their local concentration, thus increasing their importance for in vivo biological activities including radical scavenging [85].

## 4. Conclusions

The results of the performed theoretical kinetic analysis (estimated $k_{\mathrm{overall}}^{\mathrm{TST}/\mathrm{Eck}}$ rate constants) in non-polar media, where neutral forms of investigated compounds exist, indicate the catecholic moiety as operative in scavenging of HOO^•^ and CH_3_OO^•^ via the PCET mechanism. Some catecholic derivatives are more potent scavengers than **Q**: **1**, **4**, and **5** in the inactivation of HOO^•^, and **5** in the quenching of CH_3_OO^•^.

In polar media, which support the dissociation of phenolic OH groups, the phenoxide anions of the investigated compounds inactivated radicals via the SET mechanism. The obtained $k_{\mathrm{overall}}^{\mathrm{Mf}}$ rate constants, which include molar fractions of phenoxide anions and scavenged radicals, indicate **Q** as more potent than **1**–**5** in scavenging of HOO^•^ and CH_3_OO^•^.

Finally, our results indicate that the catecholic moiety of **Q** and derivatives **1**–**5** have the potency to inactivate O_2_^•−^ via the 2PCET mechanism.

Whether or not the predicted scenario could appear in vivo highly depends on the bioavailability and concentration of catecholic compounds in situ or in systemic circulation. It should be noted that a more complete picture of the antioxidant action of **Q** and **1**–**5** could be obtained by investigating interactions with prooxidant enzymes and catalytic metal ions.

## Figures and Tables

**Table 1:** Reaction Gibbs free energy (Δ_r_*G*, kcal/mol), TS imaginary frequency (*ν*, cm^−1^), Gibbs free energy of activation (Δ*G*^≠^, kcal/mol), TST rate constant (*k*^TST^, M^−1^ s^−1^), Eckart tunneling correction (*κ*^Eck^), and TST/Eckart rate constant (*k*^TST/Eck^, M^−1^ s^−1^) for the PCET reactions of **Q** and catecholic metabolites **1**–**5** with HOO^•^ in pentyl ethanoate at pH = 7.40 and 298.15 K.

Compound	Path	Δ_r_*G*	*ν*	Δ*G*^≠^	*k* ^TST^	*κ* ^Eck^	*k* ^TST/Eck^	$k_{\mathrm{overall}}^{\mathrm{TST}/\mathrm{Eck}}$
**Q**	C-3	−1.3	−4273.77	18.3	2.3 × 10^−1^	7,090,769.7	1.6 × 10^6^	1.1 × 10^3^
	C-5	12.7	−3894.44	27.2	7.8 × 10^−8^	318,443.4	2.5 × 10^−2^	
	C-7	8.1	−2521.38	23.3	5.2 × 10^−5^	1448.4	7.5 × 10^−2^	
	C-3’	−3.4	−1883.66	16.3	7.6 × 10^0^	84.8	6.4 × 10^2^	
	C-4’	−5.8	−1843.08	16.4	6.1 × 10^0^	64.8	4.0 × 10^2^	
**1**	C-3	−5.5	−1652.96	16.3	6.5 × 10^0^	25.1	1.6 × 10^2^	3.06 × 10^3^
	C-4	−6.6	−1644.00	14.7	1.0 × 10^2^	27.8	2.9 × 10^3^	
**2**	C-3	−5.3	−1674.57	16.1	1.0 × 10^1^	35.8	3.6 × 10^2^	6.5 × 10^2^
	C-4	−5.6	−1739.57	16.2	8.0 × 10^0^	36.1	2.9 × 10^2^	
**3**	C-5	13.3	−3018.24	29.0	3.4 × 10^−9^	22,708.0	7.7 × 10^−5^	1.09 × 10^3^
	C-7	24.2	−2496.43	25.4	1.4 × 10^−6^	1091.0	1.6 × 10^−3^	
	C-3’	−3.3	−1945.28	16.6	3.9 × 10^0^	194.1	7.5 × 10^2^	
	C-4’	−2.8	−1983.78	17.0	2.3 × 10^0^	150.5	3.4 × 10^2^	
**4**	C-5	27.3	−3936.12	25.8	7.4 × 10^−7^	433,548.2	3.2 × 10^−1^	2.12 × 10^3^
	C-7	24.7	−2362.23	24.7	5.0 × 10^−6^	829.8	4.2 × 10^−3^	
	C-3’	−2.5	−2108.97	16.5	5.2 × 10^0^	385.6	2.0 × 10^3^	
	C-4’	−2.2	−2142.87	18.2	2.6 × 10^−1^	454.2	1.2 × 10^2^	
**5**	C-5	27.3	−4228.32	24.8	4.4 × 10^−6^	1,026,701.2	4.5 × 10^0^	1.84 × 10^3^
	C-7	24.3	−2398.24	24.3	1.0 × 10^−5^	1040.3	1.1 × 10^−2^	
	C-3’	−4.2	−2289.25	15.6	2.2 × 10^1^	60.4	1.3 × 10^3^	
	C-4’	−5.1	−1813.86	16.3	7.1 × 10^0^	76.4	5.4 × 10^2^	

**Table 2:** Reaction Gibbs free energy (Δ_r_*G*, kcal/mol), TS imaginary frequency (*ν*, cm^−1^), Gibbs free energy of activation (Δ*G*^≠^, kcal/mol), TST rate constant (*k*^TST^, M^−1^ s^−1^), Eckart tunneling correction (*κ*^Eck^), and TST/Eckart rate constant (*k*^TST/Eck^, M^−1^ s^−1^) for the PCET reactions of **Q** and catecholic metabolites **1**–**5** with CH_3_OO^•^ in pentyl ethanoate at pH = 7.40 and 298.15 K.

Compound	Path	Δ_r_*G*	*ν*	Δ*G*^≠^	*k* ^TST^	*κ* ^Eck^	*k* ^TST/Eck^	$k_{\mathrm{overall}}^{\mathrm{TST}/\mathrm{Eck}}$
**Q**	C-3	0.9	−3359.24	22.7	1.4 × 10^−4^	198,437.3	2.8 × 10^1^	4.3 × 10^3^
	C-5	14.9	−2631.22	30.6	2.5 × 10^−10^	2359.1	5.8 × 10^−7^	
	C-7	10.4	−2455.59	23.3	4.9 × 10^−5^	281.5	1.4 × 10^−2^	
	C-3’	−1.1	−2226.66	16.3	7.2 × 10^0^	333.0	2.4 × 10^3^	
	C-4’	−3.5	−2259.20	16.5	4.7 × 10^0^	411.2	1.9 × 10^3^	
**1**	C-3	−3.2	−1919.02	16.7	3.4 × 10^0^	81.6	2.8 × 10^2^	2.88 × 10^3^
	C-4	−4.3	−2067.59	15.7	1.9 × 10^1^	137.2	2.6 × 10^3^	
**2**	C-3	−3.0	−1965.19	16.8	3.2 × 10^0^	105.2	3.4 × 10^2^	1.27 × 10^3^
	C-4	−3.4	−2124.71	16.5	5.3 × 10^0^	175.6	9.3 × 10^2^	
**3**	C-5	15.6	−2536.63	30.9	1.3 × 10^−10^	866.4	1.2 × 10^−7^	2.95 × 10^3^
	C-7	26.5	−2250.15	25.1	2.5 × 10^−6^	175.7	4.5 × 10^−4^	
	C-3’	−1.0	−2201.80	16.3	7.2 × 10^0^	363.0	2.6 × 10^3^	
	C-4’	−0.5	−2331.38	17.9	4.9 × 10^−1^	706.9	3.5 × 10^2^	
**4**	C-5	29.6	−2473.36	26.4	2.8 × 10^−7^	540.9	1.5 × 10^−4^	2.84 × 10^3^
	C-7	26.9	−2231.42	24.8	4.2 × 10^−6^	186.5	7.9 × 10^−4^	
	C-3’	−0.2	−2345.66	16.7	3.7 × 10^0^	711.6	2.6 × 10^3^	
	C-4’	0.1	−2364.37	18.3	2.3 × 10^−1^	1010.0	2.4 × 10^2^	
**5**	C-5	26.6	−2472.03	25.6	1.0 × 10^−6^	737.6	7.7 × 10^−4^	4.94 × 10^3^
	C-7	26.6	−2286.09	24.8	4.1 × 10^−6^	240.5	9.9 × 10^−4^	
	C-3’	−2.0	−2133.49	16.7	3.3 × 10^0^	284.1	9.4 × 10^2^	
	C-4’	−2.8	−2254.05	16.0	1.1 × 10^1^	349.2	4.0 × 10^3^	

**Table 3:** Reaction Gibbs free energy (Δ_r_*G*, kcal/mol), Gibbs free energy of activation (Δ*G*^≠^, kcal/mol), reorganization energy (λ, kcal/mol), diffusion rate constant (*k*_D_, M^−1^ s^−1^), apparent rate constant (*k*_app_, M^−1^ s^−1^), rate constant including molar fractions of radical and phenoxide anion (*k*^Mf^, M^−1^ s^−1^), and branching ratio (Γ, %) for the SET reactions of monoanion species of **Q** and **1**–**5** with HOO^•^, in water at pH = 7.40 and 298.15 K.

Compound	Path	Δ_r_*G*	Δ*G*^≠^	λ	*k*_D_	*k*_app_	*k* ^Mf^	Γ	$k_{\mathrm{overall}}^{\mathrm{Mf}}$
**Q**	3-O^−^	3.0	5.9	16.9	8.2 × 10^9^	3.1 × 10^8^	5.2 × 10^5^	90.3	5.8 × 10^5^
	5-O^−^	13.4	13.5	15.1	8.3 × 10^9^	8.0 × 10^2^	1.3 × 10^0^	0	
	7-O^−^	18.5	18.8	14.3	8.2 × 10^9^	1.0 × 10^−1^	1.7 × 10^−4^	0	
	3′-O^−^	5.8	7.5	16.2	8.2 × 10^9^	2.0 × 10^7^	3.4 × 10^4^	5.7	
	4′-O^−^	6.3	7.7	15.6	8.2 × 10^9^	1.4 × 10^7^	2.4 × 10^4^	4.0	
**1**	3′-O^−^	1.4	5.0	17.1	8.0 × 10^9^	1.1 × 10^9^	1.4 × 10^4^	31.5	4.4 × 10^4^
	4′-O^−^	0.6	4.4	16.6	7.9 × 10^9^	2.4 × 10^9^	3.00 × 10^4^	68.5	
**2**	3′-O^−^	0.2	4.3	16.9	7.9 × 10^9^	2.7 × 10^9^	4.3 × 10^4^	34.3	1.2 × 10^5^
	4′-O^−^	−1.5	3.6	17.2	7.8 × 10^9^	5.1 × 10^9^	8.0 × 10^4^	65.7	
**3**	5-O^−^	32.9	102.5	3.2	8.1 × 10^9^	4.6 × 10^−63^	5.1 × 10^−66^	0	6.4× 10^3^
	7-O^−^	33.6	36.7	18.4	8.1 × 10^9^	8.1 × 10^−15^	9.0 × 10^−18^	0	
	3′-O^−^	7.0	8.4	16.3	8.3 × 10^9^	4.7 × 10^6^	5.2 × 10^3^	81.0	
	4′-O^−^	8.2	9.2	16.3	8.2 × 10^9^	1.1 × 10^6^	1.2 × 10^3^	19.0	
**4**	7-O^−^	37.2	42.2	18.1	8.1 × 10^9^	7.2 × 10^−19^	9.1 × 10^−22^	0	7.6 × 10^2^
	3′-O^−^	8.5	9.6	17.1	8.2 × 10^9^	6.0 × 10^5^	7.6 × 10^2^	100	
	4′-O^−^	14.0	14.1	16.6	8.1 × 10^9^	2.7 × 10^2^	3.4 × 10^−1^	0	
**5**	5-O^−^	30.7	31.9	20.8	8.2 × 10^9^	2.8 × 10^−11^	3.1 × 10^−14^	0.0	3.8 × 10^5^
	7-O^−^	29.7	31.4	18.5	8.2 × 10^9^	6.5 × 10^−11^	7.2 × 10^−14^	0.0	
	3′-O^−^	3.8	6.4	17.1	8.2 × 10^9^	1.3 × 10^8^	1.4 × 10^5^	36.8	
	4′-O^−^	3.4	6.1	16.7	8.2 × 10^9^	2.2 × 10^8^	2.4 × 10^5^	63.2	

**Table 4:** Reaction Gibbs free energy (Δ_r_*G*, kcal/mol), Gibbs free energy of activation (Δ*G*^≠^, kcal/mol), reorganization energy (λ, kcal/mol), diffusion rate constant (*k*_D_, M^−1^ s^−1^), apparent rate constant (*k*_app_, M^−1^ s^−1^), rate constant including molar fractions of radical and phenoxide anion (*k*^Mf^, M^−1^ s^−1^), and branching ratio (Γ, %) for the SET reactions of monoanion species of **Q** and **1**–**5** with CH_3_OO^•^, in water at pH = 7.40 and 298.15 K.

Compound	Path	Δ_r_*G*	Δ*G*^≠^	λ	*k*_D_	*k*_app_	*k* ^Mf^	Γ	$k_{\mathrm{overall}}^{\mathrm{Mf}}$
**Q**	3-O^−^	4.9	6.9	16.5	7.7 × 10^9^	5.1 × 10^7^	3.4 × 10^7^	99.8	3.7 × 10^7^
	5-O^−^	15.4	15.4	14.7	7.8 × 10^9^	3.4 × 10^1^	2.3 × 10^1^	0	
	7-O^−^	20.4	21.2	13.9	7.8 × 10^9^	1.9 × 10^−3^	1.3 × 10^−3^	0	
	3′-O^−^	7.8	8.8	15.8	7.7 × 10^9^	2.3 × 10^6^	1.5 × 10^6^	0.1	
	4′-O^−^	8.3	9.0	15.2	7.7 × 10^9^	1.5 × 10^6^	1.0 × 10^6^	0.1	
**1**	3′-O^−^	3.3	6.0	16.7	7.6 × 10^9^	2.4 × 10^8^	1.2 × 10^6^	27.0	4.4 × 10^6^
	4′-O^−^	2.5	5.4	16.1	7.5 × 10^9^	6.5 × 10^8^	3.2 × 10^6^	73.0	
**2**	3′-O^−^	2.1	5.3	16.5	7.5 × 10^9^	7.9 × 10^8^	5.0 × 10^6^	24.0	2.1 × 10^7^
	4′-O^−^	0.4	4.4	16.7	7.5 × 10^9^	2.5 × 10^9^	1.6 × 10^7^	76.0	
**3**	5-O^−^	34.8	128.3	2.7	7.7 × 10^9^	5.3 × 10^−82^	2.3 × 10^−82^	0	2.5 × 10^5^
	7-O^−^	35.5	39.7	18.0	7.7 × 10^9^	4.8 × 10^−17^	2.1 × 10^−17^	0	
	3′-O^−^	8.9	9.7	15.9	7.8 × 10^9^	4.8 × 10^5^	2.1 × 10^5^	82.8	
	4′-O^−^	10.1	10.6	15.9	7.8 × 10^9^	1.0 × 10^5^	4.4 × 10^4^	17.2	
**4**	7-O^−^	39.1	45.6	17.7	7.7 × 10^9^	2.5 × 10^−21^	1.3 × 10^−21^	0	2.8 × 10^4^
	3′-O^−^	10.4	11.0	16.7	8.2 × 10^9^	5.5 × 10^4^	2.8 × 10^4^	100	
	4′-O^−^	15.9	15.9	16.2	8.1 × 10^9^	1.3 × 10^1^	6.5 × 10^0^	0	
**5**	5-O^−^	32.6	34.4	20.3	7.7 × 10^9^	3.6 × 10^−13^	1.6 × 10^−13^	0	2.3 × 10^7^
	7-O^−^	31.6	34.1	18.1	7.7 × 10^9^	6.4 × 10^−13^	2.8 × 10^−13^	0	
	3′-O^−^	5.7	7.5	16.6	7.7 × 10^9^	1.9 × 10^7^	8.4 × 10^6^	35.9	
	4′-O^−^	5.4	7.2	16.2	7.7 × 10^9^	3.4 × 10^7^	1.5 × 10^7^	64.1	

**Table 5:** Reaction Gibbs free energy (Δ_r_*G*, kcal/mol), TS imaginary frequency (*ν*, cm^−1^), Gibbs free energy of activation (Δ*G*^≠^, kcal/mol), TST rate constant (*k*^TST^, M^−1^ s^−1^), diffusion rate constant (*k*_D_, M^−1^ s^−1^), and apparent rate constant (*k*_app_, M^−1^ s^−1^) for reaction of **Q** and catecholic metabolites **1**–**5** with O_2_^•−^ in pentyl ethanoate at 298.15 K.

Compound	Δ_r_*G*	*ν*	Δ*G*^≠^	*k* ^TST^	*k*_D_	*k*_app_
**Q**	−14.9	−4882.49	−2.8	3.6 × 10^14^	8.2 × 10^9^	8.2 × 10^9^
**1**	−10.6	−5177.82	0.2	4.8 × 10^12^	8.2 × 10^9^	8.2 × 10^9^
**2**	−11.0	−4923.04	0.4	1.5 × 10^12^	8.0 × 10^9^	8.0 × 10^9^
**3**	−14.3	−5112.75	−3.1	1.1 × 10^15^	8.5 × 10^9^	8.5 × 10^9^
**4**	−18.7	−4798.18	−6.8	2.9 × 10^17^	8.5 × 10^9^	8.5 × 10^9^
**5**	−12.4	−5039.88	−2.0	1.9 × 10^14^	8.2 × 10^9^	8.2 × 10^9^

## Data Availability

Data are contained within the article and Supplementary Materials.

## Supplementary Materials

The following are available online at https://www.mdpi.com/article/10.3390/antiox12061154/s1: Chart S1: Estimation of the *k*^TST/Eck^ value for scavenging of HOO^•^ radical by the C3-OH site of Q; Table S1: p*K*_a_ values for **Q** and metabolites **1**–**5** calculated by using ACD/Percepta (2020) [67]. Available experimental results for **Q** and **2** are included [65,66]; Table S2: Molar fractions of **Q** and **1**–**5** species at pH = 7.40. Optimized geometries and Cartesian coordinates of TSs in PCET and 2PCET reactions.
