# Supplementary material for: A DFT Study on the Kinetics of HOO•, CH3OO•, and O2•− Scavenging by Quercetin and Flavonoid Catecholic Metabolites
Source: Antioxidants (Basel). 2023 May 25;12(6):1154. doi: 10.3390/antiox12061154 (PMC10295791; doi:10.3390/antiox12061154)
Supplement: Supplementary file 1 [file antioxidants-12-01154-s001.zip › antioxidants-2403132-supplementary.docx]

SUPPLEMENTARY INFORMATION

A DFT Study on the Kinetic of HOO^•^, CH_3_OO^•^, and O_2_^•−^ Scavenging by Quercetin and Flavonoid Catecholic Metabolites

Ana Amić^a*^, Denisa Mastiľák Cagardová^b^

^a^ Department of Chemistry, Josip Juraj Strossmayer University of Osijek, Ulica cara Hadrijana 8A, 31000 Osijek, Croatia

^b^ Institute of Physical Chemistry and Chemical Physics, Department of Chemical Physics, Slovak University of Technology in Bratislava, Radlinského 9, SK-812 37 Bratislava, Slovak Republic

E-mail: denisa.cagardova@stuba.sk

ORCID iD: 0000-0001-7784-1113 (Ana Amić); 0000-0002-6785-6570 (Denisa Cagardová)

* Author to whom correspondence should be addressed: Ana Amić

E-mail: aamic@kemija.unios.hr

**Chart S1** Estimation of the *k*^TST/Eck^ value for scavenging of HOO^•^ radical by the C3-OH site of **Q**

| path | Δ*G*^≠^ | log *k*^TST/Eck^ |
| --- | --- | --- |
| C-5 | 27.2 | -1.602 |
| C-7 | 23.3 | -1.125 |
| C-3' | 16.3 | 2.806 |
| C-4' | 16.4 | 2.602 |
|  | | |
| C-3 | 18.3 | 1.701 |

**Table S1** p*K*_a_ values for **Q** and metabolites **1**-**5** calculated by using ACD/Percepta (2020) [67]. Available experimental results for **Q** and **2** are included [65,66].

|  | p*K*_a1_ | p*K*_a2_ | p*K*_a3_ | p*K*_a4_ | p*K*_a5_ |  |
| --- | --- | --- | --- | --- | --- | --- |
| **Q** | 6.41 (4’-OH) | 7.81 (7-OH) | 10.19 (3-OH) | 11.53 (3’-OH) | 12.91 (5-OH) | [65] |
| **Q** | 7.7 (4’-OH) | 8.5 (7-OH) | 10.8 (3-OH) | 13.5 (5-OH) | 14.3 (3’-OH) | ACD |
| **1** | 9.7 (3-OH) | 12.6 (4-OH) | - | - | - | ACD |
| **2** | 4.18 (-COOH) | 9.42 (4-OH) | 11.65 (3-OH) | - | - | [66] |
| **2** | 4.4 (-COOH) | 10.3 (4-OH) | 14.1 (3-OH) | - | - | ACD |
| **3** | 7.5 (7-OH) | 9.9 (3’-OH) | 11.4 (5-OH) | 13.9 (4’-OH) | - (2-OH) | ACD |
| **4** | 7.3 (7-OH) | 8.1 (4’-OH) | 11.3 (5-OH) | 12.3 (3’-OH) | 13.8 (2-OH) | ACD |
| **5** | 7.5 (7-OH) | 10.0 (3’-OH) | 11.4 (5-OH) | 13.9 (4’-OH) | 15.9 (2-OH) | ACD |

65. Alvarez-Diduk, R.; Ramirez-Silva, M.T.; Galano, A.; Merkoci, A. Deprotonation mechanism and acidity constants in aqueous solution of flavonols: A combined experimental and theoretical study. *J. Phys. Chem. B* **2013**, *117*, 12347−12359.

66. Borges, F.; Guimaraes, C.; Lima, J.L.F.C.; Pinto, I.; Reis, S. Potentiometric studies on the complexation of copper(II) by phenolic acids as discrete ligand models of humic substances. *Talanta* **2005**, *66*, 670–673.

67. ACD/Percepta (2020). ACD/Labs Release 2020.2.0, https://www.acdlabs.com/products/percepta/predictors/pka/

**Table S2** Molar fractions of **Q** and **1**-**5** species at pH = 7.40.

|  | *f*[H_3_A] | *f*[H_2_A^−^] | *f*[HA^2−^] | *f*[A^3−^] |
| --- | --- | --- | --- | --- |
| **Q** | 0.06856 | 0.6702 | 0.2608 | 4.231 × 10^−4^ |
| **1** | 0.995012 | 0.004988 | 3.148 × 10^−8^ | - |
| **2** | 5.982 × 10^−4^ | 0.99307 | 6.285 × 10^−3^ | 5.336 × 10^−7^ |
| **3** | 0.557701 | 0.440900 | 0.001395 | 1.395 × 10^−7^ |
| **4** | 0.398308 | 0.501575 | 0.100104 | 1.261 × 10^−5^ |
| **5** | 0.556626 | 0.442263 | 0.001111 | 1.112 × 10^−7^ |

CARTESSIAN COORDINATES

Optimized geometry and Cartesian coordinates of quercetin (**Q**) 3’,4’-diOH∙∙∙O_2_^•−^ TS at SMD/um052x/6-311++g(d,p) level of theory in pentyl ethanoate

| 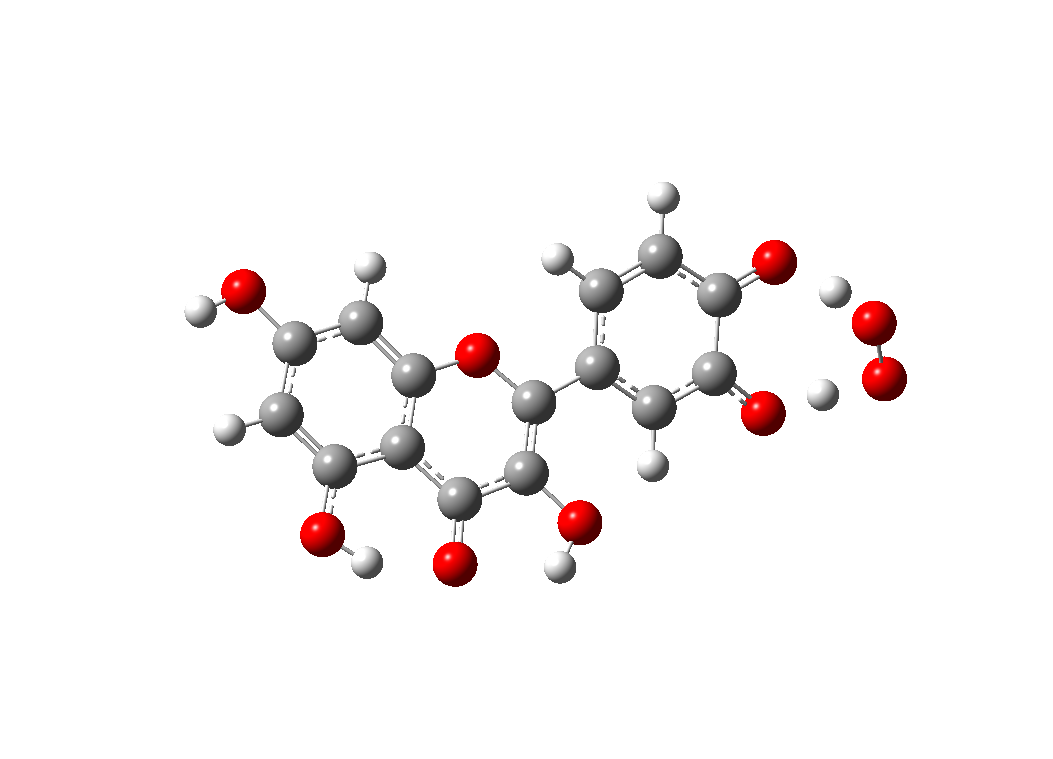 | 6 -1.482965000 -1.641264000 0.124454000  6 -2.777685000 -2.084629000 0.085707000  6 -3.867347000 -1.223172000 -0.226479000  6 -3.544803000 0.161299000 -0.525376000  6 -2.212940000 0.593963000 -0.445299000  6 -1.175319000 -0.279771000 -0.133209000  1 -0.688884000 -2.331272000 0.367543000  1 -3.014154000 -3.119445000 0.297099000  1 -2.023090000 1.631115000 -0.670175000  8 -4.467629000 0.998459000 -0.933228000  1 -5.493217000 0.919110000 -0.397841000  8 -5.076510000 -1.642693000 -0.244708000  8 -6.476898000 0.918319000 0.323242000  8 -6.164329000 -0.018370000 1.263266000  1 -5.767710000 -0.783583000 0.685039000  6 0.211554000 0.157275000 -0.081024000  6 0.689064000 1.433205000 -0.008916000  8 1.091235000 -0.887806000 -0.092014000  6 2.100821000 1.701572000 0.050900000  8 -0.115155000 2.521656000 0.029426000  6 2.428994000 -0.718096000 -0.028693000  6 2.975869000 0.565669000 0.037204000  8 2.500328000 2.885807000 0.125185000  1 0.483581000 3.281732000 0.103226000  6 3.217385000 -1.857765000 -0.038026000  6 4.382402000 0.702796000 0.099679000  6 4.593080000 -1.685281000 0.021506000  1 2.778219000 -2.842341000 -0.090498000  6 5.186649000 -0.419798000 0.088354000  8 4.936474000 1.919500000 0.169290000  8 5.351001000 -2.805451000 0.011133000  1 6.262164000 -0.308673000 0.132722000  1 4.202530000 2.575101000 0.170764000  1 6.283861000 -2.573121000 0.064696000 |
| --- | --- |

Note

Optimized geometry and Cartesian coordinates of TS’s related to reactions between quercetin’s 3,5,7,3’ and 4’ OH groups with ^•^OOH and ^•^OOCH_3_ radicals, at SMD/um052x/6-311++g(d,p) level of theory in pentyl ethanoate, are given in the Supplementary material of our recently published article:

[52] Amić, A.; Mastiľák Cagardová, D. DFT study of the direct radical scavenging potency of two natural catecholic compounds. *Int. J. Mol. Sci.* **2022**, *23*, 14497. https://doi.org/10.3390/

ijms232214497

Optimized geometry and Cartesian coordinates of 5-(3,4-dihydroxyphenyl)-γ-valerolactone (**1**)

3-OH∙∙∙^•^OOH TS at SMD/um052x/6-311++g(d,p) level of theory in pentyl ethanoate

| 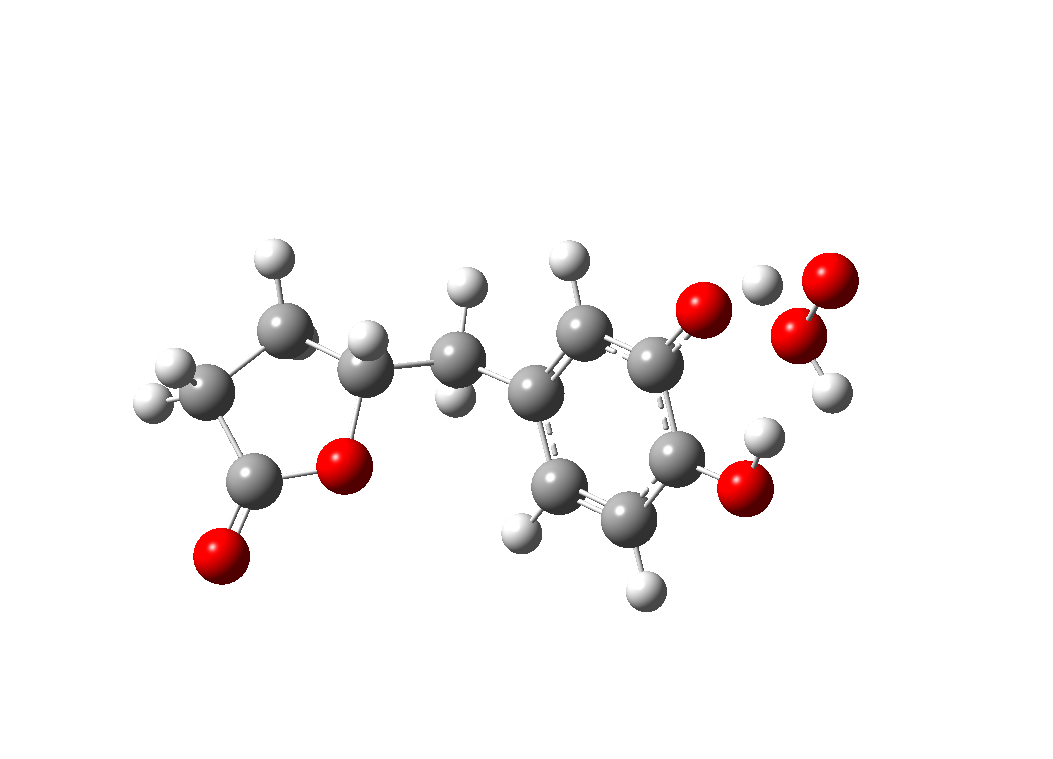 | 6 0.215129000 1.009028000 0.910517000  6 1.324870000 1.776908000 0.605164000  6 2.312328000 1.242191000 -0.213110000  6 2.185727000 -0.076881000 -0.709696000  6 1.048659000 -0.831508000 -0.387221000  6 0.058870000 -0.300561000 0.415336000  6 -1.182583000 -1.094717000 0.728534000  6 -2.332449000 -0.792794000 -0.214676000  6 -4.693961000 -0.695463000 -0.427723000  6 -4.117411000 0.669725000 -0.130634000  8 3.409157000 1.955439000 -0.524932000  1 -0.557162000 1.425779000 1.542566000  1 0.976743000 -1.838800000 -0.777602000  1 -1.516291000 -0.896056000 1.748717000  1 -0.966373000 -2.161612000 0.648731000  1 -1.999845000 -0.853778000 -1.252511000  1 3.910716000 1.449657000 -1.181240000  1 1.435165000 2.783367000 0.984119000  8 3.164304000 -0.534329000 -1.487509000  1 3.897662000 -1.029146000 -0.858452000  8 -2.778515000 0.574132000 0.006175000  6 -3.582177000 -1.639412000 0.014832000  1 -3.677233000 -1.867656000 1.077639000  1 -3.545481000 -2.570402000 -0.544778000  8 -4.694016000 1.715530000 -0.025968000  1 -5.645178000 -0.823957000 0.080364000  1 -4.866466000 -0.745418000 -1.505423000  8 3.861393000 -0.923544000 1.212403000  1 4.246346000 -0.082268000 1.509283000  8 4.648351000 -1.335831000 0.192411000 |
| --- | --- |

Optimized geometry and Cartesian coordinates of 5-(3,4-dihydroxyphenyl)-γ-valerolactone (**1**)

4-OH∙∙∙^•^OOH TS at SMD/um052x/6-311++g(d,p) level of theory in pentyl ethanoate

| 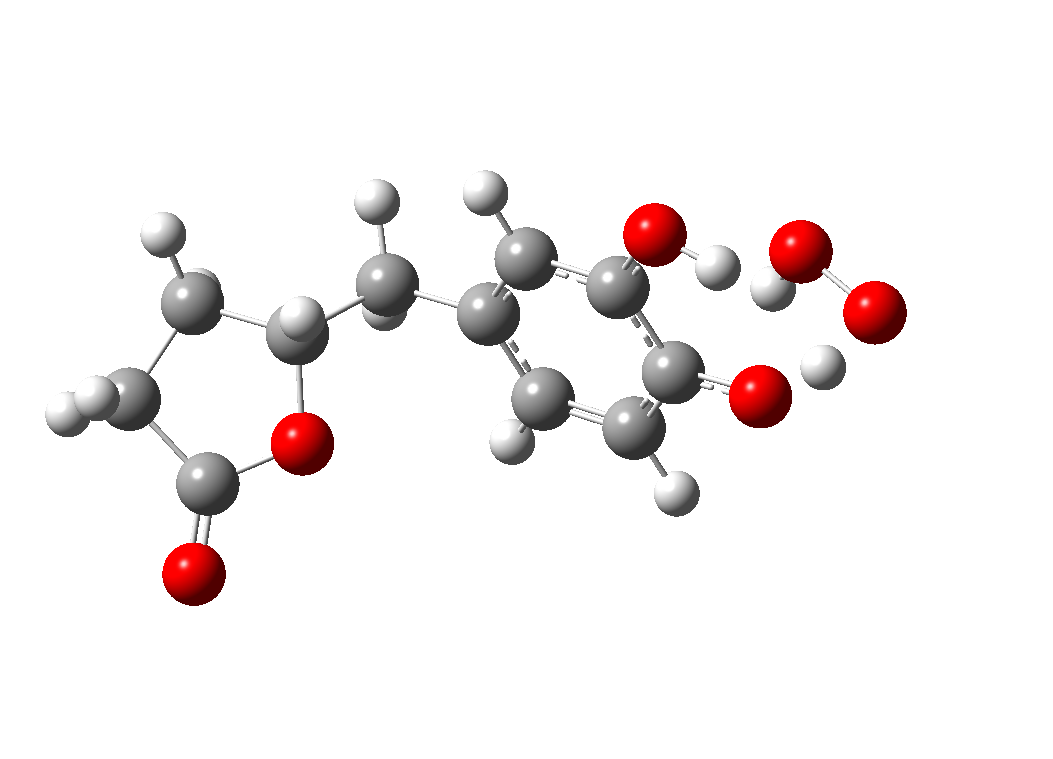 | 6 0.398156000 0.929240000 0.706159000  6 1.575010000 1.390875000 0.157755000  6 2.387276000 0.524499000 -0.591969000  6 1.975215000 -0.816940000 -0.784897000  6 0.787020000 -1.270275000 -0.229534000  6 -0.003807000 -0.405859000 0.517905000  6 -1.312275000 -0.889239000 1.082401000  6 -2.451642000 -0.770077000 0.084915000  6 -4.784901000 -0.398678000 -0.149966000  6 -4.004870000 0.879919000 -0.353000000  8 3.531437000 0.904887000 -1.153329000  1 -0.233985000 1.595983000 1.276498000  1 0.496484000 -2.301698000 -0.381686000  1 -1.575809000 -0.318242000 1.973921000  1 -1.231342000 -1.941510000 1.362104000  1 -2.178378000 -1.237134000 -0.862846000  1 4.298815000 0.906931000 -0.385108000  1 1.895012000 2.417097000 0.283142000  8 2.746219000 -1.641312000 -1.507217000  1 3.506323000 -1.130659000 -1.822468000  8 -2.687725000 0.637079000 -0.189245000  6 -3.795366000 -1.290919000 0.590781000  1 -3.867400000 -1.127055000 1.667078000  1 -3.919035000 -2.350875000 0.385016000  8 -4.420266000 1.970656000 -0.624907000  1 -5.712758000 -0.195444000 0.376713000  1 -5.026971000 -0.790630000 -1.140456000  8 4.068897000 -0.172310000 1.375724000  1 3.500918000 0.409275000 1.908205000  8 4.940092000 0.647085000 0.746047000 |
| --- | --- |

Optimized geometry and Cartesian coordinates of 5-(3,4-dihydroxyphenyl)-γ-valerolactone (**1**)

3-OH∙∙∙^•^OOCH_3_ TS at SMD/um052x/6-311++g(d,p) level of theory in pentyl ethanoate

| 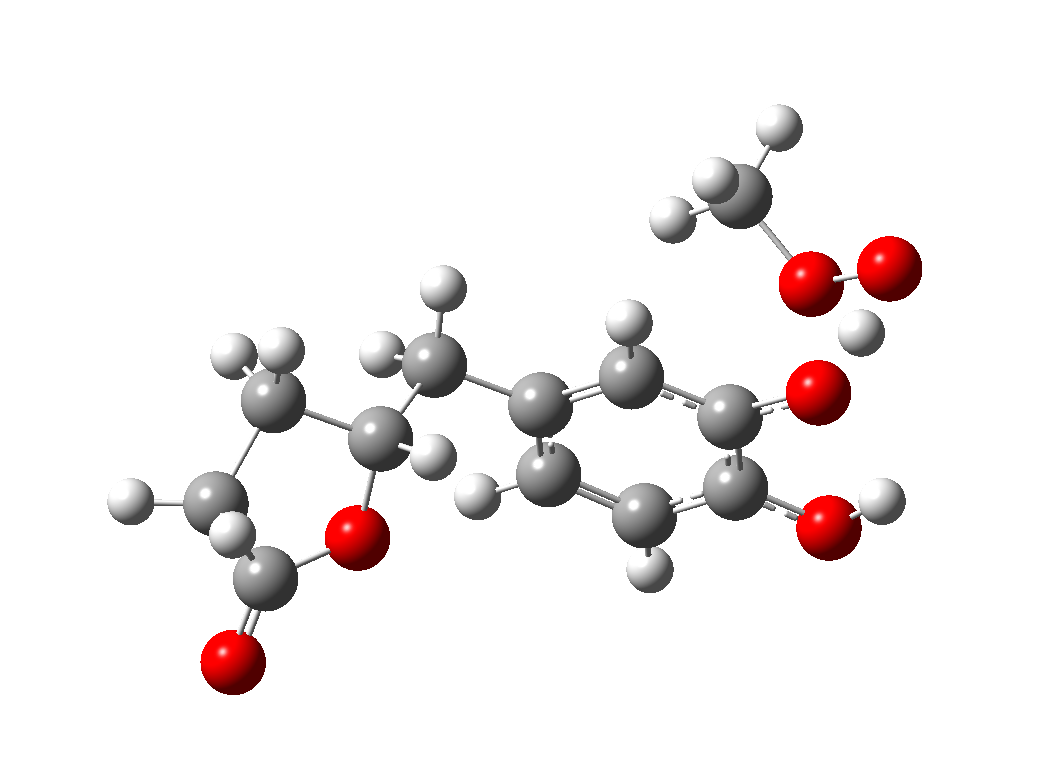 | 6 -0.009960000 0.849264000 -1.076570000  6 -1.074420000 1.711884000 -0.903969000  6 -2.027720000 1.430545000 0.070925000  6 -1.911899000 0.258733000 0.862012000  6 -0.815159000 -0.595867000 0.665605000  6 0.138428000 -0.312721000 -0.290298000  6 1.341785000 -1.200879000 -0.468887000  6 2.528556000 -0.757735000 0.367039000  6 4.896356000 -0.654523000 0.481445000  6 4.315945000 0.651645000 -0.010361000  8 -3.057071000 2.262525000 0.266936000  1 0.736590000 1.070802000 -1.827454000  1 -0.745510000 -1.481214000 1.285971000  1 1.648866000 -1.218965000 -1.516463000  1 1.097321000 -2.222207000 -0.171064000  1 2.233907000 -0.637692000 1.411203000  1 -3.593679000 1.914786000 0.993083000  1 -1.179949000 2.604704000 -1.504067000  8 -2.836459000 0.029249000 1.785123000  1 -3.731016000 -0.389815000 1.282719000  8 2.973569000 0.546612000 -0.097471000  6 3.763955000 -1.645928000 0.240544000  1 3.820014000 -2.049078000 -0.771897000  1 3.741881000 -2.469760000 0.949111000  8 4.894517000 1.663284000 -0.290787000  1 5.825802000 -0.872211000 -0.036462000  1 5.112594000 -0.532267000 1.545255000  8 -3.874501000 -0.573226000 -0.739582000  8 -4.575812000 -0.799840000 0.393018000  6 -3.377117000 -1.810837000 -1.266428000  1 -2.697509000 -1.536964000 -2.069763000  1 -2.859791000 -2.360928000 -0.483192000  1 -4.215961000 -2.389495000 -1.651215000 |
| --- | --- |

Optimized geometry and Cartesian coordinates of 5-(3,4-dihydroxyphenyl)-γ-valerolactone (**1**)

4-OH∙∙∙^•^OOCH_3_ TS at SMD/um052x/6-311++g(d,p) level of theory in pentyl ethanoate

| 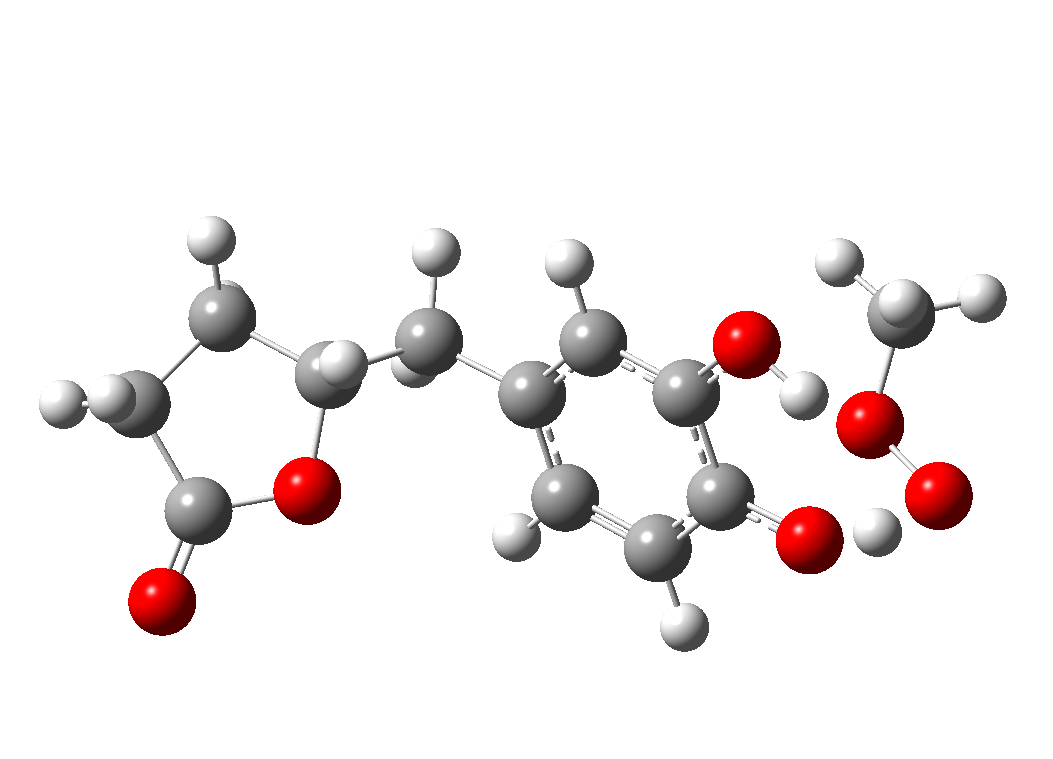 | 6 -0.102206000 0.878848000 -0.713420000  6 -1.241875000 1.471863000 -0.220448000  6 -2.075405000 0.765881000 0.663956000  6 -1.716229000 -0.552284000 1.042980000  6 -0.567895000 -1.142803000 0.538782000  6 0.244007000 -0.433356000 -0.339636000  6 1.516267000 -1.056904000 -0.846426000  6 2.703369000 -0.769356000 0.056509000  6 5.051946000 -0.470989000 0.153759000  6 4.336491000 0.858752000 0.085682000  8 -3.179227000 1.269814000 1.194195000  1 0.542660000 1.421793000 -1.390303000  1 -0.316392000 -2.152778000 0.836345000  1 1.750107000 -0.689465000 -1.846857000  1 1.402286000 -2.141393000 -0.899135000  1 2.458252000 -1.002681000 1.094274000  1 -3.965958000 1.272096000 0.400146000  1 -1.525695000 2.476931000 -0.502162000  8 -2.516146000 -1.218420000 1.892902000  1 -3.214573000 -0.604457000 2.166695000  8 3.005196000 0.651665000 0.002049000  6 4.003850000 -1.448021000 -0.365933000  1 4.046278000 -1.508991000 -1.454723000  1 4.089056000 -2.448992000 0.049101000  8 4.808598000 1.959932000 0.104068000  1 5.978163000 -0.432719000 -0.411902000  1 5.292744000 -0.654429000 1.203656000  8 -3.820550000 0.127834000 -1.273161000  8 -4.668370000 0.948452000 -0.614747000  6 -4.245403000 -1.231415000 -1.122761000  1 -3.479304000 -1.833896000 -1.604253000  1 -4.327555000 -1.477629000 -0.064862000  1 -5.207590000 -1.358916000 -1.616733000 |
| --- | --- |

Optimized geometry and Cartesian coordinates of 5-(3,4-dihydroxyphenyl)-γ-valerolactone (**1**)

3,4-diOH∙∙∙O_2_^•−^ TS at SMD/um052x/6-311++g(d,p) level of theory in pentyl ethanoate

| 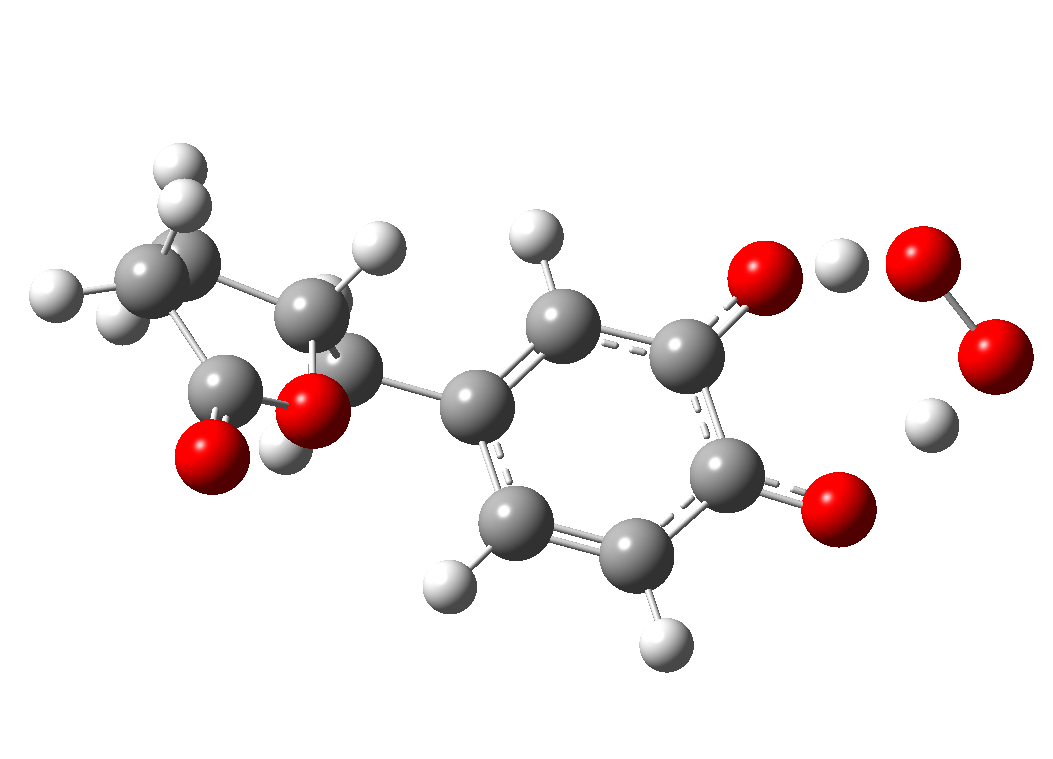 | 6 1.542774000 -0.808432000 -1.244408000  6 2.413932000 -0.736560000 -0.003929000  6 4.638645000 -0.610007000 0.813781000  6 3.993414000 0.756949000 0.770365000  1 2.054651000 -0.271984000 -2.046717000  1 1.487808000 -1.861008000 -1.532521000  1 1.868334000 -1.106712000 0.865464000  8 2.748464000 0.655140000 0.272860000  6 3.770756000 -1.424505000 -0.137458000  1 4.129791000 -1.323146000 -1.163211000  1 3.710717000 -2.480671000 0.113385000  8 4.462182000 1.807168000 1.114260000  1 5.689405000 -0.539824000 0.547082000  1 4.564036000 -0.970621000 1.842346000  6 0.150628000 -0.268529000 -1.039679000  6 -0.874809000 -1.109366000 -0.645690000  6 -0.129821000 1.102158000 -1.210913000  6 -2.184674000 -0.648446000 -0.429069000  1 -0.695870000 -2.170189000 -0.510015000  6 -1.396935000 1.594063000 -0.998385000  1 0.667265000 1.771388000 -1.509856000  6 -2.476847000 0.756649000 -0.607250000  8 -3.143927000 -1.494651000 -0.106054000  1 -1.614832000 2.645530000 -1.135837000  8 -3.665037000 1.233178000 -0.459680000  1 -3.630922000 -1.107383000 0.855880000  8 -4.133493000 -0.560865000 1.853826000  8 -5.066375000 0.249523000 1.274350000  1 -4.527282000 0.674829000 0.473306000 |
| --- | --- |

Optimized geometry and Cartesian coordinates of 3,4-dihydroxyphenylacetic acid (DOPAC) (**2**)

3-OH∙∙∙^•^OOH TS at SMD/um052x/6-311++g(d,p) level of theory in pentyl ethanoate

| 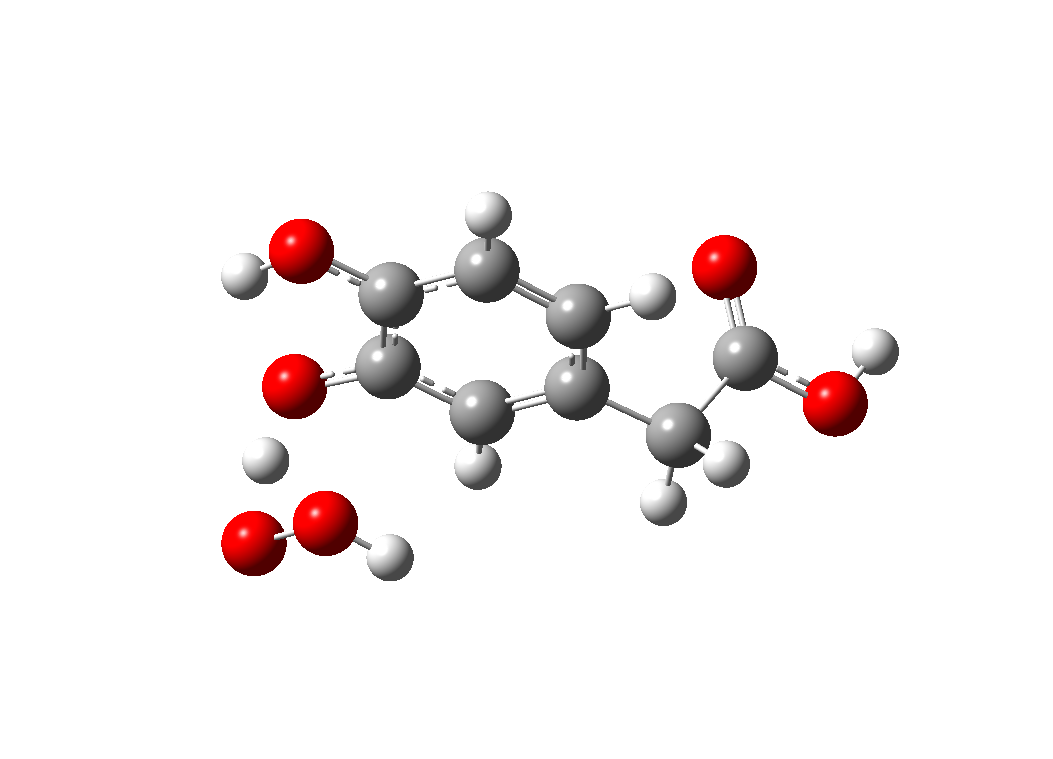 | 6 1.497708000 1.281341000 0.035231000  6 1.283719000 0.039430000 0.679605000  6 0.071558000 -0.644190000 0.483341000  6 -0.903561000 -0.114986000 -0.335635000  6 -0.668507000 1.121254000 -0.967501000  6 0.512127000 1.816215000 -0.788350000  1 -0.074923000 -1.586256000 0.997141000  1 -1.435341000 1.538533000 -1.607583000  1 0.688076000 2.764242000 -1.276951000  6 -2.208315000 -0.828099000 -0.545094000  6 -3.358521000 -0.180512000 0.184922000  1 -2.151731000 -1.863358000 -0.206784000  1 -2.477252000 -0.857074000 -1.603563000  8 -4.503602000 -0.847997000 -0.035451000  1 -5.208517000 -0.400793000 0.455192000  8 -3.304445000 0.798254000 0.878935000  8 2.248877000 -0.418679000 1.470427000  1 2.821314000 -1.156679000 0.913749000  8 2.651255000 1.931591000 0.227194000  1 3.179686000 1.416571000 0.854523000  8 2.753783000 -1.244411000 -1.157692000  1 1.936127000 -1.739045000 -1.332259000  8 3.324795000 -1.851117000 -0.092485000 |
| --- | --- |

Optimized geometry and Cartesian coordinates of 3,4-dihydroxyphenylacetic acid (DOPAC) (**2**)

4-OH∙∙∙^•^OOH TS at SMD/um052x/6-311++g(d,p) level of theory in pentyl ethanoate

| 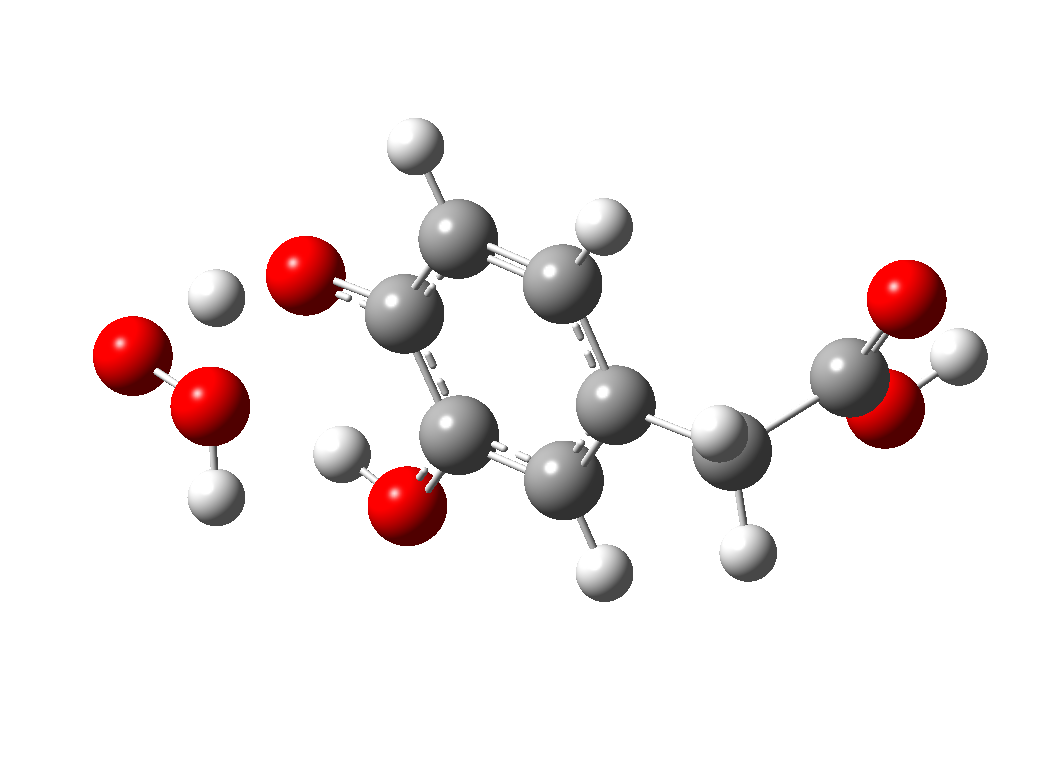 | 6 -1.447582000 -0.002100000 0.772505000  6 -0.950704000 1.080041000 0.005767000  6 0.286800000 0.993241000 -0.616795000  6 1.041035000 -0.162990000 -0.475900000  6 0.558349000 -1.240217000 0.290115000  6 -0.669894000 -1.163232000 0.905884000  1 0.650544000 1.832849000 -1.193485000  1 1.159250000 -2.135062000 0.387232000  1 -1.062519000 -1.982039000 1.493253000  6 2.405762000 -0.266632000 -1.114480000  6 3.479158000 -0.136357000 -0.061578000  1 2.541481000 0.525840000 -1.849004000  1 2.532975000 -1.234502000 -1.593315000  8 3.539796000 1.105915000 0.440130000  1 4.221211000 1.119590000 1.127849000  8 4.199771000 -1.023398000 0.312106000  8 -1.698620000 2.189096000 -0.115198000  1 -2.468701000 2.085831000 0.463498000  8 -2.628130000 0.159708000 1.359931000  1 -3.399730000 -0.291484000 0.732714000  8 -3.033724000 -1.003438000 -1.169411000  1 -3.010984000 -0.271020000 -1.806893000  8 -4.051727000 -0.710449000 -0.326928000 |
| --- | --- |

Optimized geometry and Cartesian coordinates of 3,4-dihydroxyphenylacetic acid (DOPAC) (**2**)

3-OH∙∙∙^•^OOCH_3_ TS at SMD/um052x/6-311++g(d,p) level of theory in pentyl ethanoate

| 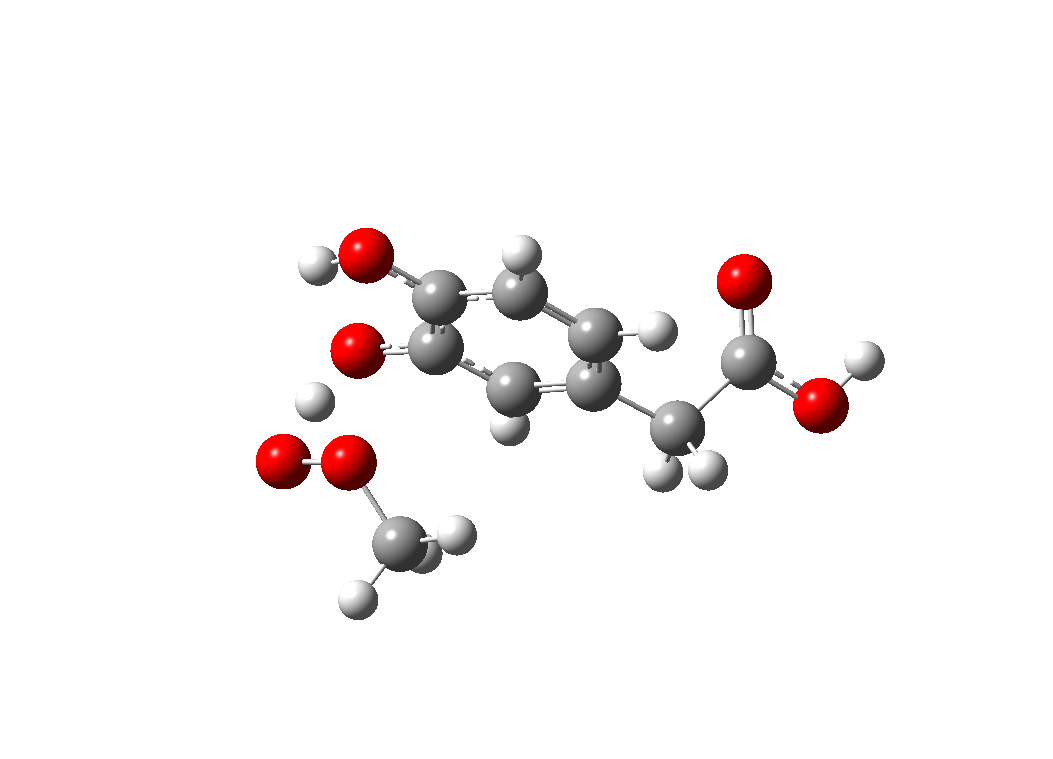 | 6 1.234611000 1.386578000 -0.162837000  6 1.041879000 0.392137000 0.829745000  6 -0.140993000 -0.367140000 0.819881000  6 -1.095296000 -0.166854000 -0.152377000  6 -0.869098000 0.809955000 -1.145212000  6 0.274580000 1.580717000 -1.154219000  1 -0.275298000 -1.109208000 1.596875000  1 -1.616321000 0.962428000 -1.913445000  1 0.440821000 2.335582000 -1.909955000  6 -2.362824000 -0.972267000 -0.168061000  6 -3.590941000 -0.158678000 0.155839000  1 -2.315917000 -1.794997000 0.545996000  1 -2.532573000 -1.419885000 -1.150609000  8 -4.688376000 -0.934278000 0.175747000  1 -5.447713000 -0.370149000 0.382393000  8 -3.632068000 1.022812000 0.367290000  8 1.979681000 0.234885000 1.752714000  1 2.802177000 -0.370440000 1.312608000  8 2.341396000 2.136825000 -0.142068000  1 2.871399000 1.871149000 0.623188000  8 2.875500000 -0.864016000 -0.656776000  8 3.554437000 -1.028645000 0.500219000  6 2.237054000 -2.091247000 -1.033169000  1 1.593551000 -1.841595000 -1.873448000  1 3.001843000 -2.806572000 -1.332652000  1 1.659645000 -2.476182000 -0.195492000 |
| --- | --- |

Optimized geometry and Cartesian coordinates of 3,4-dihydroxyphenylacetic acid (DOPAC) (**2**)

4-OH∙∙∙^•^OOCH_3_ TS at SMD/um052x/6-311++g(d,p) level of theory in pentyl ethanoate

| 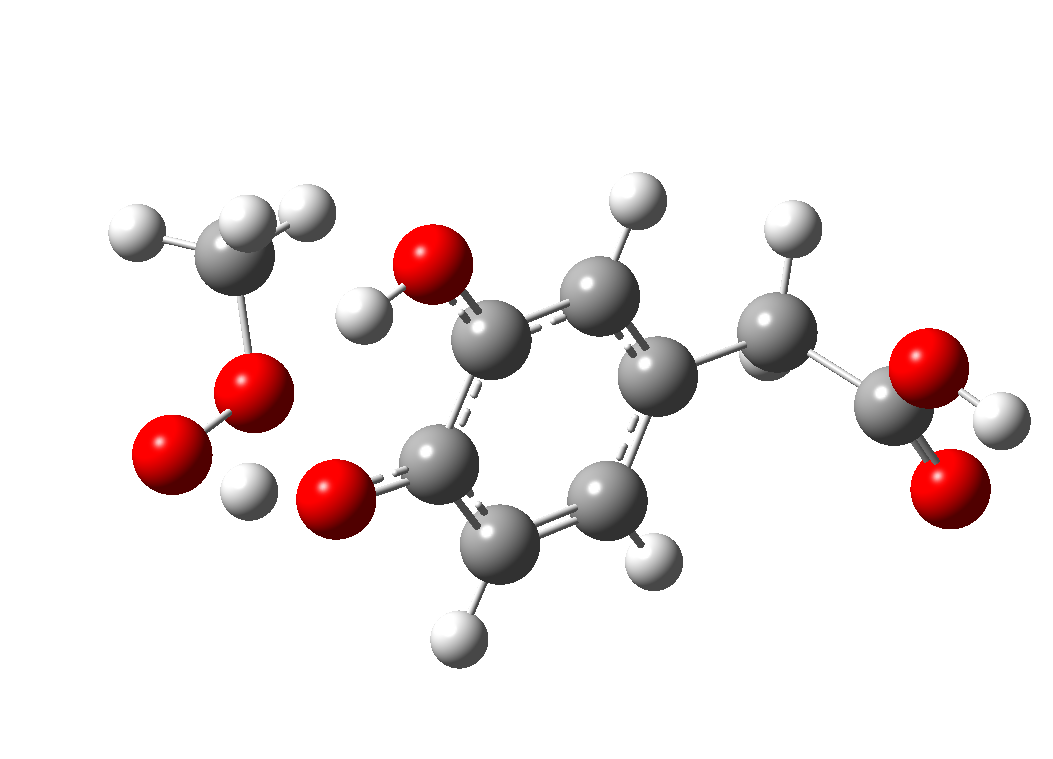 | 6 1.161568000 0.094734000 -1.013320000  6 0.707849000 1.145493000 -0.176124000  6 -0.480558000 1.026476000 0.530070000  6 -1.231923000 -0.133803000 0.406463000  6 -0.797358000 -1.179386000 -0.430609000  6 0.380300000 -1.068626000 -1.132402000  1 -0.808342000 1.841953000 1.160591000  1 -1.396612000 -2.076886000 -0.513266000  1 0.735424000 -1.864553000 -1.772825000  6 -2.540702000 -0.279375000 1.146528000  6 -3.690935000 -0.188361000 0.174476000  1 -2.645777000 0.510309000 1.888733000  1 -2.599653000 -1.249990000 1.633567000  8 -3.859642000 1.059516000 -0.286610000  1 -4.583456000 1.046480000 -0.929679000  8 -4.380546000 -1.108756000 -0.176897000  8 1.457232000 2.254464000 -0.075531000  1 2.196604000 2.155364000 -0.695398000  8 2.298128000 0.286266000 -1.661523000  1 3.095680000 -0.312127000 -1.137759000  8 2.835101000 -1.058791000 0.723964000  8 3.767678000 -0.897919000 -0.241362000  6 3.095100000 -0.160989000 1.809630000  1 2.276944000 -0.306881000 2.510295000  1 4.047879000 -0.424060000 2.266118000  1 3.117665000 0.865173000 1.445017000 |
| --- | --- |

Optimized geometry and Cartesian coordinates of 3,4-dihydroxyphenylacetic acid (DOPAC) (**2**)

3,4-diOH∙∙∙O_2_^•−^ TS at SMD/um052x/6-311++g(d,p) level of theory in pentyl ethanoate

| 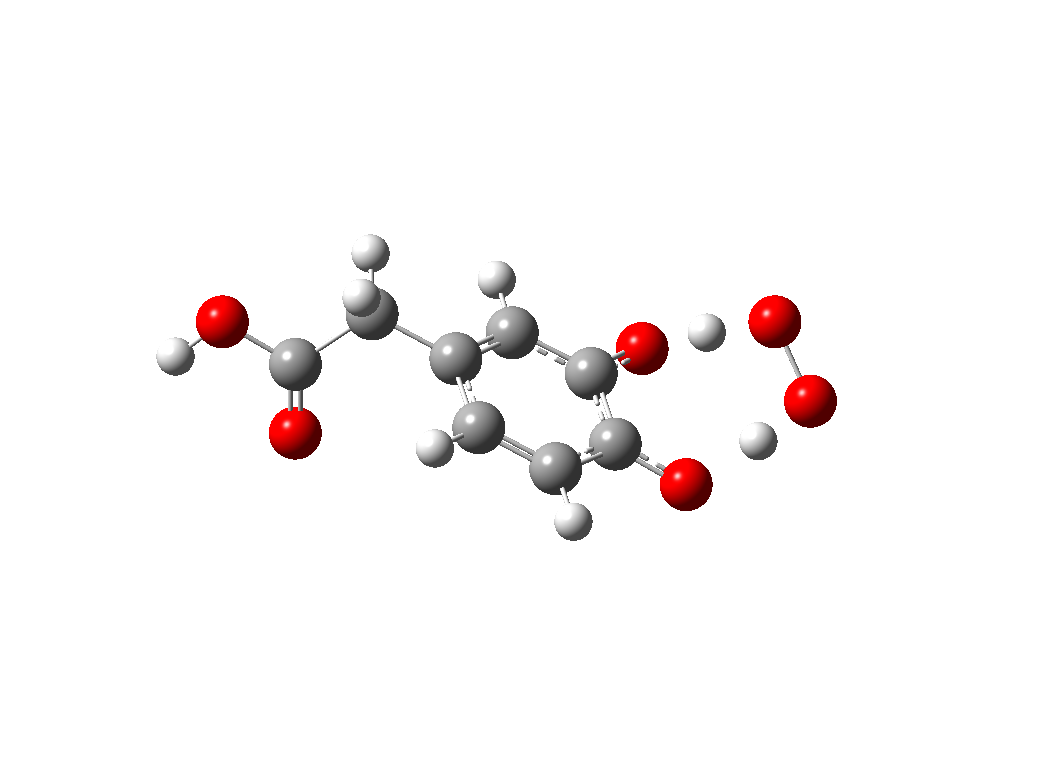 | 6 1.446281000 0.896418000 0.149965000  6 1.106349000 -0.498498000 0.315883000  6 -0.191857000 -0.933607000 -0.006746000  6 -1.165533000 -0.065939000 -0.459950000  6 -0.841351000 1.296224000 -0.619436000  6 0.419648000 1.757787000 -0.326899000  1 -0.407554000 -1.987709000 0.121867000  1 -1.599249000 1.984381000 -0.975366000  1 0.671444000 2.803871000 -0.445946000  6 -2.542609000 -0.561109000 -0.798460000  6 -3.606544000 -0.156226000 0.188550000  1 -2.564144000 -1.651360000 -0.852641000  1 -2.867525000 -0.199002000 -1.776993000  8 -4.823621000 -0.578676000 -0.213234000  1 -5.462777000 -0.306574000 0.460099000  8 -3.453604000 0.454199000 1.211397000  8 1.995867000 -1.351227000 0.786820000  8 2.612560000 1.357507000 0.443054000  1 3.738094000 0.557436000 0.263691000  8 4.527076000 -0.084519000 -0.009617000  8 3.870837000 -1.087144000 -0.662934000  1 2.912485000 -1.292457000 0.103960000 |
| --- | --- |

Optimized geometry and Cartesian coordinates of 2,5,7,3′,4′-pentahydroxy-3,4-flavandione (**3**)

5-OH∙∙∙^•^OOH TS at SMD/um052x/6-311++g(d,p) level of theory in pentyl ethanoate

| 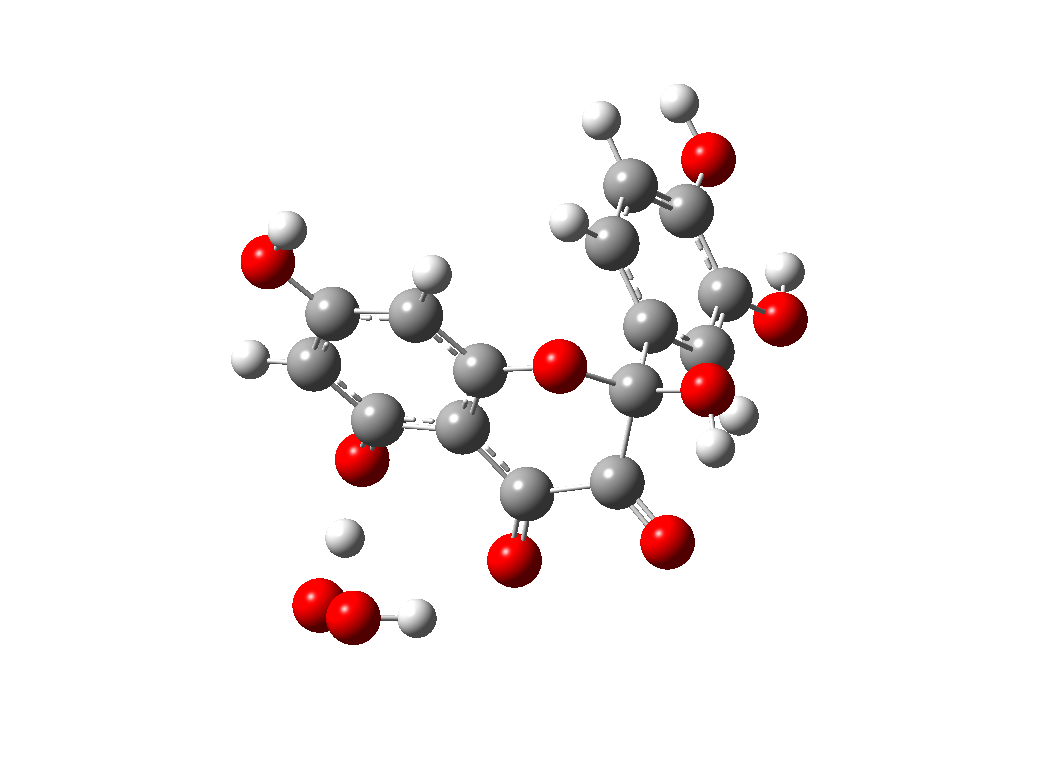 | 6 -1.049192000 0.738437000 1.114798000  6 -1.358561000 -0.079541000 0.021808000  6 -1.640003000 1.988400000 1.282571000  6 -2.194024000 0.467259000 -1.018069000  6 -2.518033000 2.465102000 0.312678000  6 -2.779534000 1.729880000 -0.838222000  6 0.849306000 -0.581454000 1.737448000  6 0.123375000 -1.783649000 1.133395000  8 -0.185617000 0.367674000 2.090816000  6 -0.911446000 -1.456227000 0.040565000  6 1.812446000 0.005418000 0.724188000  6 1.908412000 1.373034000 0.511759000  6 2.653710000 -0.876360000 0.045150000  6 2.850278000 1.864802000 -0.386537000  6 3.588615000 -0.387380000 -0.848271000  6 3.682967000 0.991259000 -1.062999000  8 -2.347432000 -0.137132000 -2.154106000  8 -3.116910000 3.663139000 0.427096000  8 -1.345219000 -2.372471000 -0.630577000  8 0.305799000 -2.902726000 1.513310000  8 1.517276000 -0.915608000 2.883712000  8 4.633189000 1.372381000 -1.961555000  8 4.406640000 -1.241471000 -1.507205000  1 -1.394155000 2.570593000 2.161430000  1 -3.410371000 2.132592000 -1.617146000  1 1.272208000 2.063347000 1.044814000  1 2.610781000 -1.945612000 0.210488000  1 2.938759000 2.929924000 -0.561031000  1 -3.139732000 -1.070867000 -2.157564000  1 -2.878448000 4.085437000 1.260195000  1 1.098218000 -1.686721000 3.288420000  1 4.641574000 2.328919000 -2.070387000  1 4.982314000 -0.735076000 -2.092495000  8 -4.046351000 -1.950989000 -0.635713000  1 -3.195865000 -2.399435000 -0.460472000  8 -4.025773000 -1.761169000 -1.979255000 |
| --- | --- |

Optimized geometry and Cartesian coordinates of 2,5,7,3′,4′-pentahydroxy-3,4-flavandione (**3**)

7-OH∙∙∙^•^OOH TS at SMD/um052x/6-311++g(d,p) level of theory in pentyl ethanoate

| 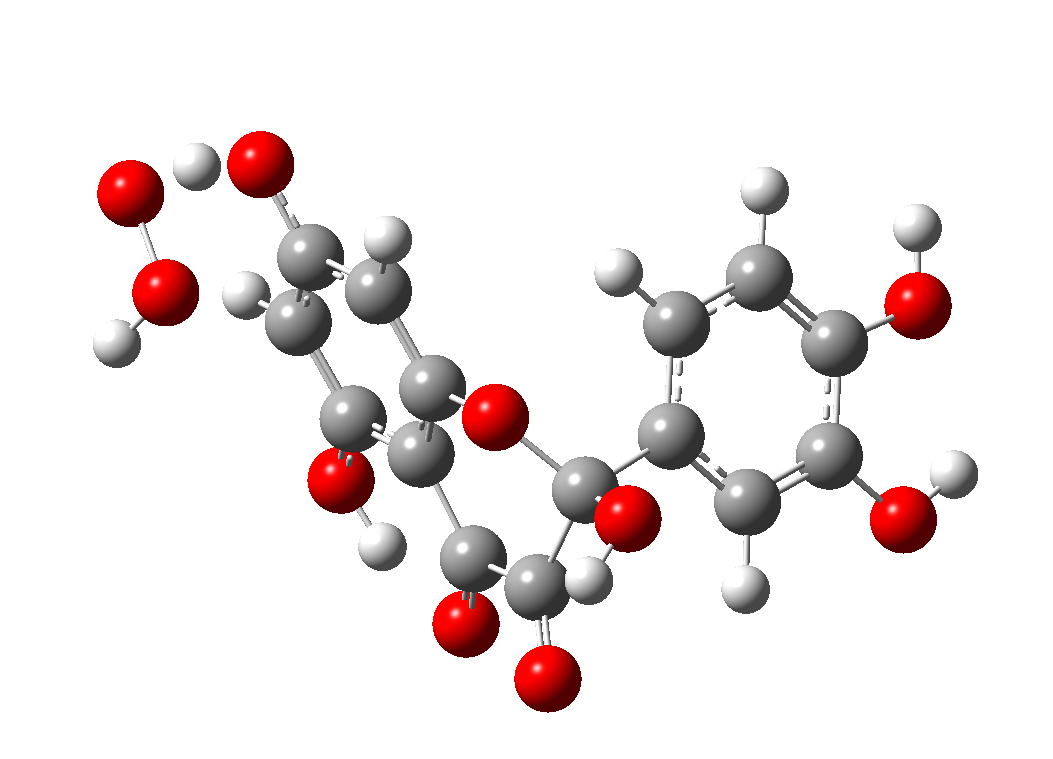 | 6 -1.213809000 0.129597000 -0.494947000  6 -1.005851000 0.722848000 0.764108000  6 -2.178467000 -0.841822000 -0.663372000  6 -1.793344000 0.302499000 1.880649000  6 -2.957049000 -1.244774000 0.454680000  6 -2.760995000 -0.660736000 1.724012000  6 0.821377000 1.043517000 -1.401894000  6 0.658693000 2.189908000 -0.401201000  8 -0.503970000 0.492868000 -1.595189000  6 -0.047273000 1.799480000 0.898280000  6 1.766658000 0.000385000 -0.840863000  6 1.492153000 -1.355972000 -0.944133000  6 2.956057000 0.439311000 -0.257146000  6 2.410709000 -2.281879000 -0.460294000  6 3.867088000 -0.481218000 0.226173000  6 3.587944000 -1.847857000 0.123216000  8 -1.610670000 0.850030000 3.085496000  8 -3.851485000 -2.177226000 0.302409000  8 0.181975000 2.415206000 1.924439000  8 1.021179000 3.307956000 -0.621717000  8 1.271730000 1.490376000 -2.612579000  8 4.545791000 -2.672200000 0.630584000  8 5.021416000 -0.058204000 0.793927000  1 -2.331454000 -1.306978000 -1.625912000  1 -3.363618000 -0.980717000 2.561340000  1 0.581438000 -1.703596000 -1.408342000  1 3.200496000 1.491787000 -0.183840000  1 2.211191000 -3.343515000 -0.537909000  1 -0.920917000 1.536356000 3.025540000  1 -4.709877000 -1.798734000 -0.444705000  1 0.965398000 2.395687000 -2.757127000  1 4.296400000 -3.596174000 0.525573000  1 5.532559000 -0.827374000 1.072577000  8 -5.497193000 -1.147240000 -1.010399000  8 -4.816631000 -0.001896000 -1.295579000  1 -5.182108000 0.656157000 -0.681954000 |
| --- | --- |

Optimized geometry and Cartesian coordinates of 2,5,7,3′,4′-pentahydroxy-3,4-flavandione (**3**)

3’-OH∙∙∙^•^OOH TS at SMD/um052x/6-311++g(d,p) level of theory in pentyl ethanoate

| 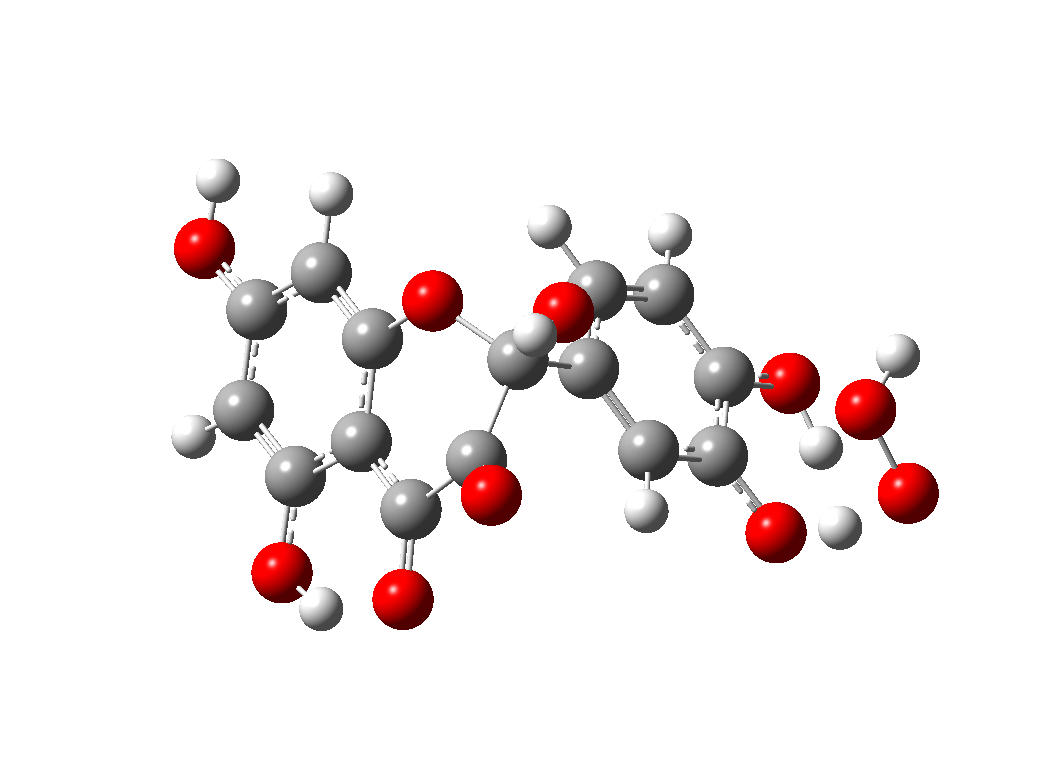 | 6 -2.105857000 0.049233000 -0.865401000  6 -2.144298000 0.572714000 0.445207000  6 -2.936124000 -0.976974000 -1.250963000  6 -3.062480000 0.012080000 1.372515000  6 -3.832738000 -1.497395000 -0.308881000  6 -3.899824000 -1.021636000 0.995846000  6 -0.022382000 1.136768000 -1.344627000  6 -0.422117000 2.203119000 -0.319221000  8 -1.268450000 0.561243000 -1.809393000  6 -1.323840000 1.693361000 0.806643000  6 0.849349000 0.065911000 -0.721653000  6 0.788721000 -1.248974000 -1.211399000  6 1.750721000 0.396402000 0.267927000  6 1.619068000 -2.235589000 -0.711683000  6 2.601982000 -0.590539000 0.792808000  6 2.523732000 -1.915586000 0.293001000  8 -3.138085000 0.478478000 2.619700000  8 -4.671034000 -2.496528000 -0.632926000  8 -1.321084000 2.260104000 1.890216000  8 -0.097867000 3.349504000 -0.425144000  8 0.612538000 1.684442000 -2.424615000  8 3.336530000 -2.848111000 0.802413000  8 3.469156000 -0.357868000 1.762645000  1 -2.889359000 -1.362080000 -2.260255000  1 -4.600221000 -1.445752000 1.699778000  1 0.087217000 -1.492261000 -1.996689000  1 1.848296000 1.402893000 0.657287000  1 1.575089000 -3.248467000 -1.085911000  1 -2.515629000 1.225670000 2.713513000  1 -4.556627000 -2.748805000 -1.555996000  1 0.324459000 2.600696000 -2.535384000  1 3.838075000 -2.440858000 1.525286000  1 4.377843000 0.125770000 1.331594000  8 5.307229000 0.452868000 0.529291000  8 4.671650000 0.406397000 -0.666689000  1 5.002719000 -0.398390000 -1.097916000 |
| --- | --- |

Optimized geometry and Cartesian coordinates of 2,5,7,3′,4′-pentahydroxy-3,4-flavandione (**3**)

4’-OH∙∙∙^•^OOH TS at SMD/um052x/6-311++g(d,p) level of theory in pentyl ethanoate

| 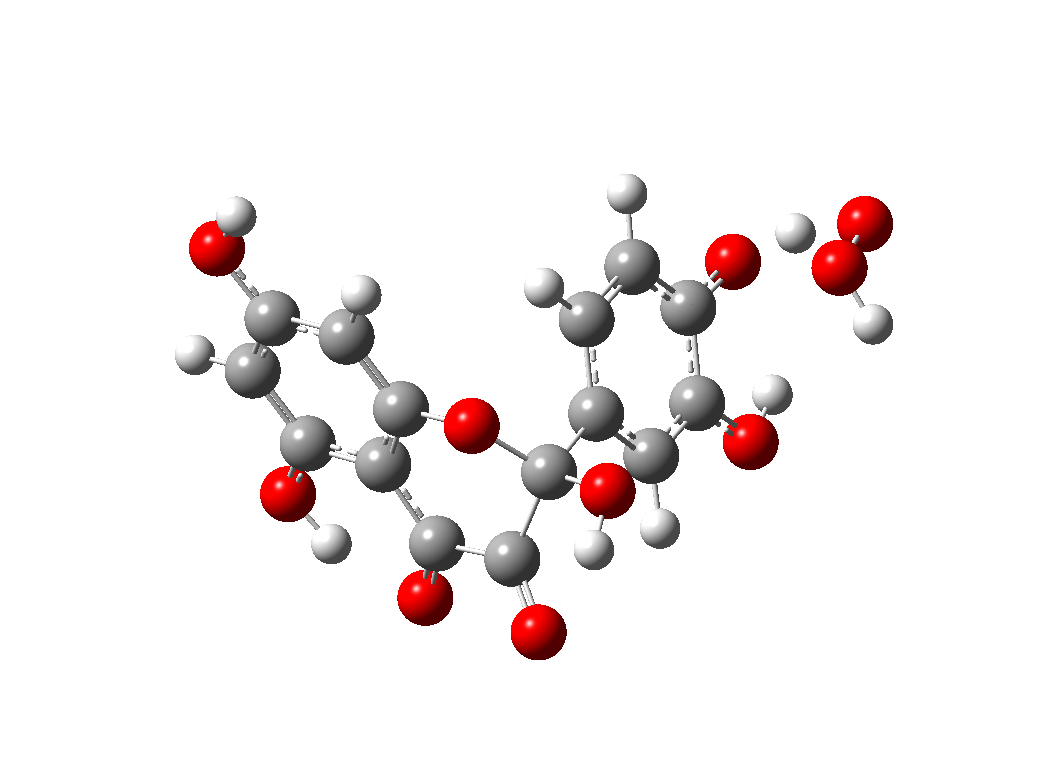 | 6 -2.059159000 -0.267314000 -0.901379000  6 -2.285496000 0.352049000 0.348039000  6 -2.648967000 -1.467370000 -1.221210000  6 -3.134095000 -0.299323000 1.283075000  6 -3.483559000 -2.073735000 -0.273792000  6 -3.727171000 -1.508801000 0.973364000  6 -0.223082000 1.192927000 -1.397275000  6 -0.911388000 2.237004000 -0.509825000  8 -1.269433000 0.307859000 -1.851401000  6 -1.738952000 1.647943000 0.630923000  6 0.827560000 0.427422000 -0.612126000  6 0.995867000 -0.950997000 -0.810086000  6 1.640697000 1.137295000 0.260185000  6 1.986799000 -1.622309000 -0.130834000  6 2.635632000 0.462442000 0.953258000  6 2.822526000 -0.931099000 0.762292000  8 -3.380380000 0.251486000 2.472339000  8 -4.083659000 -3.246868000 -0.535786000  8 -1.912373000 2.293186000 1.655239000  8 -0.838635000 3.411017000 -0.728154000  8 0.354401000 1.768864000 -2.494617000  8 3.775587000 -1.514927000 1.467196000  8 3.434507000 1.117551000 1.804171000  1 -2.463255000 -1.924056000 -2.183733000  1 -4.374752000 -2.000410000 1.683909000  1 0.360564000 -1.479646000 -1.504393000  1 1.530839000 2.203551000 0.412519000  1 2.150937000 -2.681839000 -0.272121000  1 -2.922635000 1.113375000 2.520145000  1 -3.854843000 -3.554141000 -1.420198000  1 -0.100417000 2.598642000 -2.694913000  1 4.669930000 -1.654092000 0.814542000  1 4.007702000 0.465162000 2.234479000  8 4.833370000 -0.823392000 -1.042754000  1 5.122853000 0.102808000 -0.996517000  8 5.547838000 -1.465547000 -0.086404000 |
| --- | --- |

Optimized geometry and Cartesian coordinates of 2,5,7,3′,4′-pentahydroxy-3,4-flavandione (**3**)

5-OH∙∙∙^•^OOCH_3_ TS at SMD/um052x/6-311++g(d,p) level of theory in pentyl ethanoate

| 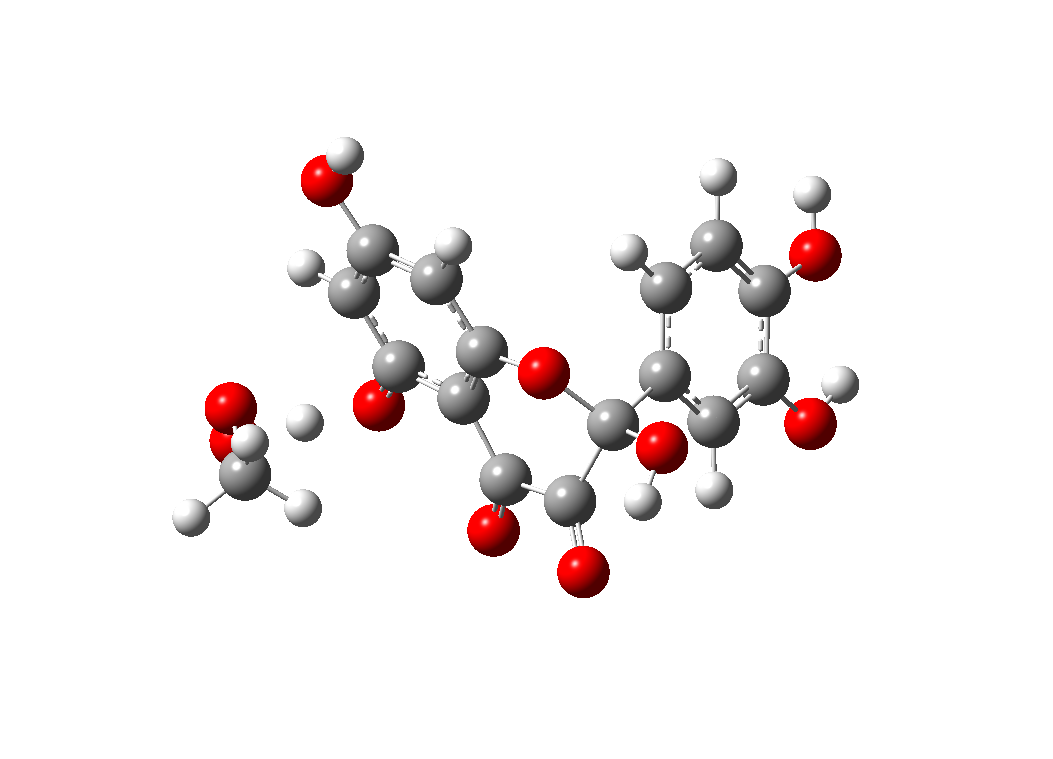 | 6 -0.785551000 0.316155000 1.170820000  6 -1.011340000 -0.226356000 -0.097272000  6 -1.469014000 1.447826000 1.634646000  6 -1.923335000 0.463670000 -0.964186000  6 -2.371706000 2.080997000 0.801831000  6 -2.592172000 1.613853000 -0.497533000  6 1.178797000 -1.014398000 1.540075000  6 0.494138000 -2.069230000 0.672397000  8 0.088959000 -0.209530000 2.061710000  6 -0.398666000 -1.506031000 -0.446238000  6 2.124309000 -0.163287000 0.719520000  6 2.222167000 1.206148000 0.921000000  6 2.933654000 -0.798951000 -0.221206000  6 3.133371000 1.947905000 0.176012000  6 3.836515000 -0.060664000 -0.964923000  6 3.932029000 1.319562000 -0.763175000  8 -2.167843000 0.037618000 -2.160335000  8 -3.056782000 3.177180000 1.175977000  8 -0.552896000 -2.162588000 -1.445647000  8 0.609394000 -3.241962000 0.885664000  8 1.829695000 -1.576665000 2.602487000  8 4.847630000 1.956966000 -1.546302000  8 4.620375000 -0.675812000 -1.881571000  1 -1.260824000 1.812682000 2.632011000  1 -3.263746000 2.140995000 -1.158531000  1 1.605392000 1.701271000 1.656419000  1 2.886524000 -1.868397000 -0.387480000  1 3.224324000 3.016504000 0.327076000  1 -3.349055000 -0.161924000 -2.264852000  1 -2.825442000 3.430500000 2.076943000  1 1.449180000 -2.449128000 2.775611000  1 4.847124000 2.904343000 -1.375024000  1 5.173740000 -0.014431000 -2.313195000  8 -4.645403000 -0.087430000 -0.760850000  8 -4.481226000 -0.418521000 -2.066694000  6 -4.564721000 -1.270222000 0.047847000  1 -3.659638000 -1.822464000 -0.199270000  1 -4.549175000 -0.921487000 1.077552000  1 -5.444480000 -1.884383000 -0.140476000 |
| --- | --- |

Optimized geometry and Cartesian coordinates of 2,5,7,3′,4′-pentahydroxy-3,4-flavandione (**3**)

7-OH∙∙∙^•^OOCH_3_ TS at SMD/um052x/6-311++g(d,p) level of theory in pentyl ethanoate

| 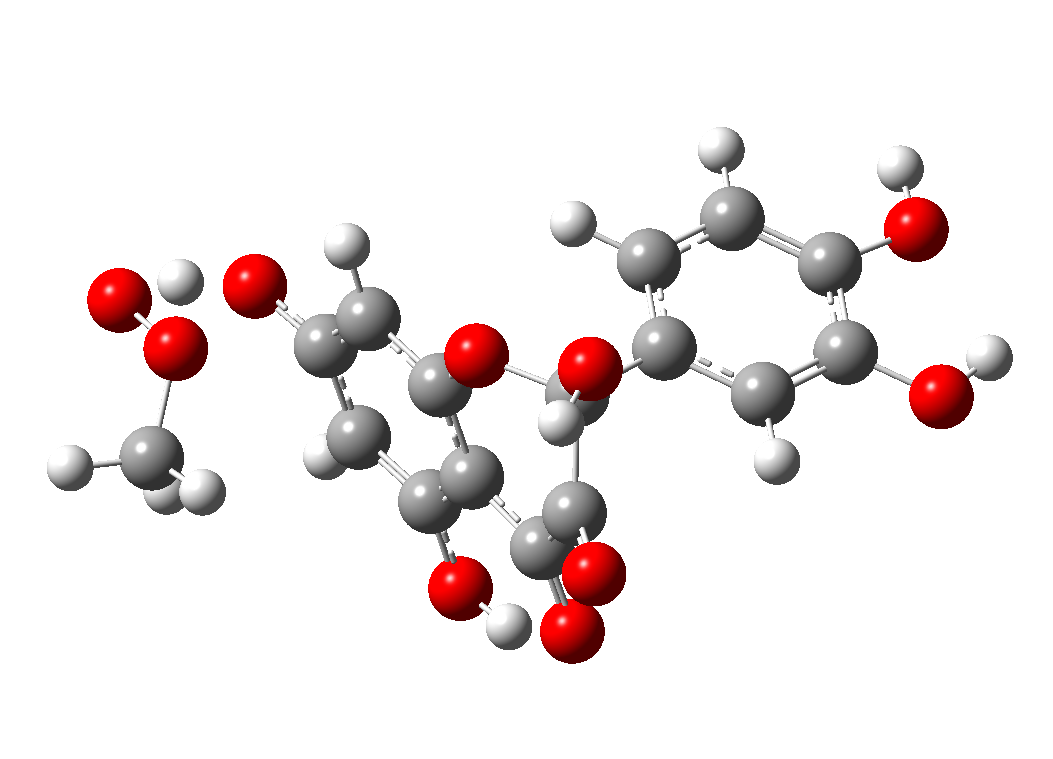 | 6 -1.051867000 0.020230000 -0.393792000  6 -0.847676000 0.547837000 0.886905000  6 -1.979395000 -0.991638000 -0.600937000  6 -1.589627000 0.008271000 1.994287000  6 -2.733246000 -1.488315000 0.502058000  6 -2.514060000 -0.984727000 1.803034000  6 0.911579000 1.082206000 -1.294109000  6 0.720364000 2.153786000 -0.217511000  8 -0.381311000 0.459272000 -1.486852000  6 0.065774000 1.651562000 1.070706000  6 1.936909000 0.069027000 -0.824913000  6 1.716761000 -1.296537000 -0.928822000  6 3.145769000 0.555516000 -0.324719000  6 2.709358000 -2.185403000 -0.528226000  6 4.130613000 -0.328028000 0.075128000  6 3.905782000 -1.705205000 -0.026452000  8 -1.393843000 0.493575000 3.222655000  8 -3.641803000 -2.381360000 0.279936000  8 0.294497000 2.212644000 2.129259000  8 1.032010000 3.297908000 -0.374859000  8 1.299070000 1.626142000 -2.486610000  8 4.935050000 -2.490404000 0.396698000  8 5.304700000 0.140926000 0.560213000  1 -2.101645000 -1.427567000 -1.580658000  1 -3.087807000 -1.371924000 2.631943000  1 0.791985000 -1.681287000 -1.331831000  1 3.344961000 1.617725000 -0.255126000  1 2.550578000 -3.253592000 -0.608470000  1 -0.737547000 1.214447000 3.182019000  1 -4.456039000 -1.904016000 -0.533819000  1 0.956192000 2.526924000 -2.558404000  1 4.720385000 -3.423611000 0.296446000  1 5.871416000 -0.606558000 0.784902000  8 -5.128021000 -1.209252000 -1.140296000  8 -4.325231000 -0.141435000 -1.393842000  6 -4.744444000 0.975870000 -0.596250000  1 -4.738236000 0.700696000 0.456975000  1 -4.026586000 1.765632000 -0.803166000  1 -5.744170000 1.272303000 -0.909572000 |
| --- | --- |

Optimized geometry and Cartesian coordinates of 2,5,7,3′,4′-pentahydroxy-3,4-flavandione (**3**)

3’-OH∙∙∙^•^OOCH_3_ TS at SMD/um052x/6-311++g(d,p) level of theory in pentyl ethanoate

| 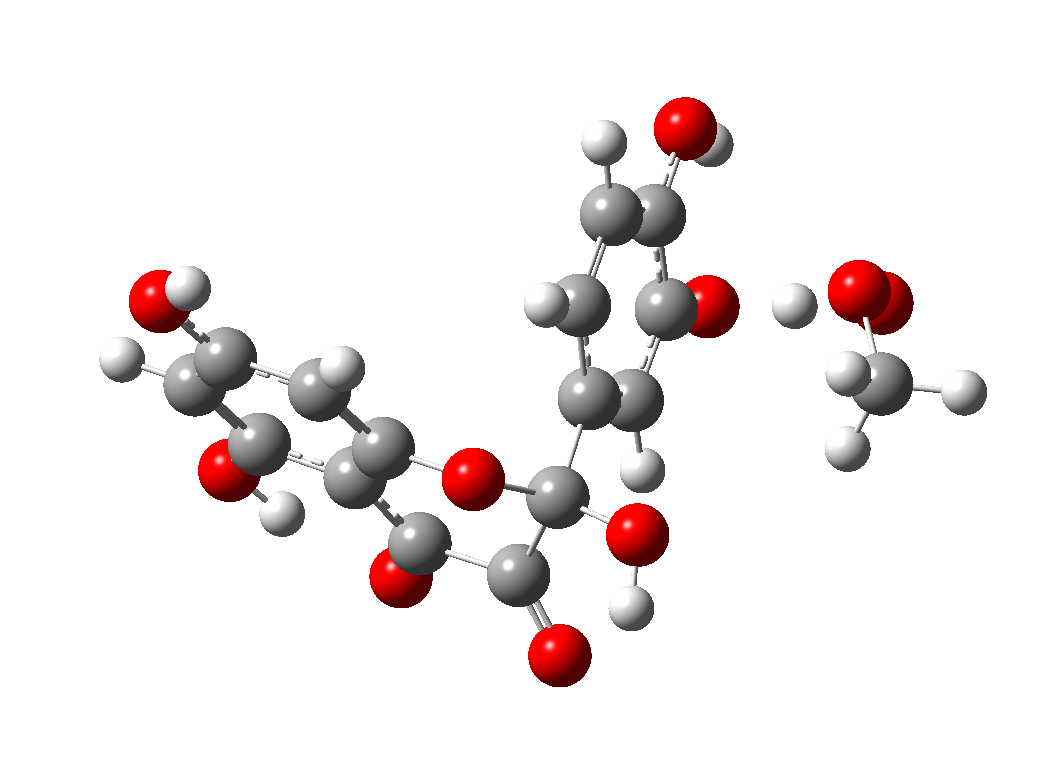 | 6 -2.266006000 0.101554000 -0.898121000  6 -2.370193000 0.520324000 0.446418000  6 -3.064614000 -0.899527000 -1.399039000  6 -3.315785000 -0.124614000 1.286832000  6 -3.989295000 -1.506173000 -0.538714000  6 -4.118650000 -1.137206000 0.795456000  6 -0.180949000 1.247926000 -1.190620000  6 -0.645280000 2.229355000 -0.109745000  8 -1.392536000 0.694337000 -1.759362000  6 -1.583340000 1.617617000 0.931457000  6 0.677219000 0.146807000 -0.602405000  6 0.666957000 -1.131730000 -1.189768000  6 1.514967000 0.416675000 0.457558000  6 1.482772000 -2.137013000 -0.711984000  6 2.362547000 -0.587281000 0.957586000  6 2.327667000 -1.877705000 0.364297000  8 -3.450653000 0.242235000 2.561679000  8 -4.794069000 -2.489532000 -0.975635000  8 -1.633400000 2.092105000 2.057595000  8 -0.336958000 3.385236000 -0.112684000  8 0.499681000 1.881874000 -2.193930000  8 3.115634000 -2.839004000 0.846327000  8 3.183125000 -0.384720000 1.968603000  1 -2.973100000 -1.203250000 -2.433211000  1 -4.839433000 -1.625402000 1.434214000  1 0.014430000 -1.327220000 -2.028959000  1 1.567916000 1.391261000 0.928010000  1 1.480869000 -3.121650000 -1.157279000  1 -2.840314000 0.984489000 2.740127000  1 -4.637120000 -2.664427000 -1.910419000  1 0.180787000 2.791331000 -2.272433000  1 3.600103000 -2.482340000 1.605651000  1 4.217380000 -0.161521000 1.534344000  8 4.599115000 -0.340864000 -0.426626000  8 5.171819000 -0.003336000 0.751107000  6 4.409405000 0.826763000 -1.240731000  1 3.784884000 0.506204000 -2.070898000  1 3.923472000 1.606985000 -0.659411000  1 5.381541000 1.164256000 -1.597190000 |
| --- | --- |

Optimized geometry and Cartesian coordinates of 2,5,7,3′,4′-pentahydroxy-3,4-flavandione (**3**)

4’-OH∙∙∙^•^OOCH_3_ TS at SMD/um052x/6-311++g(d,p) level of theory in pentyl ethanoate

| 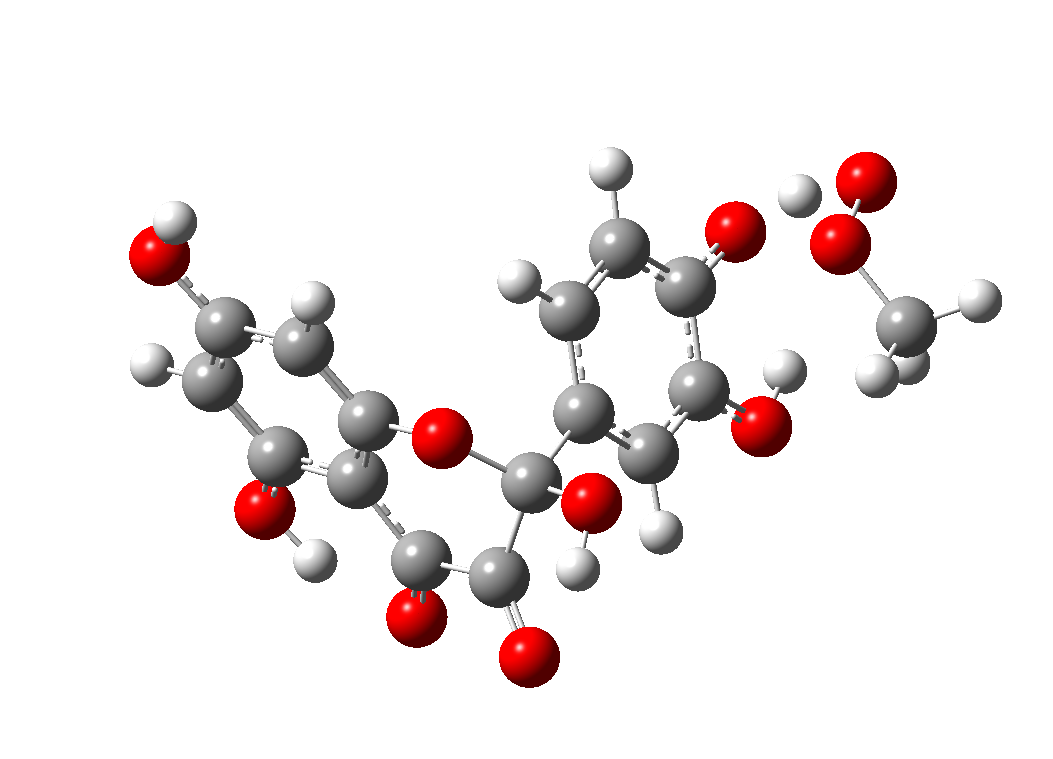 | 6 -2.257824000 -0.216920000 -0.934797000  6 -2.499618000 0.369200000 0.327721000  6 -2.868081000 -1.391672000 -1.305857000  6 -3.384615000 -0.290061000 1.222455000  6 -3.741416000 -2.004812000 -0.398510000  6 -4.000215000 -1.473529000 0.860404000  6 -0.377960000 1.215125000 -1.339241000  6 -1.071000000 2.249121000 -0.444442000  8 -1.431579000 0.367612000 -1.847359000  6 -1.930684000 1.642833000 0.662709000  6 0.628154000 0.403804000 -0.543663000  6 0.787946000 -0.967670000 -0.794329000  6 1.407287000 1.062807000 0.397591000  6 1.735958000 -1.683969000 -0.100346000  6 2.358081000 0.341769000 1.104602000  6 2.541127000 -1.045835000 0.860829000  8 -3.644125000 0.228985000 2.423237000  8 -4.366428000 -3.152047000 -0.712298000  8 -2.108520000 2.258290000 1.704457000  8 -0.980195000 3.426738000 -0.634752000  8 0.244059000 1.806741000 -2.403340000  8 3.457742000 -1.665897000 1.572752000  8 3.126956000 0.940569000 2.021752000  1 -2.668480000 -1.823557000 -2.276962000  1 -4.676768000 -1.970501000 1.539614000  1 0.178335000 -1.454691000 -1.540613000  1 1.305333000 2.123275000 0.591548000  1 1.892981000 -2.738190000 -0.283193000  1 -3.163897000 1.075581000 2.509920000  1 -4.123846000 -3.438012000 -1.600224000  1 -0.193580000 2.646512000 -2.600350000  1 4.368873000 -1.839188000 0.886168000  1 3.670769000 0.252532000 2.436694000  8 4.577995000 -0.881987000 -0.861966000  8 5.217254000 -1.695344000 0.008891000  6 5.146604000 0.432740000 -0.810030000  1 4.526353000 1.043743000 -1.461048000  1 5.125055000 0.805373000 0.213130000  1 6.171121000 0.390243000 -1.176242000 |
| --- | --- |

Optimized geometry and Cartesian coordinates of 2,5,7,3′,4′-pentahydroxy-3,4-flavandione (**3**)

3’,4’-diOH∙∙∙O_2_^•−^ TS at SMD/um052x/6-311++g(d,p) level of theory in pentyl ethanoate

| 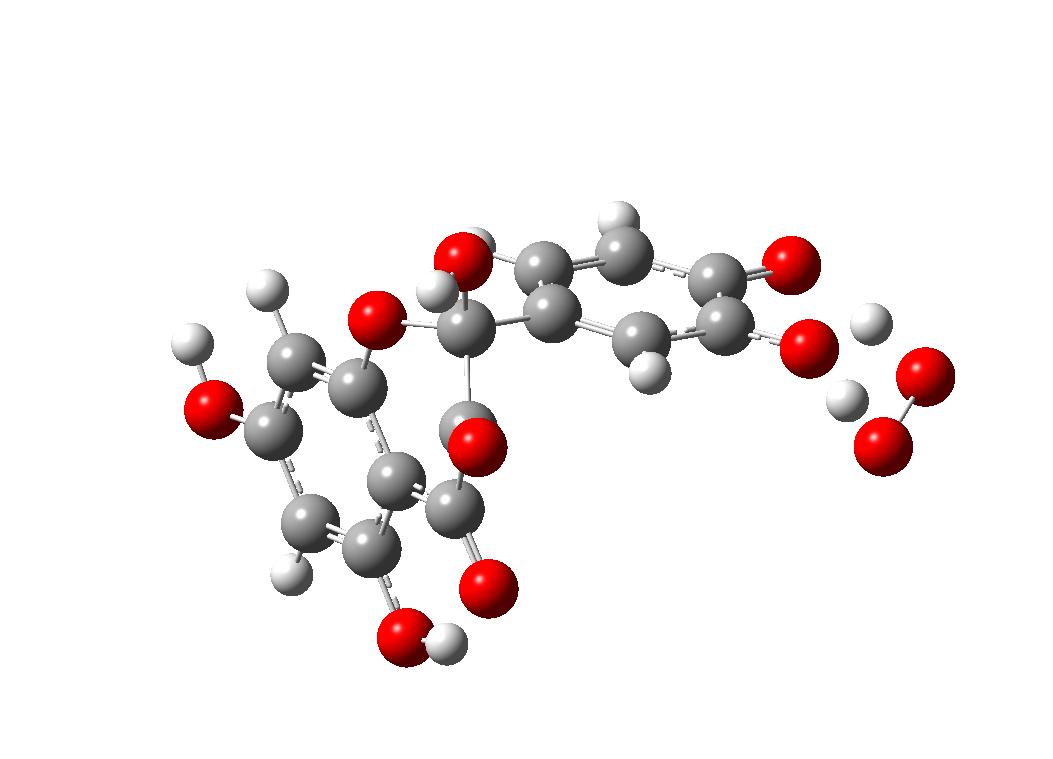 | 6 -2.277260000 0.164049000 -0.652945000  6 -2.053432000 0.095943000 0.738850000  6 -3.086932000 -0.752207000 -1.289341000  6 -2.666130000 -0.951557000 1.475736000  6 -3.676881000 -1.764361000 -0.526681000  6 -3.473174000 -1.879686000 0.844301000  6 -0.517290000 1.779543000 -0.953524000  6 -0.786493000 2.223462000 0.484973000  8 -1.734403000 1.142702000 -1.419124000  6 -1.287945000 1.112347000 1.404955000  8 -2.483189000 -1.048257000 2.794287000  8 -4.473566000 -2.685864000 -1.101109000  8 -1.078491000 1.171582000 2.608877000  8 -0.649037000 3.355512000 0.853308000  8 -0.280758000 2.856265000 -1.769418000  1 -3.243030000 -0.685113000 -2.357434000  1 -3.938515000 -2.676540000 1.404906000  1 -1.912117000 -0.307974000 3.082544000  1 -4.542528000 -2.523803000 -2.048416000  1 -0.681160000 3.642402000 -1.375345000  6 0.644520000 0.811497000 -0.967283000  6 1.789791000 1.149849000 -0.267398000  6 0.597136000 -0.392743000 -1.691890000  6 2.929953000 0.328290000 -0.275641000  1 1.857855000 2.079318000 0.286323000  6 1.689969000 -1.225986000 -1.710257000  1 -0.289846000 -0.658415000 -2.249154000  6 2.891607000 -0.918190000 -1.015699000  8 4.022928000 0.698811000 0.347101000  1 1.670238000 -2.150426000 -2.272539000  8 3.905437000 -1.703268000 -1.075318000  1 4.322500000 -0.170449000 1.097758000  8 4.609390000 -1.129995000 1.749778000  8 5.367921000 -1.839355000 0.869450000  1 4.802101000 -1.775559000 -0.020239000 |
| --- | --- |

Optimized geometry and Cartesian coordinates of 2-(3,4-dihydroxybenzoyl)-2,4,6-trihydroxy-3(2*H*)-benzofuranone (**4**) 5-OH∙∙∙^•^OOH TS at SMD/um052x/6-311++g(d,p) level of theory in pentyl ethanoate

| 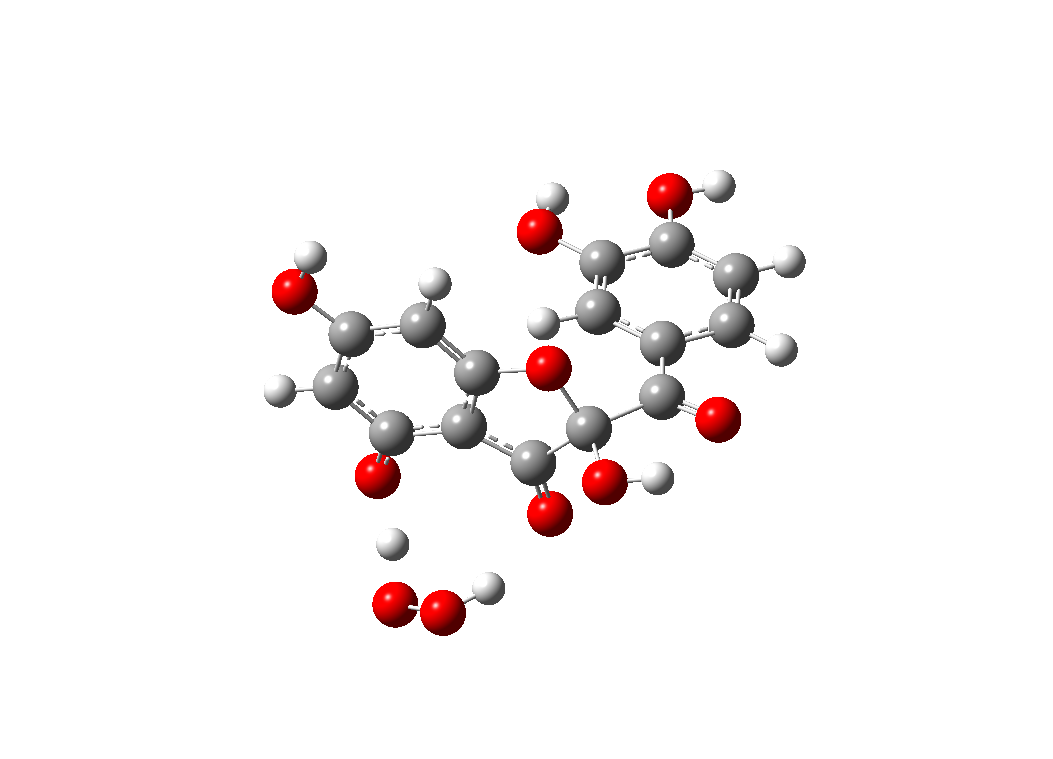 | 6 -1.278801000 0.416003000 1.322981000  6 -1.587274000 0.023006000 0.024040000  6 -1.792692000 1.558116000 1.915398000  6 -2.444616000 0.839697000 -0.778016000  6 -2.671156000 2.325579000 1.140979000  6 -3.004023000 1.980387000 -0.167834000  8 -0.388004000 -0.391064000 1.946400000  6 -0.139524000 -1.527620000 1.093026000  6 -0.848619000 -1.180194000 -0.240131000  6 1.396480000 -1.759188000 1.029165000  6 2.305150000 -0.785164000 0.410227000  6 4.125125000 0.945269000 -0.765933000  6 4.550955000 -0.297552000 -0.318269000  6 2.787536000 1.333245000 -0.626544000  6 3.643799000 -1.159875000 0.271495000  6 1.882889000 0.469843000 -0.043978000  8 1.779697000 -2.797494000 1.522491000  8 -0.801657000 -1.909572000 -1.211242000  8 4.931079000 1.861390000 -1.355206000  8 -0.748475000 -2.643813000 1.591788000  8 -3.234833000 3.444376000 1.628549000  8 -2.655267000 0.614400000 -2.028021000  8 2.377832000 2.551026000 -1.055808000  1 -1.525122000 1.835994000 2.926249000  1 -3.667705000 2.611489000 -0.740599000  1 5.588647000 -0.583723000 -0.435510000  1 3.963212000 -2.130737000 0.619716000  1 0.864952000 0.814904000 0.047695000  1 5.834824000 1.535061000 -1.428283000  1 -0.045724000 -3.205532000 1.960918000  1 -2.954379000 3.600403000 2.537546000  1 -3.085538000 -0.507734000 -2.413720000  1 3.128250000 3.019867000 -1.439267000  8 -3.677992000 -1.416743000 -2.641212000  8 -3.476247000 -2.275965000 -1.607447000  1 -2.501850000 -2.371635000 -1.556718000 |
| --- | --- |

Optimized geometry and Cartesian coordinates of 2-(3,4-dihydroxybenzoyl)-2,4,6-trihydroxy-3(2*H*)-benzofuranone (**4**) 7-OH∙∙∙^•^OOH TS at SMD/um052x/6-311++g(d,p) level of theory in pentyl ethanoate

| 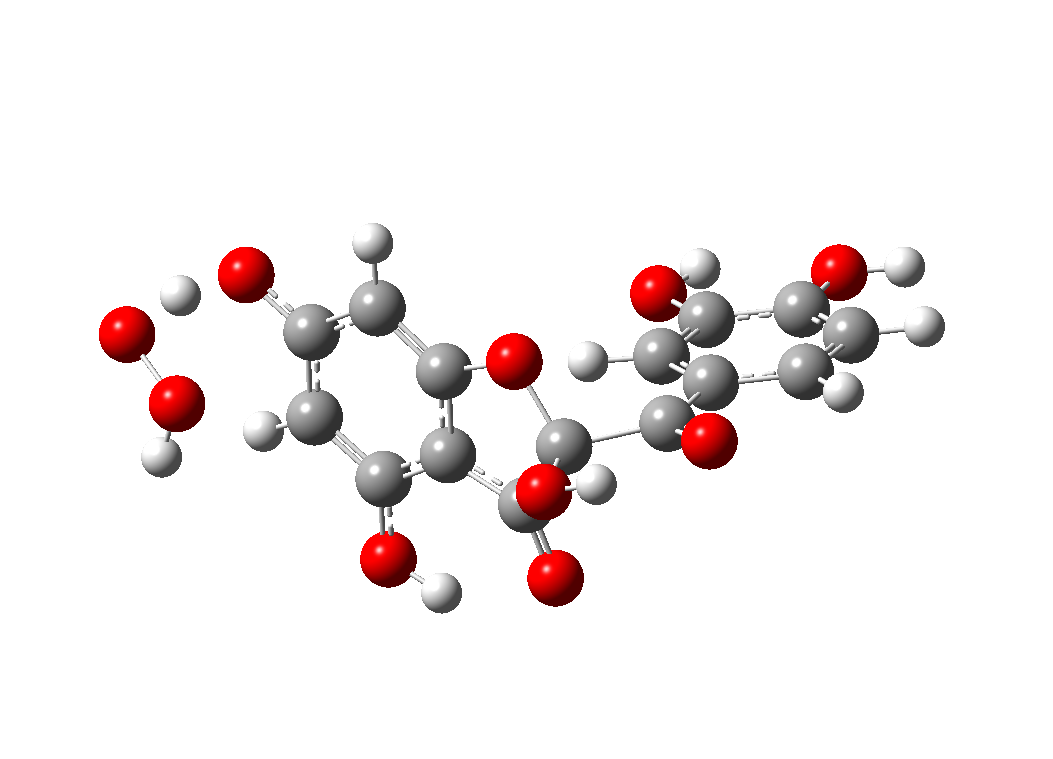 | 6 -1.268153000 0.323830000 -0.670876000  6 -1.234046000 0.368044000 0.730809000  6 -2.137027000 -0.482352000 -1.350036000  6 -2.088578000 -0.402085000 1.519899000  6 -3.011760000 -1.270550000 -0.552122000  6 -2.990050000 -1.229037000 0.870637000  8 -0.334734000 1.139666000 -1.234907000  6 0.282475000 1.936876000 -0.199924000  6 -0.205978000 1.298880000 1.125282000  6 1.813667000 1.935408000 -0.456390000  6 2.613841000 0.708270000 -0.352863000  6 4.248272000 -1.521179000 -0.141627000  6 4.813294000 -0.277038000 -0.383185000  6 2.861893000 -1.660966000 -0.007354000  6 3.997752000 0.835578000 -0.490301000  6 2.050296000 -0.550081000 -0.112538000  8 2.289003000 3.011272000 -0.746777000  8 0.174530000 1.593458000 2.234105000  8 4.956582000 -2.670208000 -0.020850000  8 -0.186551000 3.220012000 -0.228720000  8 -3.865209000 -2.066558000 -1.136217000  8 -2.040380000 -0.348407000 2.857819000  8 2.313336000 -2.878445000 0.219524000  1 -2.185974000 -0.535216000 -2.426917000  1 -3.667096000 -1.864083000 1.425031000  1 5.887396000 -0.186335000 -0.484508000  1 4.425855000 1.809828000 -0.674057000  1 0.986653000 -0.709402000 -0.021004000  1 5.900792000 -2.510633000 -0.128288000  1 0.518092000 3.767484000 -0.615067000  1 -4.909567000 -1.503669000 -1.209202000  1 -1.342387000 0.263020000 3.134860000  1 3.013617000 -3.540688000 0.250169000  8 -5.214098000 0.325823000 -0.476221000  1 -5.439226000 0.236951000 0.464489000  8 -5.800421000 -0.740218000 -1.079251000 |
| --- | --- |

Optimized geometry and Cartesian coordinates of 2-(3,4-dihydroxybenzoyl)-2,4,6-trihydroxy-3(2*H*)-benzofuranone (**4**) 3’-OH∙∙∙^•^OOH TS at SMD/um052x/6-311++g(d,p) level of theory in pentyl ethanoate

| 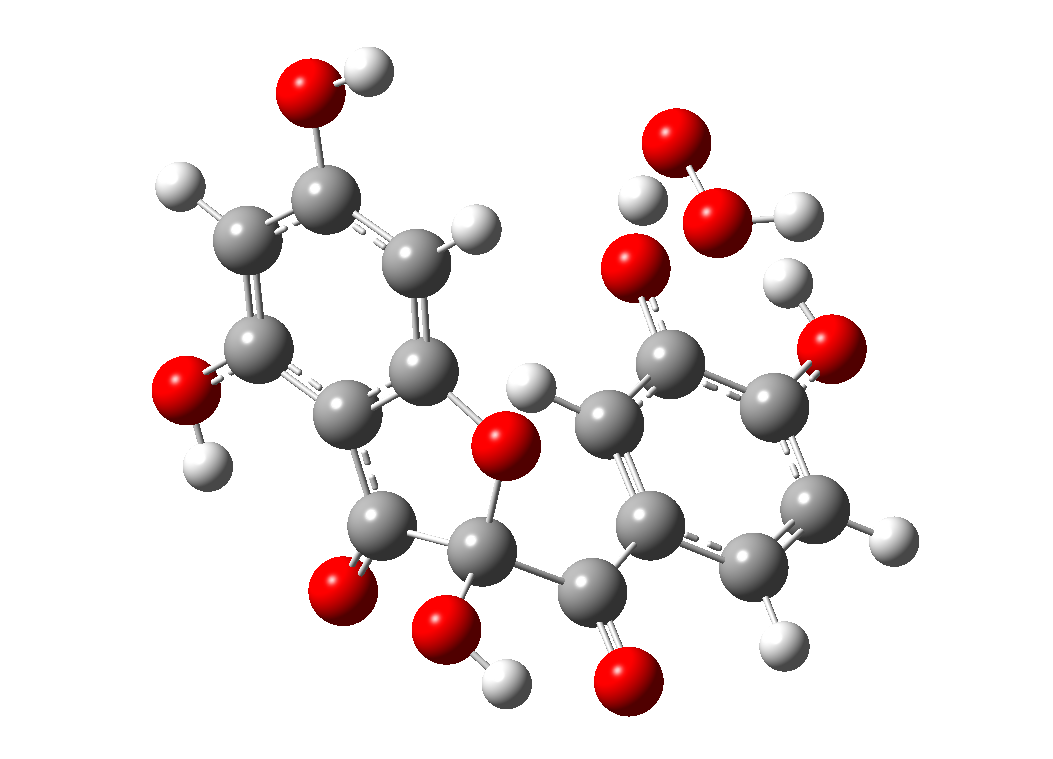 | 6 -1.824814000 0.124740000 -0.845857000  6 -2.101309000 0.522069000 0.462632000  6 -2.355732000 -1.016347000 -1.395386000  6 -2.951612000 -0.234458000 1.278310000  6 -3.212590000 -1.750813000 -0.559300000  6 -3.520613000 -1.380300000 0.755229000  8 -0.951924000 0.959948000 -1.473086000  6 -0.674464000 2.077103000 -0.601511000  6 -1.340186000 1.697082000 0.751262000  6 0.861981000 2.283228000 -0.584514000  6 1.781770000 1.224368000 -0.135414000  6 3.673210000 -0.686878000 0.621748000  6 4.103476000 0.544933000 0.139773000  6 2.285646000 -0.968637000 0.735678000  6 3.164723000 1.483955000 -0.233497000  6 1.350114000 0.001633000 0.350967000  8 1.254369000 3.353362000 -0.994018000  8 -1.235229000 2.329705000 1.779877000  8 4.550779000 -1.620269000 0.985928000  8 -1.284445000 3.212514000 -1.057675000  8 -3.793837000 -2.881333000 -1.000333000  8 -3.204091000 0.142959000 2.539117000  8 1.943390000 -2.147600000 1.219974000  1 -2.129158000 -1.333341000 -2.403723000  1 -4.184214000 -1.995241000 1.344991000  1 5.162298000 0.746891000 0.064795000  1 3.485620000 2.443667000 -0.612236000  1 0.308791000 -0.264296000 0.450760000  1 4.054567000 -2.380143000 1.328584000  1 -0.585974000 3.792763000 -1.402648000  1 -3.517574000 -3.071128000 -1.903856000  1 -2.711592000 0.952438000 2.738414000  1 1.517501000 -2.777258000 0.392885000  8 1.522256000 -2.225781000 -1.566566000  1 2.383337000 -2.409685000 -1.977043000  8 1.296082000 -3.277847000 -0.740922000 |
| --- | --- |

Optimized geometry and Cartesian coordinates of 2-(3,4-dihydroxybenzoyl)-2,4,6-trihydroxy-3(2*H*)-benzofuranone (**4**) 4’-OH∙∙∙^•^OOH TS at SMD/um052x/6-311++g(d,p) level of theory in pentyl ethanoate

| 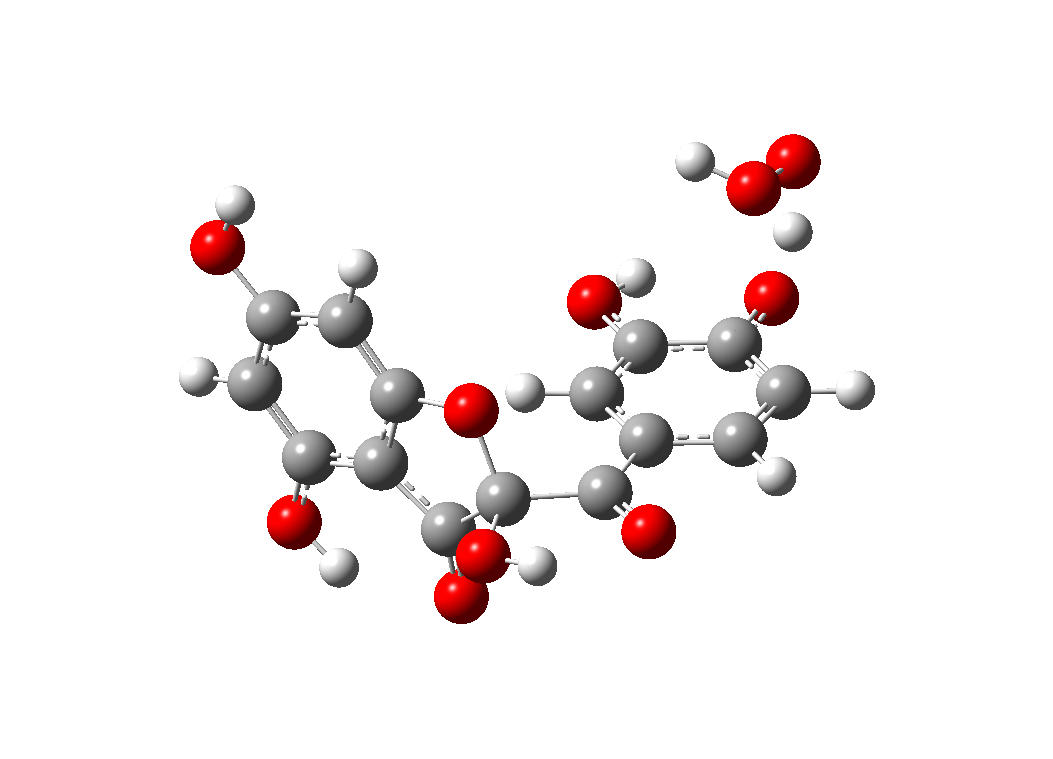 | 6 -2.117147000 -0.130568000 -0.939483000  6 -2.433442000 0.152816000 0.389048000  6 -2.559582000 -1.264056000 -1.575780000  6 -3.225164000 -0.724603000 1.139856000  6 -3.361845000 -2.120679000 -0.803846000  6 -3.698585000 -1.872485000 0.531737000  8 -1.304395000 0.809628000 -1.496523000  6 -1.160754000 1.901862000 -0.561116000  6 -1.788538000 1.374565000 0.760042000  6 0.341080000 2.280242000 -0.528265000  6 1.380735000 1.339548000 -0.053463000  6 3.455505000 -0.307173000 0.804481000  6 3.735849000 0.993306000 0.344236000  6 2.109330000 -0.758368000 0.841220000  6 2.712963000 1.803921000 -0.075785000  6 1.079058000 0.062056000 0.404607000  8 0.616033000 3.384667000 -0.936363000  8 -1.751011000 1.949633000 1.825665000  8 4.380694000 -1.139457000 1.234300000  8 -1.900114000 2.981217000 -0.956809000  8 -3.854902000 -3.255792000 -1.332860000  8 -3.515694000 -0.458311000 2.420463000  8 1.845235000 -1.990397000 1.292440000  1 -2.316051000 -1.482351000 -2.606044000  1 -4.316052000 -2.575478000 1.070538000  1 4.765257000 1.323651000 0.329402000  1 2.912002000 2.804087000 -0.430166000  1 0.075963000 -0.333650000 0.442192000  1 4.865104000 -1.629236000 0.343889000  1 -1.286060000 3.632932000 -1.331884000  1 -3.579307000 -3.347280000 -2.251705000  1 -3.094657000 0.373677000 2.680826000  1 2.673561000 -2.373899000 1.619038000  8 4.102403000 -1.469890000 -1.532710000  1 3.390779000 -2.110515000 -1.698402000  8 5.018336000 -2.144329000 -0.796681000 |
| --- | --- |

Optimized geometry and Cartesian coordinates of 2-(3,4-dihydroxybenzoyl)-2,4,6-trihydroxy-3(2*H*)-benzofuranone (**4**) 5-OH∙∙∙^•^OOCH_3_ TS at SMD/um052x/6-311++g(d,p) level of theory in pentyl ethanoate

| 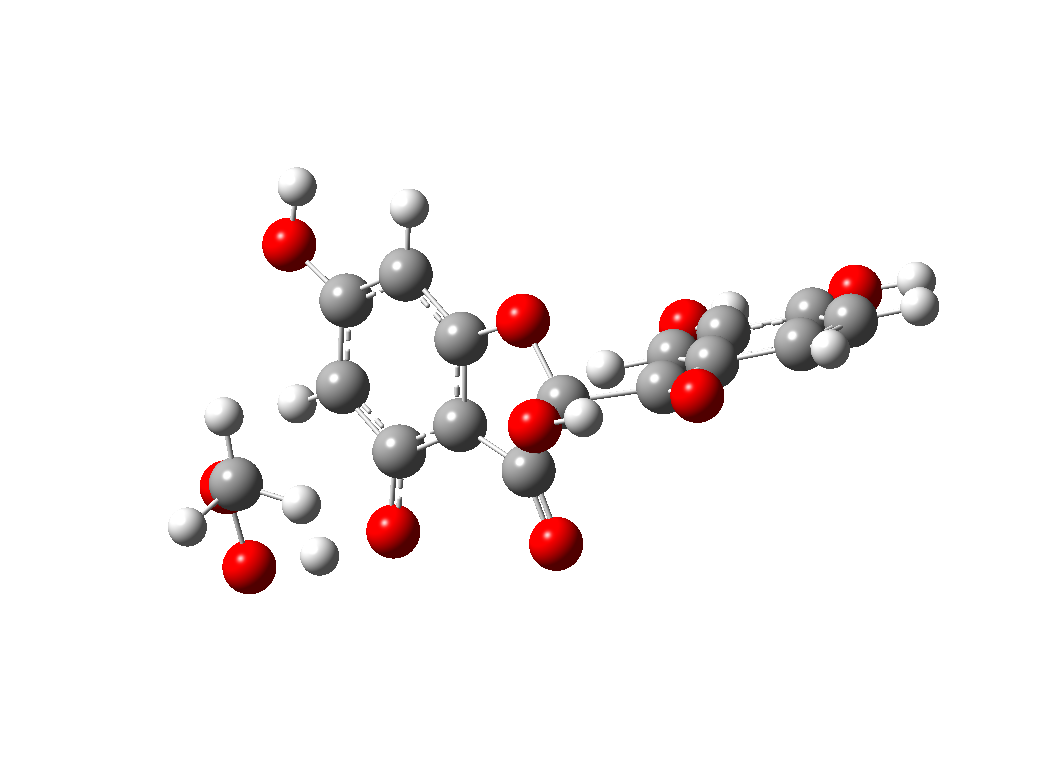 | 6 0.907506000 0.087613000 1.118381000  6 1.203867000 0.173441000 -0.234036000  6 1.495484000 -0.828133000 1.989670000  6 2.158083000 -0.715893000 -0.796430000  6 2.425436000 -1.695398000 1.438151000  6 2.757870000 -1.657419000 0.071323000  8 -0.030958000 0.964477000 1.538160000  6 -0.335322000 1.854971000 0.441392000  6 0.371893000 1.221621000 -0.793624000  6 -1.877400000 1.996154000 0.363954000  6 -2.749696000 0.839565000 0.111628000  6 -4.507402000 -1.256380000 -0.345964000  6 -5.005923000 0.009382000 -0.071589000  6 -3.127502000 -1.483516000 -0.394810000  6 -4.129003000 1.055507000 0.156264000  6 -2.254642000 -0.439405000 -0.166343000  8 -2.307061000 3.113984000 0.553686000  8 0.211797000 1.630317000 -1.912970000  8 -5.280123000 -2.345045000 -0.582291000  8 0.228203000 3.082032000 0.658073000  8 3.059798000 -2.628477000 2.171332000  8 2.506180000 -0.657543000 -2.040180000  8 -2.643783000 -2.721065000 -0.660884000  1 1.227931000 -0.853630000 3.037124000  1 3.461915000 -2.374192000 -0.323479000  1 -6.076510000 0.168027000 -0.039458000  1 -4.505118000 2.044588000 0.370871000  1 -1.199701000 -0.661755000 -0.220904000  1 -6.216500000 -2.120027000 -0.550932000  1 -0.499445000 3.693113000 0.862560000  1 2.758999000 -2.601961000 3.086994000  1 3.699257000 -0.476318000 -2.088204000  1 -3.381074000 -3.320721000 -0.823920000  8 4.890211000 -0.211893000 -0.520314000  8 4.806438000 -0.149447000 -1.873334000  6 4.805333000 1.110516000 0.032242000  1 3.912116000 1.609291000 -0.339512000  1 4.763138000 0.973337000 1.109846000  1 5.697884000 1.665770000 -0.252460000 |
| --- | --- |

Optimized geometry and Cartesian coordinates of 2-(3,4-dihydroxybenzoyl)-2,4,6-trihydroxy-3(2*H*)-benzofuranone (**4**) 7-OH∙∙∙^•^OOCH_3_ TS at SMD/um052x/6-311++g(d,p) level of theory in pentyl ethanoate

| 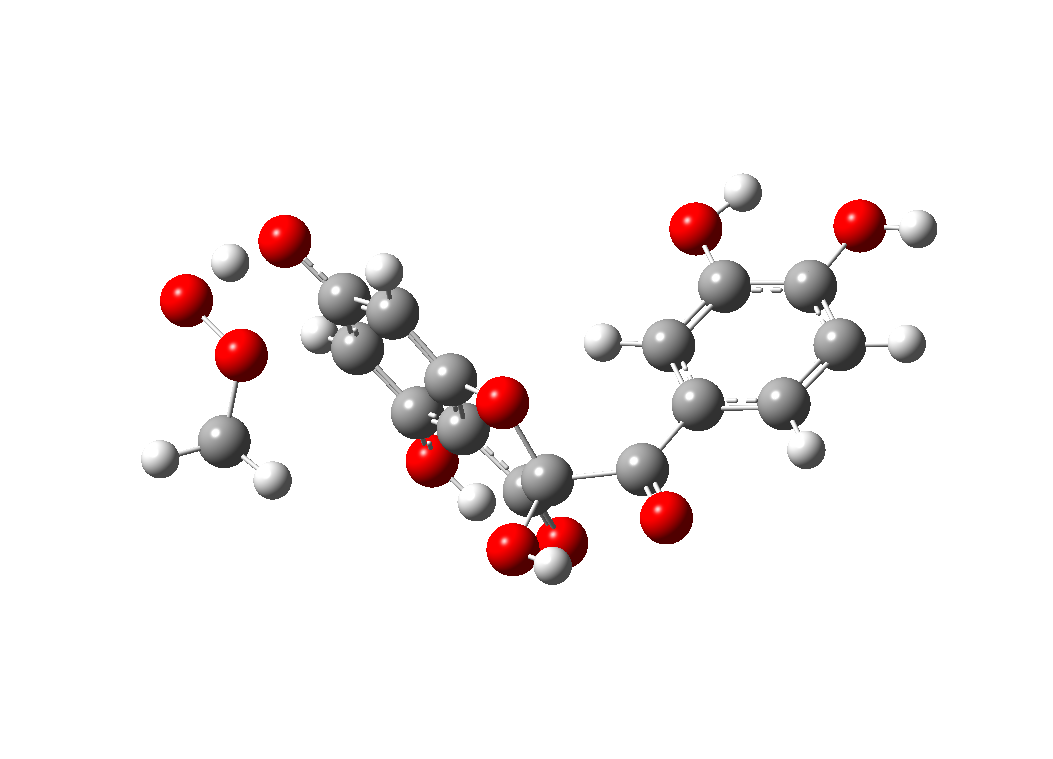 | 6 -1.172528000 0.138567000 -0.373138000  6 -1.040119000 0.122273000 1.006866000  6 -2.028749000 -0.723355000 -1.029354000  6 -1.766328000 -0.783006000 1.814386000  6 -2.799899000 -1.605086000 -0.207127000  6 -2.651325000 -1.635912000 1.206948000  8 -0.360667000 1.045652000 -0.980094000  6 0.275790000 1.842130000 0.045772000  6 -0.050512000 1.103214000 1.370160000  6 1.773472000 1.988309000 -0.335240000  6 2.677334000 0.831682000 -0.373614000  6 4.494164000 -1.260940000 -0.455772000  6 4.931506000 0.036344000 -0.683272000  6 3.145457000 -1.522264000 -0.186311000  6 4.024994000 1.080939000 -0.643317000  6 2.242866000 -0.479272000 -0.145586000  8 2.129200000 3.115262000 -0.603505000  8 0.405267000 1.372946000 2.457569000  8 5.299287000 -2.350599000 -0.473558000  8 -0.301772000 3.078997000 0.122321000  8 -3.683954000 -2.361407000 -0.775191000  8 -1.594005000 -0.779506000 3.143899000  8 2.722254000 -2.790451000 0.030486000  1 -2.115749000 -0.761798000 -2.103462000  1 -3.251475000 -2.326883000 1.780556000  1 5.978597000 0.220181000 -0.888730000  1 4.354184000 2.094858000 -0.814917000  1 1.211739000 -0.730085000 0.052968000  1 6.211413000 -2.106864000 -0.666479000  1 0.319895000 3.704506000 -0.287731000  1 -4.535446000 -1.625752000 -1.317276000  1 -0.924343000 -0.123535000 3.385516000  1 3.473678000 -3.391175000 -0.037446000  8 -5.230851000 -0.764308000 -1.575938000  8 -4.403076000 0.312716000 -1.562123000  6 -4.743965000 1.175052000 -0.465545000  1 -4.696869000 0.618514000 0.468669000  1 -4.006046000 1.973296000 -0.488675000  1 -5.745994000 1.568267000 -0.629049000 |
| --- | --- |

Optimized geometry and Cartesian coordinates of 2-(3,4-dihydroxybenzoyl)-2,4,6-trihydroxy-3(2*H*)-benzofuranone (**4**) 3’-OH∙∙∙^•^OOCH_3_ TS at SMD/um052x/6-311++g(d,p) level of theory in pentyl ethanoate

| 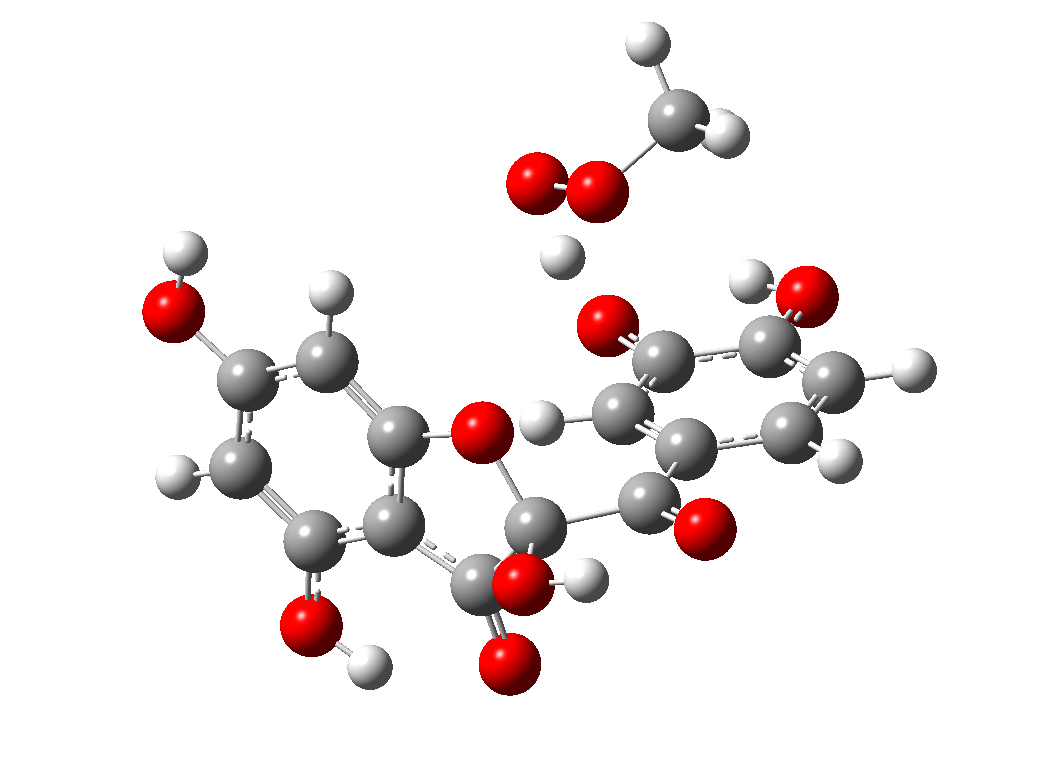 | 6 -1.998538000 -0.069927000 -0.867259000  6 -2.337977000 0.375844000 0.410360000  6 -2.404940000 -1.288453000 -1.352620000  6 -3.119860000 -0.414197000 1.262111000  6 -3.197681000 -2.055111000 -0.483071000  6 -3.562225000 -1.640801000 0.803556000  8 -1.196995000 0.807025000 -1.532257000  6 -1.060351000 2.002835000 -0.731739000  6 -1.705219000 1.638223000 0.634106000  6 0.444263000 2.376740000 -0.719307000  6 1.457737000 1.499676000 -0.109166000  6 3.516206000 -0.039286000 0.978011000  6 3.814656000 1.199761000 0.422408000  6 2.179332000 -0.522848000 0.995610000  6 2.793480000 1.954532000 -0.116354000  6 1.157023000 0.269177000 0.450109000  8 0.730838000 3.421702000 -1.259979000  8 -1.683034000 2.338724000 1.623393000  8 4.475738000 -0.803885000 1.497773000  8 -1.785071000 3.031449000 -1.265755000  8 -3.654654000 -3.262765000 -0.862168000  8 -3.428096000 0.009166000 2.495693000  8 1.967469000 -1.702198000 1.538714000  1 -2.132220000 -1.639892000 -2.337501000  1 -4.169102000 -2.284300000 1.423222000  1 4.836377000 1.551558000 0.421353000  1 3.009511000 2.918467000 -0.553939000  1 0.160124000 -0.143079000 0.479724000  1 4.053596000 -1.596421000 1.866610000  1 -1.155554000 3.630442000 -1.699292000  1 -3.344510000 -3.476001000 -1.749221000  1 -3.016456000 0.871499000 2.652922000  1 1.706228000 -2.440361000 0.682983000  6 3.380423000 -2.210754000 -1.668993000  1 3.415746000 -3.112141000 -2.278795000  1 3.648400000 -1.334725000 -2.254189000  1 4.030076000 -2.317337000 -0.801097000  8 2.030079000 -1.989525000 -1.239552000  8 1.646198000 -2.984715000 -0.408279000 |
| --- | --- |

Optimized geometry and Cartesian coordinates of 2-(3,4-dihydroxybenzoyl)-2,4,6-trihydroxy-3(2*H*)-benzofuranone (**4**) 4’-OH∙∙∙^•^OOCH_3_ TS at SMD/um052x/6-311++g(d,p) level of theory in pentyl ethanoate

| 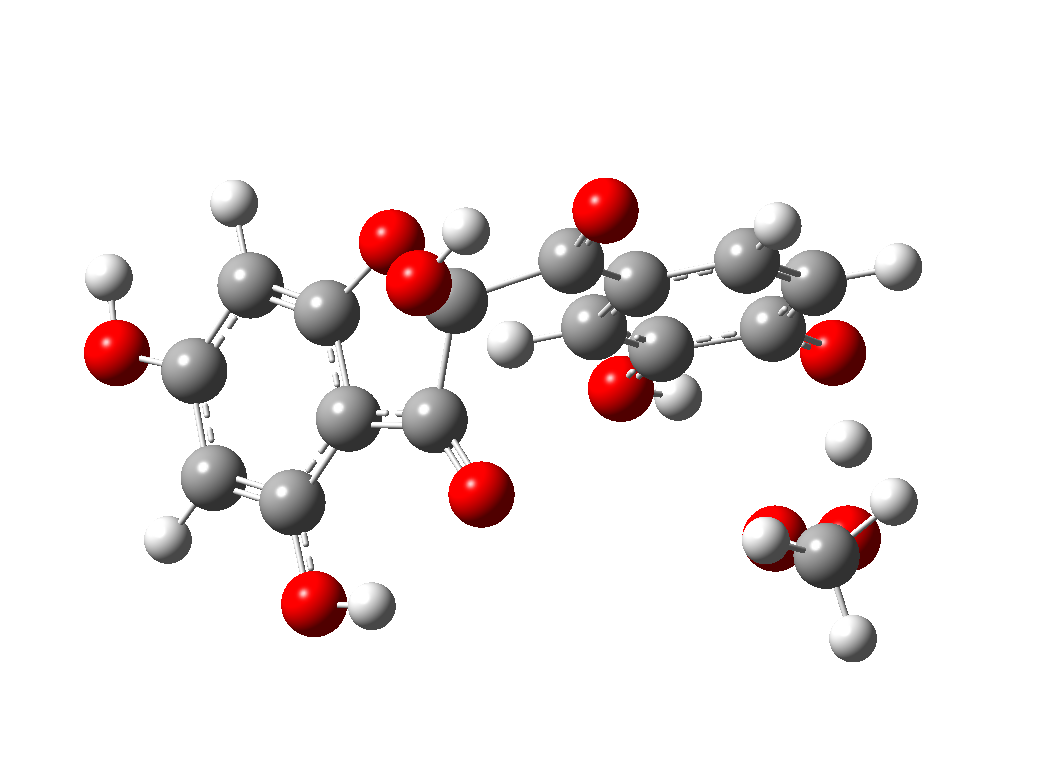 | 6 -2.573798000 0.319410000 -0.639237000  6 -2.296636000 -0.028185000 0.683028000  6 -3.383830000 -0.440057000 -1.446486000  6 -2.846301000 -1.184580000 1.249843000  6 -3.932538000 -1.587079000 -0.848882000  6 -3.680467000 -1.967615000 0.474097000  8 -1.921112000 1.449364000 -1.031647000  6 -1.267290000 2.022743000 0.123038000  6 -1.392306000 0.936834000 1.226803000  6 0.168177000 2.412446000 -0.307163000  8 0.404906000 3.597944000 -0.350179000  8 -0.853006000 0.983937000 2.311488000  8 -1.934913000 3.131996000 0.561059000  8 -4.749631000 -2.397575000 -1.546362000  8 -2.569600000 -1.524696000 2.516072000  1 -3.591640000 -0.173812000 -2.473578000  1 -4.130621000 -2.865560000 0.870069000  1 -1.449596000 3.908017000 0.237796000  1 -4.868463000 -2.066159000 -2.443514000  1 -1.961518000 -0.875660000 2.898975000  6 1.188720000 1.393355000 -0.631276000  6 0.879046000 0.048471000 -0.778963000  6 2.519472000 1.847224000 -0.775285000  6 1.897583000 -0.855948000 -1.058341000  1 -0.124801000 -0.339655000 -0.702327000  6 3.529481000 0.957927000 -1.032688000  1 2.726601000 2.901282000 -0.665576000  6 3.246690000 -0.416320000 -1.164435000  8 1.607937000 -2.147291000 -1.224806000  1 4.557138000 1.279519000 -1.130745000  8 4.175957000 -1.312845000 -1.396863000  1 2.425658000 -2.622313000 -1.433981000  1 4.413878000 -1.837856000 -0.392304000  8 4.410625000 -2.174755000 0.789751000  8 3.347632000 -1.484451000 1.258530000  6 3.783420000 -0.419908000 2.119631000  1 2.899755000 0.186630000 2.302765000  1 4.151944000 -0.852955000 3.048259000  1 4.564765000 0.153176000 1.625704000 |
| --- | --- |

Optimized geometry and Cartesian coordinates of 2-(3,4-dihydroxybenzoyl)-2,4,6-trihydroxy-3(2*H*)-benzofuranone (**4**) 3’,4’-diOH∙∙∙O_2_^•−^ TS at SMD/um052x/6-311++g(d,p) level of theory in pentyl ethanoate

| 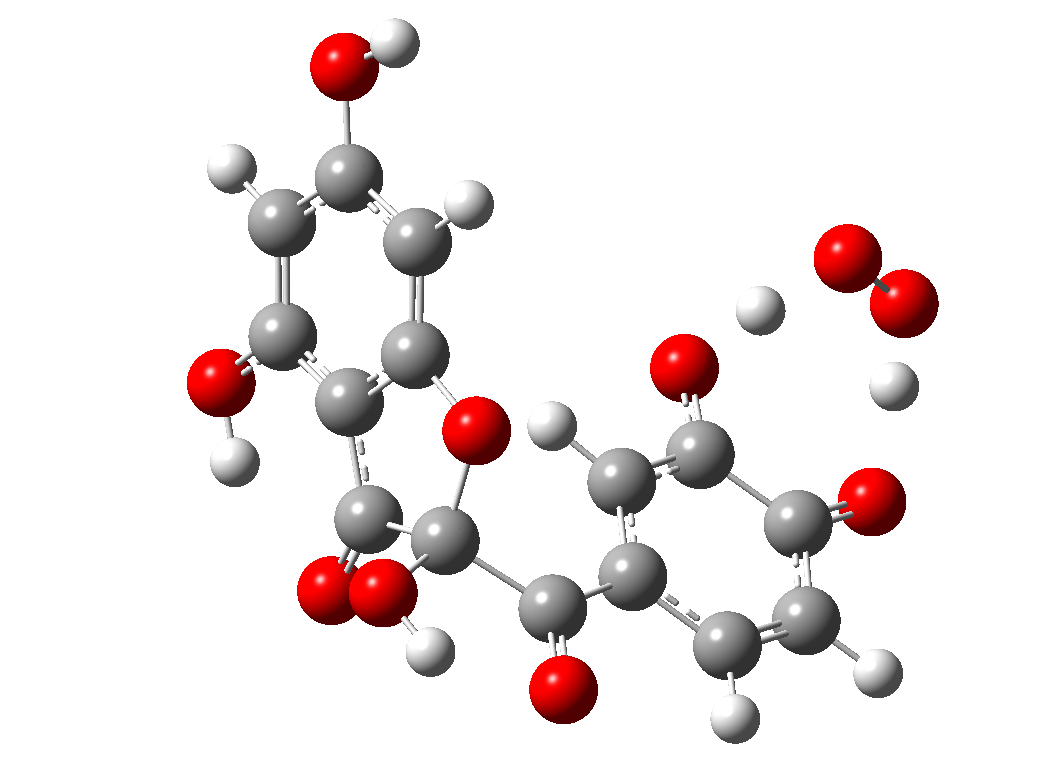 | 6 -2.109182000 -0.168302000 -0.887875000  6 -2.314065000 -0.027244000 0.483897000  6 -2.466532000 -1.309606000 -1.566615000  6 -2.894982000 -1.053719000 1.235231000  6 -3.060314000 -2.317709000 -0.792510000  6 -3.282656000 -2.210926000 0.585387000  8 -1.498479000 0.910467000 -1.440702000  6 -1.375193000 1.940978000 -0.429551000  6 -1.793684000 1.242286000 0.890386000  6 0.067319000 2.520336000 -0.492395000  8 0.123073000 3.702011000 -0.798822000  8 -1.730223000 1.736849000 1.996214000  8 -2.272329000 2.947239000 -0.669296000  8 -3.456235000 -3.472926000 -1.363939000  8 -3.071035000 -0.913829000 2.557831000  1 -2.298535000 -1.428892000 -2.627617000  1 -3.734070000 -3.031979000 1.122210000  1 -1.737067000 3.711116000 -0.952001000  1 -3.240237000 -3.471030000 -2.302893000  1 -2.707670000 -0.060148000 2.836522000  6 1.232545000 1.704202000 -0.218598000  6 1.164305000 0.346389000 0.086924000  6 2.506330000 2.332234000 -0.272524000  6 2.309932000 -0.403351000 0.359197000  1 0.229664000 -0.191808000 0.141954000  6 3.647728000 1.628978000 -0.026995000  1 2.550600000 3.385871000 -0.510441000  6 3.621763000 0.231015000 0.288811000  8 2.207451000 -1.664977000 0.713864000  1 4.617949000 2.106038000 -0.064928000  8 4.687887000 -0.411717000 0.512194000  1 2.849103000 -2.304637000 0.001500000  8 3.636341000 -2.900609000 -0.726104000  8 4.765492000 -2.876530000 0.042034000  1 4.814858000 -1.895743000 0.312874000 |
| --- | --- |

Optimized geometry and Cartesian coordinates of alphitonin (**5**) 5-OH∙∙∙^•^OOH TS at SMD/um052x/6-311++g(d,p) level of theory in pentyl ethanoate

| 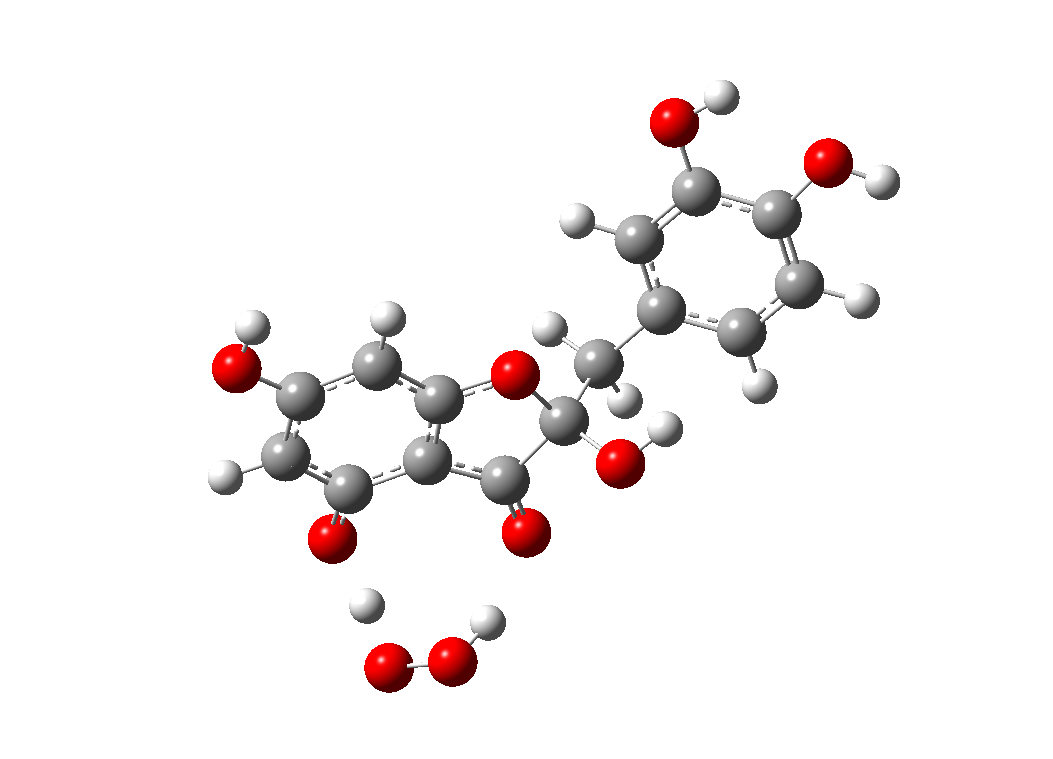 | 6 -1.247734000 1.059987000 0.265701000  6 -2.109288000 0.059319000 -0.178382000  6 -1.652243000 2.374427000 0.462975000  6 -3.466728000 0.380611000 -0.496273000  6 -2.991841000 2.669165000 0.197276000  6 -3.887526000 1.705333000 -0.266730000  8 0.014427000 0.651761000 0.475155000  6 0.099455000 -0.765112000 0.140718000  6 -1.344476000 -1.152362000 -0.228887000  6 1.041001000 -0.930951000 -1.051400000  8 -1.670101000 -2.303676000 -0.466533000  8 0.469958000 -1.481718000 1.250590000  8 -3.482245000 3.910260000 0.372941000  8 -4.275227000 -0.459488000 -1.045868000  1 -0.958635000 3.126718000 0.813156000  1 -4.908864000 1.978387000 -0.488939000  1 1.431860000 -1.431927000 1.342650000  1 -2.795441000 4.504291000 0.696367000  1 -4.508601000 -1.570504000 -0.501124000  1 0.930839000 -1.959180000 -1.397346000  6 2.469834000 -0.655133000 -0.662694000  6 3.297728000 -1.695346000 -0.247160000  6 2.961676000 0.652455000 -0.663168000  6 4.603829000 -1.437028000 0.167266000  1 2.934402000 -2.715052000 -0.263613000  6 4.257296000 0.912316000 -0.257649000  1 2.338762000 1.477967000 -0.979971000  6 5.079252000 -0.139509000 0.162106000  1 5.251838000 -2.244681000 0.485305000  1 0.709982000 -0.260213000 -1.844611000  8 6.340100000 0.225511000 0.538819000  1 6.849943000 -0.542337000 0.816271000  8 4.730363000 2.183920000 -0.267400000  1 5.643520000 2.176356000 0.042552000  8 -4.836166000 -2.410712000 0.148066000  8 -3.859236000 -2.577474000 1.077888000  1 -3.036014000 -2.675923000 0.547831000 |
| --- | --- |

Optimized geometry and Cartesian coordinates of alphitonin (**5**) 7-OH∙∙∙^•^OOH TS at SMD/um052x/6-311++g(d,p) level of theory in pentyl ethanoate

| 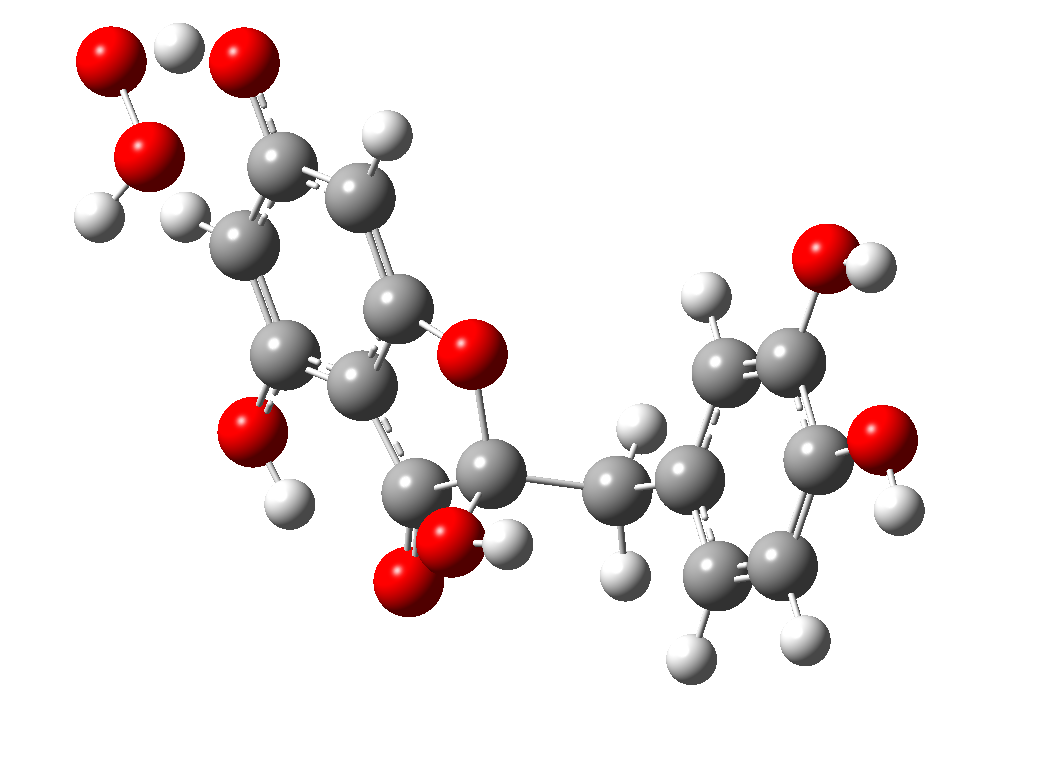 | 6 1.428265000 -0.139433000 -0.167371000  6 1.965486000 1.142725000 -0.187484000  6 2.207422000 -1.245474000 -0.445882000  6 3.321615000 1.385707000 -0.480822000  6 3.576961000 -0.998616000 -0.745892000  6 4.131238000 0.309430000 -0.753551000  8 0.112184000 -0.164062000 0.135157000  6 -0.346305000 1.203353000 0.364133000  6 0.912806000 2.066578000 0.142262000  6 -1.431646000 1.535396000 -0.656725000  8 0.960941000 3.275272000 0.225530000  8 -0.744774000 1.346846000 1.671463000  8 4.351359000 -2.013036000 -1.020625000  8 3.796370000 2.640384000 -0.485944000  1 1.822714000 -2.253420000 -0.450900000  1 5.178331000 0.433879000 -0.987157000  1 -1.640230000 0.990314000 1.758975000  1 4.566368000 -2.627680000 -0.029588000  1 3.082667000 3.257223000 -0.266087000  1 -1.615873000 2.607534000 -0.580165000  6 -2.698231000 0.764173000 -0.390457000  6 -3.707087000 1.319867000 0.392425000  6 -2.855836000 -0.530351000 -0.891177000  6 -4.864047000 0.594620000 0.674633000  1 -3.605987000 2.329877000 0.768690000  6 -4.003322000 -1.251171000 -0.617584000  1 -2.088199000 -0.986308000 -1.501556000  6 -5.009559000 -0.684662000 0.172626000  1 -5.652647000 1.029143000 1.276603000  1 -1.041900000 1.322170000 -1.652242000  8 -6.100758000 -1.479787000 0.380413000  1 -6.755966000 -1.027121000 0.920693000  8 -4.152652000 -2.503512000 -1.116849000  1 -5.007292000 -2.849931000 -0.835009000  8 4.717134000 -2.946635000 1.098273000  8 3.675407000 -2.340951000 1.730649000  1 4.069767000 -1.574679000 2.178412000 |
| --- | --- |

Optimized geometry and Cartesian coordinates of alphitonin (**5**) 3’-OH∙∙∙^•^OOH TS at SMD/um052x/6-311++g(d,p) level of theory in pentyl ethanoate

| 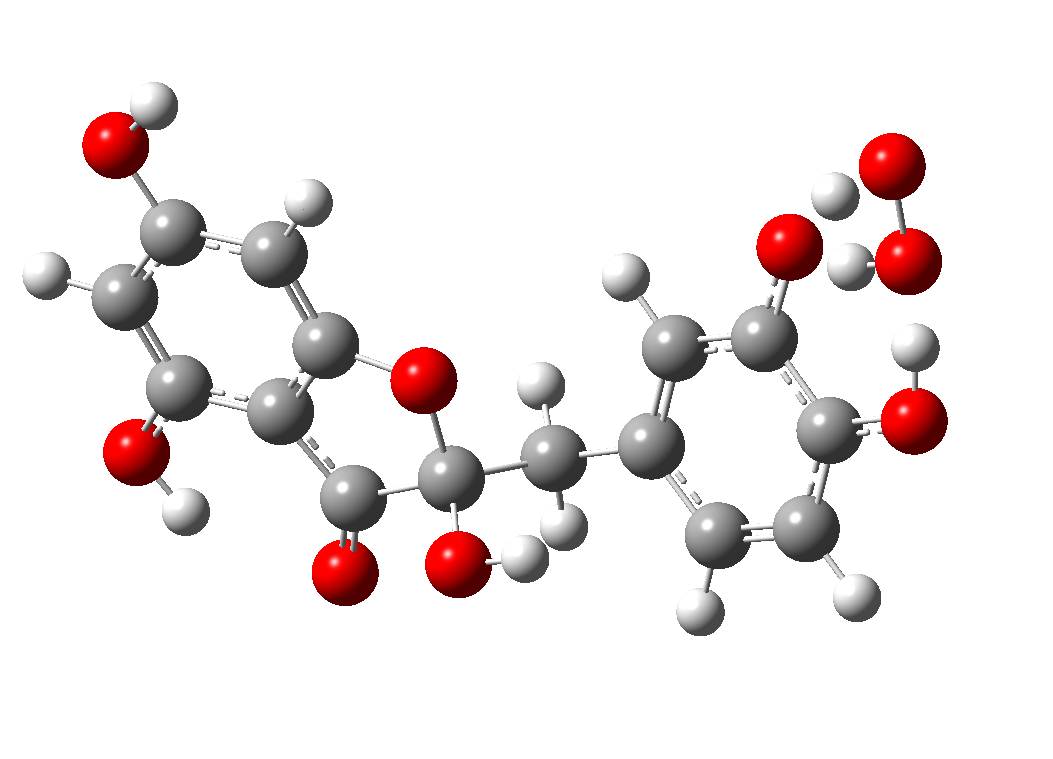 | 6 2.257908000 0.562910000 -0.438732000  6 3.020278000 -0.458482000 0.127523000  6 2.784007000 1.810529000 -0.690949000  6 4.366464000 -0.259388000 0.455837000  6 4.132493000 1.984362000 -0.348965000  6 4.929015000 0.979406000 0.215698000  8 0.981035000 0.189140000 -0.685515000  6 0.808727000 -1.198585000 -0.272165000  6 2.195125000 -1.614319000 0.267409000  6 -0.247947000 -1.251083000 0.828210000  8 2.466072000 -2.708747000 0.721944000  8 0.520417000 -1.979533000 -1.368351000  8 4.742821000 3.167856000 -0.554757000  8 5.088201000 -1.253630000 0.994067000  1 2.201976000 2.607987000 -1.131267000  1 5.962884000 1.182440000 0.452860000  1 -0.394179000 -1.819598000 -1.635872000  1 4.131776000 3.795238000 -0.956579000  1 4.528970000 -2.040192000 1.080042000  1 -0.224517000 -2.260295000 1.240064000  6 -1.621831000 -0.929953000 0.301039000  6 -2.491912000 -1.969359000 -0.084011000  6 -2.036404000 0.380204000 0.143631000  6 -3.743369000 -1.714759000 -0.619257000  1 -2.175226000 -2.995888000 0.052192000  6 -3.303363000 0.665995000 -0.388680000  1 -1.397673000 1.209499000 0.418753000  6 -4.158227000 -0.398503000 -0.777185000  1 -4.405190000 -2.518769000 -0.908350000  1 0.045350000 -0.551901000 1.611699000  8 -5.358718000 -0.118642000 -1.294056000  1 -5.443278000 0.845188000 -1.344974000  8 -3.742128000 1.903337000 -0.571420000  1 -4.129132000 2.275479000 0.391663000  8 -4.644883000 0.988370000 1.911011000  1 -3.805542000 0.785987000 2.356041000  8 -4.583068000 2.305722000 1.604240000 |
| --- | --- |

Optimized geometry and Cartesian coordinates of alphitonin (**5**) 4’-OH∙∙∙^•^OOH TS at SMD/um052x/6-311++g(d,p) level of theory in pentyl ethanoate

| 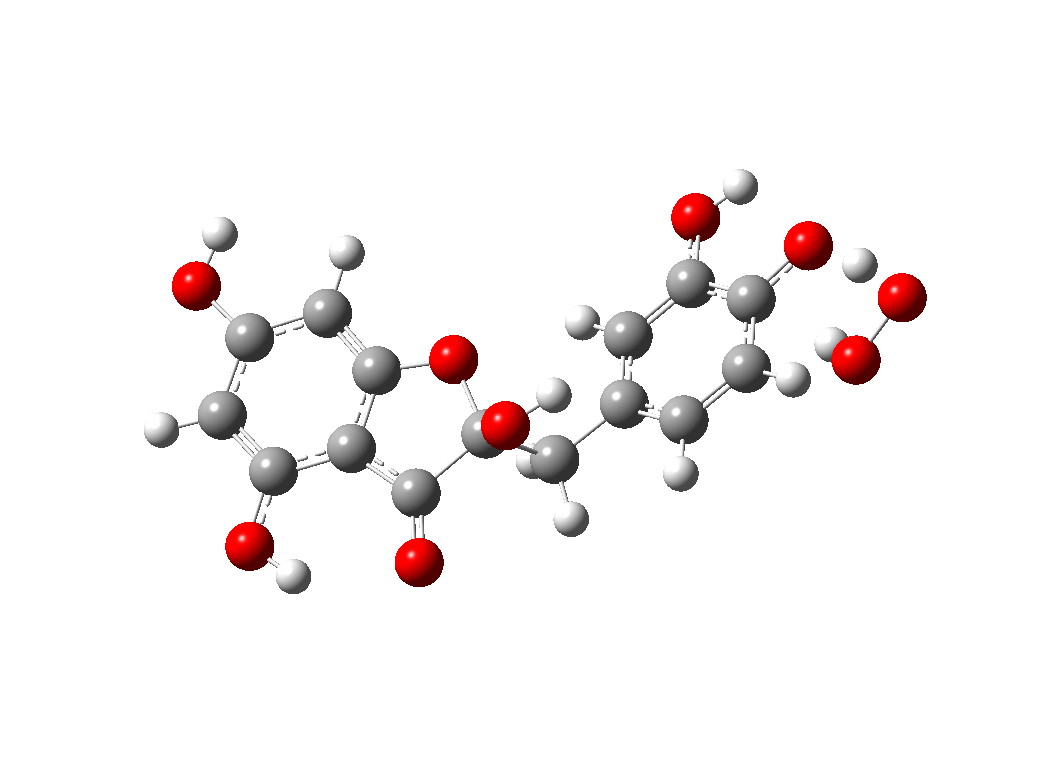 | 6 -2.433565000 0.626396000 0.285436000  6 -3.139340000 -0.520116000 -0.078419000  6 -3.030275000 1.866631000 0.335073000  6 -4.498906000 -0.456636000 -0.405648000  6 -4.393043000 1.901392000 0.007053000  6 -5.133035000 0.770114000 -0.362047000  8 -1.132305000 0.376597000 0.559414000  6 -0.882693000 -1.049616000 0.399024000  6 -2.245973000 -1.633003000 -0.037307000  6 0.171048000 -1.241424000 -0.690419000  8 -2.453439000 -2.803133000 -0.292440000  8 -0.548497000 -1.603854000 1.614302000  8 -5.074822000 3.063635000 0.032614000  8 -5.166312000 -1.565693000 -0.757496000  1 -2.487287000 2.758119000 0.616986000  1 -6.180571000 0.868711000 -0.604166000  1 0.357586000 -1.352540000 1.837454000  1 -4.497331000 3.784673000 0.306462000  1 -4.563329000 -2.323206000 -0.722623000  1 0.219935000 -2.310530000 -0.898083000  6 1.515853000 -0.724615000 -0.258105000  6 2.425506000 -1.591033000 0.377621000  6 1.838386000 0.615975000 -0.441396000  6 3.641703000 -1.123708000 0.827830000  1 2.170238000 -2.636186000 0.494881000  6 3.058896000 1.093642000 0.008563000  1 1.147370000 1.293833000 -0.922778000  6 3.978953000 0.226740000 0.652628000  1 4.357319000 -1.775522000 1.310309000  1 -0.172624000 -0.726217000 -1.587654000  8 5.115477000 0.757326000 1.083063000  1 5.900673000 0.571396000 0.333804000  8 3.393039000 2.382389000 -0.163820000  1 4.233181000 2.532633000 0.294772000  8 5.510953000 -0.207639000 -1.527401000  1 5.186809000 0.483112000 -2.128507000  8 6.491773000 0.383781000 -0.804508000 |
| --- | --- |

Optimized geometry and Cartesian coordinates of alphitonin (**5**) 5-OH∙∙∙^•^OOCH_3_ TS at SMD/um052x/6-311++g(d,p) level of theory in pentyl ethanoate

| 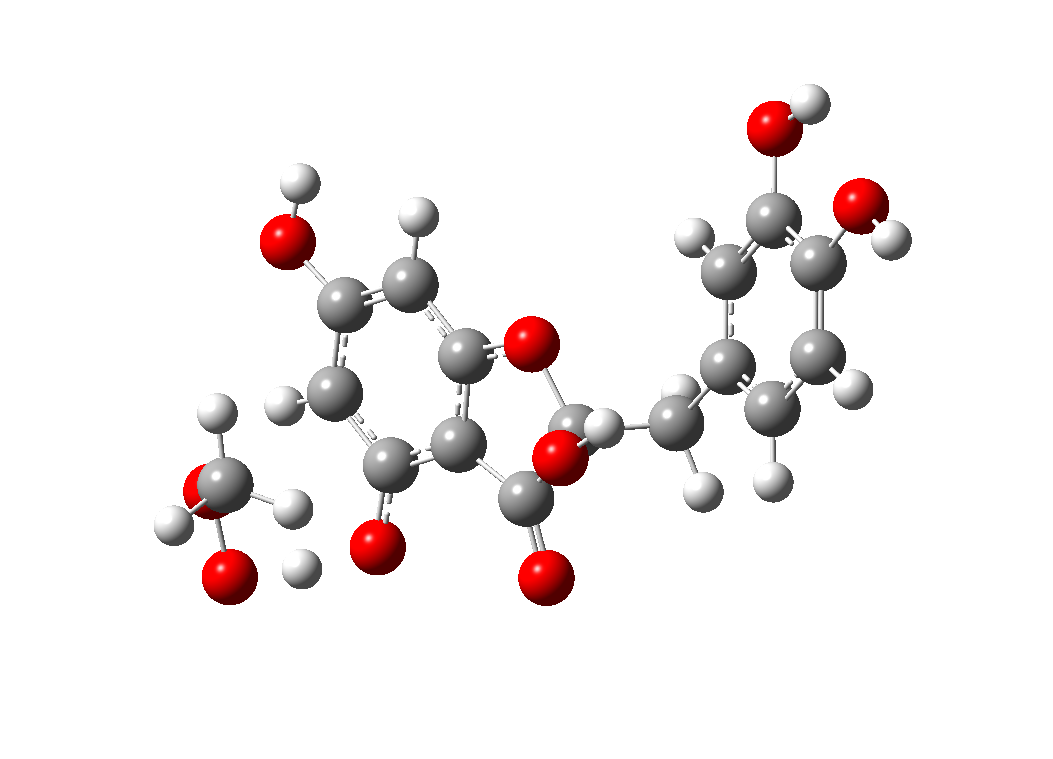 | 6 -1.049469000 0.759624000 -0.133196000  6 -1.769041000 -0.344293000 -0.571672000  6 -1.600512000 2.037268000 0.009541000  6 -3.142670000 -0.199705000 -0.894118000  6 -2.936902000 2.176375000 -0.321329000  6 -3.709347000 1.089488000 -0.773288000  8 0.236243000 0.508862000 0.154174000  6 0.490370000 -0.912758000 -0.067256000  6 -0.866186000 -1.476757000 -0.559274000  6 1.570346000 -1.055464000 -1.135164000  8 -1.014795000 -2.636194000 -0.854394000  8 0.803742000 -1.511374000 1.130202000  8 -3.581081000 3.356136000 -0.233393000  8 -3.881006000 -1.204823000 -1.244449000  1 -1.001206000 2.867385000 0.358434000  1 -4.740239000 1.248981000 -1.050106000  1 1.742169000 -1.362757000 1.312105000  1 -2.978880000 4.048612000 0.061588000  1 -4.760270000 -1.316311000 -0.441529000  1 1.571861000 -2.103728000 -1.435983000  6 2.928529000 -0.657780000 -0.618075000  6 3.788186000 -1.622930000 -0.099627000  6 3.323209000 0.682512000 -0.611625000  6 5.029645000 -1.259975000 0.420426000  1 3.500091000 -2.666447000 -0.115432000  6 4.554990000 1.046675000 -0.099691000  1 2.675342000 1.452280000 -1.008601000  6 5.410005000 0.068401000 0.419624000  1 5.701381000 -2.011138000 0.817630000  1 1.281620000 -0.450824000 -1.995289000  8 6.603052000 0.533689000 0.896430000  1 7.147221000 -0.190954000 1.220304000  8 4.933880000 2.350028000 -0.103395000  1 5.820694000 2.414892000 0.269650000  8 -5.058084000 -0.128375000 1.128211000  8 -5.424354000 -1.296040000 0.541504000  6 -4.181765000 -0.393075000 2.233556000  1 -3.350196000 -1.014395000 1.905924000  1 -3.838462000 0.580763000 2.573897000  1 -4.748238000 -0.897271000 3.015290000 |
| --- | --- |

Optimized geometry and Cartesian coordinates of alphitonin (**5**) 7-OH∙∙∙^•^OOCH_3_ TS at SMD/um052x/6-311++g(d,p) level of theory in pentyl ethanoate

| 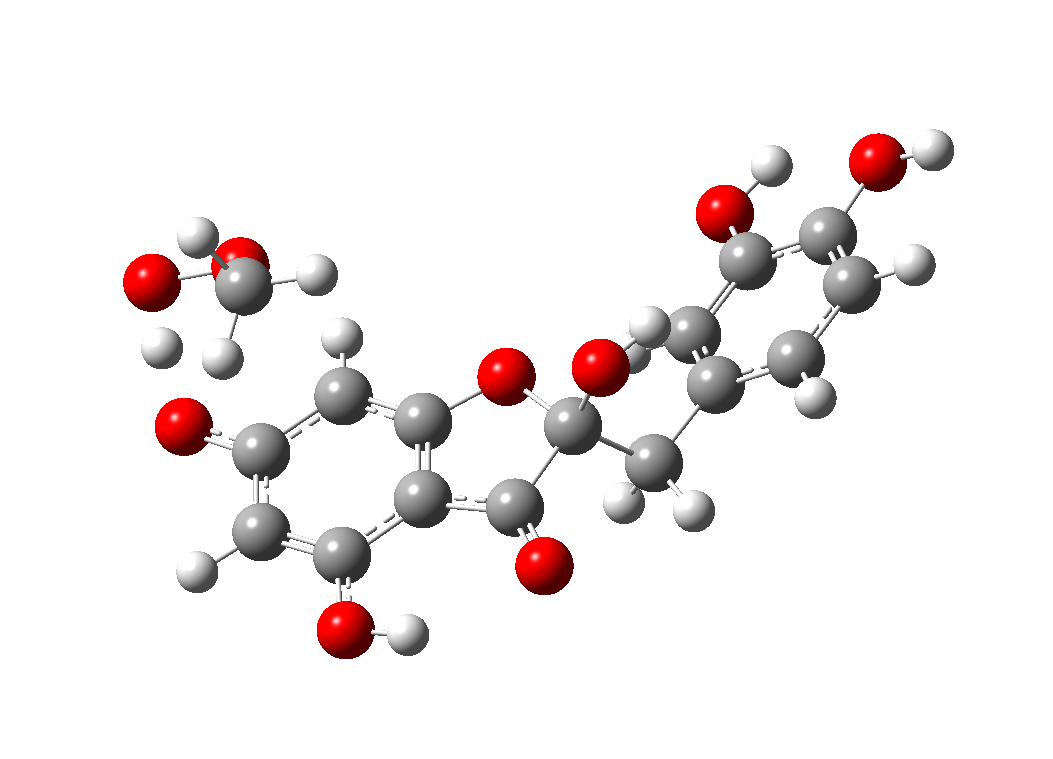 | 6 1.307378000 -0.023461000 -0.331952000  6 1.814783000 1.263713000 -0.256944000  6 2.099085000 -1.083380000 -0.749627000  6 3.154785000 1.562737000 -0.597770000  6 3.461142000 -0.784009000 -1.060176000  6 3.976493000 0.539986000 -0.994321000  8 0.008213000 -0.113806000 0.017889000  6 -0.475741000 1.221752000 0.366565000  6 0.758535000 2.129614000 0.184334000  6 -1.589183000 1.605121000 -0.605220000  8 0.780384000 3.328765000 0.369885000  8 -0.849995000 1.249513000 1.687635000  8 4.251675000 -1.765062000 -1.367704000  8 3.592481000 2.827762000 -0.514636000  1 1.721929000 -2.087092000 -0.863765000  1 5.013319000 0.711692000 -1.243291000  1 -1.742545000 0.882589000 1.761237000  1 4.321272000 -2.519081000 -0.390369000  1 2.872037000 3.399876000 -0.210549000  1 -1.784182000 2.667320000 -0.453834000  6 -2.840310000 0.802057000 -0.361073000  6 -3.843333000 1.303041000 0.464911000  6 -2.989828000 -0.466575000 -0.926905000  6 -4.986962000 0.549623000 0.726442000  1 -3.745985000 2.292901000 0.892398000  6 -4.124441000 -1.214927000 -0.674350000  1 -2.226432000 -0.879889000 -1.571941000  6 -5.125012000 -0.702935000 0.159443000  1 -5.770494000 0.941048000 1.363721000  1 -1.220048000 1.462919000 -1.621125000  8 -6.202700000 -1.522007000 0.342541000  1 -6.862845000 -1.098618000 0.900348000  8 -4.267127000 -2.441307000 -1.236287000  1 -5.116320000 -2.809664000 -0.966284000  8 4.331868000 -2.953869000 0.672255000  8 3.203440000 -2.437115000 1.225021000  6 3.551841000 -1.470506000 2.227871000  1 4.171482000 -0.690640000 1.788742000  1 2.605261000 -1.069979000 2.582218000  1 4.082239000 -1.977485000 3.032324000 |
| --- | --- |

Optimized geometry and Cartesian coordinates of alphitonin (**5**) 3’-OH∙∙∙^•^OOCH_3_ TS at SMD/um052x/6-311++g(d,p) level of theory in pentyl ethanoate

| 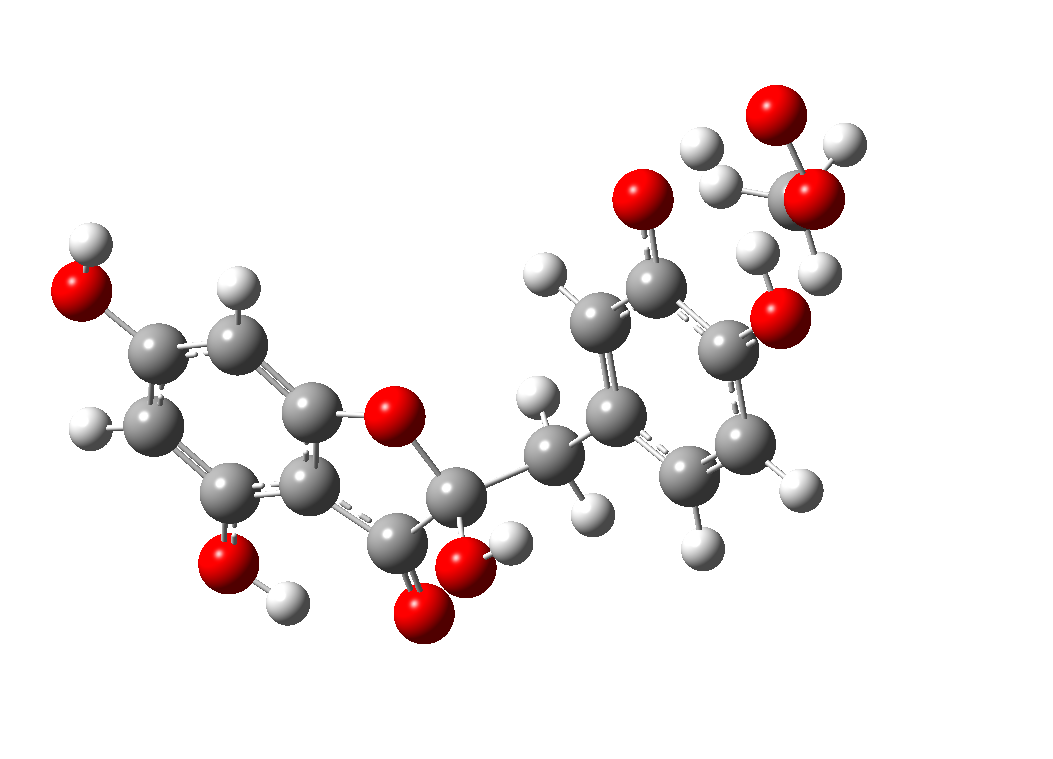 | 6 2.492799000 0.567389000 -0.462175000  6 3.240234000 -0.437901000 0.150962000  6 3.016736000 1.817241000 -0.708514000  6 4.567755000 -0.218864000 0.537209000  6 4.346794000 2.010519000 -0.310067000  6 5.127498000 1.022704000 0.304895000  8 1.232198000 0.176880000 -0.759179000  6 1.054010000 -1.205353000 -0.329287000  6 2.421581000 -1.600866000 0.269963000  6 -0.041857000 -1.247325000 0.732587000  8 2.684786000 -2.686972000 0.748793000  8 0.811059000 -2.006304000 -1.421919000  8 4.953740000 3.197457000 -0.506829000  8 5.274683000 -1.197165000 1.122648000  1 2.446274000 2.601800000 -1.185754000  1 6.147325000 1.240622000 0.585463000  1 -0.096763000 -1.863010000 -1.719872000  1 4.354074000 3.812196000 -0.943538000  1 4.720424000 -1.988794000 1.194026000  1 -0.023495000 -2.247888000 1.165276000  6 -1.399180000 -0.950920000 0.151138000  6 -2.248990000 -2.010234000 -0.232900000  6 -1.818961000 0.349557000 -0.048442000  6 -3.480754000 -1.778834000 -0.817172000  1 -1.928501000 -3.029938000 -0.058883000  6 -3.068647000 0.617295000 -0.629372000  1 -1.199189000 1.189716000 0.235529000  6 -3.896292000 -0.468430000 -1.028068000  1 -4.127163000 -2.593779000 -1.110749000  1 0.214474000 -0.529317000 1.512103000  8 -5.075590000 -0.218975000 -1.603735000  1 -5.174734000 0.740800000 -1.686312000  8 -3.501507000 1.846252000 -0.848841000  1 -4.213523000 2.119879000 -0.015514000  8 -4.826975000 0.780389000 1.360883000  8 -4.958604000 2.078115000 1.001000000  6 -4.032572000 0.678339000 2.550681000  1 -3.843060000 -0.383615000 2.686735000  1 -3.104158000 1.230354000 2.422385000  1 -4.603232000 1.077670000 3.387899000 |
| --- | --- |

Optimized geometry and Cartesian coordinates of alphitonin (**5**) 4’-OH∙∙∙^•^OOCH_3_ TS at SMD/um052x/6-311++g(d,p) level of theory in pentyl ethanoate

| 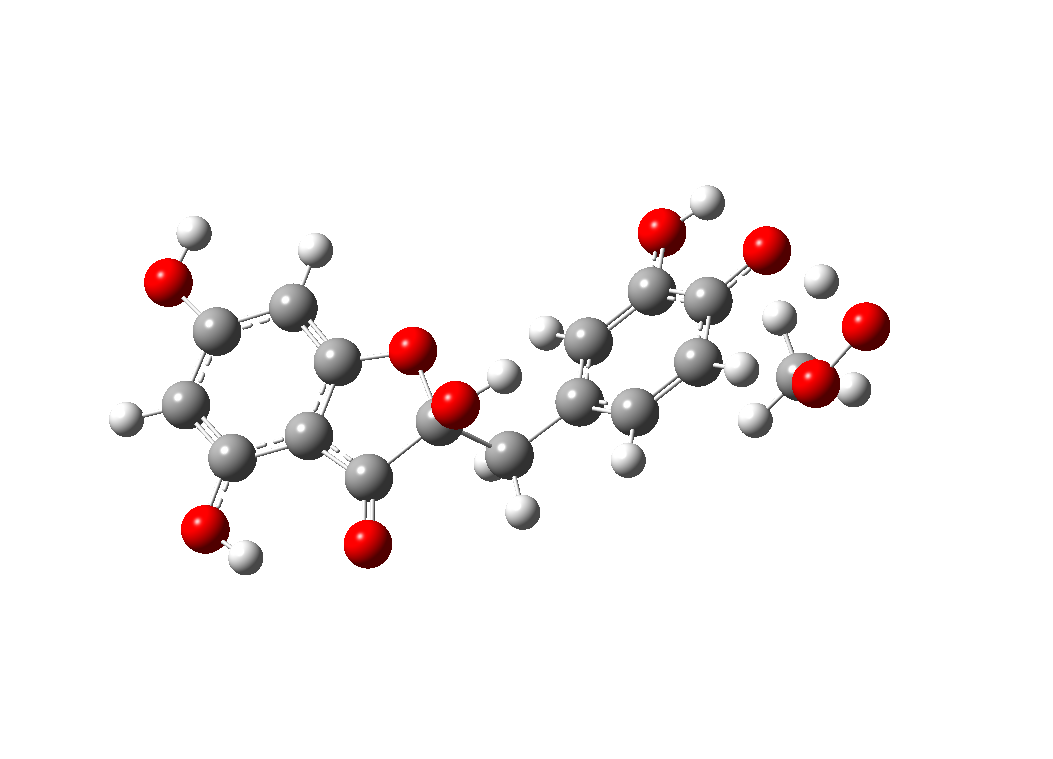 | 6 2.672721000 0.606095000 -0.326931000  6 3.367270000 -0.517534000 0.119762000  6 3.262568000 1.848650000 -0.397424000  6 4.708077000 -0.428477000 0.511728000  6 4.606098000 1.909400000 -0.001426000  6 5.334611000 0.801416000 0.451013000  8 1.388954000 0.333241000 -0.655031000  6 1.138395000 -1.085498000 -0.437255000  6 2.484813000 -1.639395000 0.081716000  6 0.044051000 -1.229971000 0.619387000  8 2.688792000 -2.796165000 0.394159000  8 0.851568000 -1.697969000 -1.636057000  8 5.279657000 3.076082000 -0.039342000  8 5.364671000 -1.516801000 0.940342000  1 2.729070000 2.723309000 -0.742649000  1 6.367477000 0.919696000 0.742221000  1 -0.045907000 -1.461006000 -1.904964000  1 4.711240000 3.781489000 -0.367366000  1 4.768665000 -2.280008000 0.907606000  1 -0.016268000 -2.289914000 0.867748000  6 -1.281385000 -0.728194000 0.117057000  6 -2.166170000 -1.615042000 -0.526248000  6 -1.610028000 0.617846000 0.245560000  6 -3.363403000 -1.164288000 -1.038165000  1 -1.907657000 -2.663705000 -0.598382000  6 -2.812556000 1.078151000 -0.263855000  1 -0.936295000 1.310015000 0.731363000  6 -3.713651000 0.190873000 -0.911707000  1 -4.060942000 -1.833649000 -1.523296000  1 0.355945000 -0.678956000 1.506936000  8 -4.836353000 0.704321000 -1.379092000  1 -5.677511000 0.386506000 -0.682195000  8 -3.157644000 2.370040000 -0.150934000  1 -3.996289000 2.484412000 -0.624175000  8 -5.326606000 -0.350346000 1.160185000  8 -6.319064000 0.060627000 0.338858000  6 -5.107782000 0.622972000 2.188446000  1 -5.983197000 0.659509000 2.835146000  1 -4.234830000 0.276468000 2.735780000  1 -4.927072000 1.599565000 1.741562000 |
| --- | --- |

Optimized geometry and Cartesian coordinates of alphitonin (**5**) 3’,4’-diOH∙∙∙O_2_^•−^ TS at SMD/um052x/6-311++g(d,p) level of theory in pentyl ethanoate

| 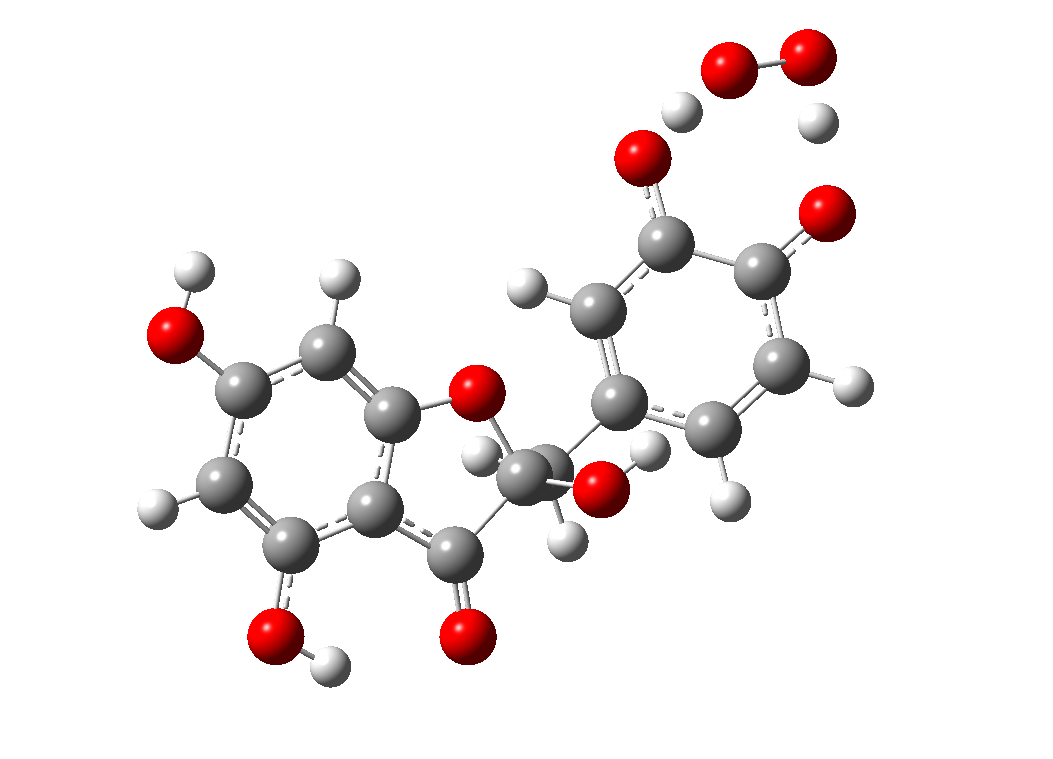 | 6 -2.229626000 0.696608000 0.209185000  6 -3.107571000 -0.380142000 0.083862000  6 -2.672726000 2.002452000 0.166484000  6 -4.482271000 -0.179321000 -0.079687000  6 -4.052164000 2.175232000 0.000783000  6 -4.962408000 1.115589000 -0.122106000  8 -0.947063000 0.314220000 0.358699000  6 -0.880536000 -1.147965000 0.328444000  6 -2.349613000 -1.588267000 0.156842000  6 -0.027838000 -1.569168000 -0.867344000  8 -2.733321000 -2.741035000 0.083007000  8 -0.416331000 -1.613775000 1.535140000  8 -4.585995000 3.413658000 -0.049027000  8 -5.309772000 -1.230736000 -0.194857000  1 -1.999102000 2.842799000 0.260196000  1 -6.014847000 1.321749000 -0.248630000  1 0.548168000 -1.526773000 1.541403000  1 -3.892824000 4.077254000 0.038254000  1 -4.787815000 -2.045514000 -0.138110000  1 -0.169447000 -2.644601000 -0.986786000  6 1.427601000 -1.245154000 -0.655023000  6 2.283499000 -2.195734000 -0.058589000  6 1.939876000 -0.003632000 -0.988800000  6 3.604245000 -1.895790000 0.192091000  1 1.897110000 -3.178530000 0.187039000  6 3.286643000 0.332755000 -0.770683000  1 1.307311000 0.747012000 -1.445998000  6 4.165200000 -0.636338000 -0.145289000  1 4.264933000 -2.626518000 0.640834000  1 -0.419331000 -1.069545000 -1.754009000  8 5.411670000 -0.390765000 0.063287000  1 5.818238000 0.894636000 0.356886000  8 3.753393000 1.501072000 -1.151857000  1 4.228199000 2.006172000 -0.204612000  8 4.759589000 2.402348000 0.807634000  8 6.013525000 1.884402000 0.679536000 |
| --- | --- |
